# Institutional operating figures in basic and applied sciences: Scientometric analysis of quantitative output benchmarking

**DOI:** 10.1186/1478-4505-6-6

**Published:** 2008-06-13

**Authors:** Beatrix Groneberg-Kloft, Cristian Scutaru, Carolin Kreiter, Silvana Kölzow, Axel Fischer, David Quarcoo

**Affiliations:** 1 Otto-Heubner-Centre, Charité, Free University Berlin and Humboldt-University, D-13353 Berlin, Germany; 2 Institute of Occupational Medicine, Charité, Free University Berlin and Humboldt-University, D-12207 Berlin, Germany

## Abstract

**Background:**

Institutional operating figures and benchmarking systems are important features for the implementation of efficacy in basic and applied sciences. They are needed for research evaluation and funding policy. However, the current policy settings for research evaluation urgently need review since there may be imbalances present in many areas.

**Methods:**

The present study assessed benchmarking of research output. By the use of large data bases research output was categorized and analyzed. Specific areas of major research activity were identified by comparing publication density on different organ systems and inter- and intrafield comparison was performed for selected countries.

**Results:**

Novel density-equalizing mappings were constructed that illustrate trends of publication activity and identify subsets of major interest in a total of 5,527,558 published items. A dichotomy was present between Western countries such as the US, UK or Germany and Asian countries such as Japan, China or South Korea concerning research focuses.

**Conclusion:**

The present study is the first large scale analysis of global research activity and output over the last 50 years. The presently described assessment of operating figures at the national and international level can be used to identify single areas of research that are heavily focused. Further research on qualitative output benchmarking is needed to improve current policy settings for research evaluation.

## Background

Economic progress is crucially dependent on advance in basic and applied research. The advance itself is directly related to intramural and even more to extramural governmental and non-governmental funding. Due to the importance of external funding for the advance of science, numerous statements [1-9], reviews [10,11] and original studies [12] exist that focus on funding trends. Also evaluation policies by major funding organizations are published annually. Due to the tight financial situation in many countries it is becoming increasingly difficult to provide solid monetary resources for both research and education. Therefore, acquisition of extramural grants from governmental and non-governmental institutions has become indispensable for all fields of research. In this respect, institutional operating figures and benchmarking systems are extremely important features to implement efficient funding. While tools to assess these features are known for many areas of research they are especially important in biomedical research. Here they are used to analyze funding schemes and to develop future funding policies. There is a remarkable amount of scientific literature present on institutional operating figures for biomedical areas which are heavily funded by governmental or industrial sponsors. These areas are i.e. neuroscience [13], cardiovascular medicine [14], gastroenterology [15], immunology [16], genetics [17], molecular biology [18-20] or stem cell research [21-23]. Next to the existing literature for these major fields of research there are also data available for smaller areas such as history of medicine [24], medical education [25], nursing sciences [26,27], reproductive health [28] or rehabilitation sciences [29,30].

Reviewing the existing policy in Europe [31] and general statements [32-36], it becomes clear that institutional operating figures and benchmarking systems are needed for research evaluation and funding policy.

The present study was performed to establish a first overview on global publication activities as a benchmark of quantitative research output. Due to the existence of multiple and advanced data bases, the area of biomedical research was chosen and publications related to single organs/systems were analyzed.

## Methods

Using two large databases (Scopus and Web of Science), biomedical research output was categorized with the numbers of published entries as an index marker for quantity of output. Quantities were analyzed with regard to three main characteristics: 1) organs 2) countries 3) publication dates. The below listed data bases were used.

### Scopus

This is the largest abstract and citation database of research literature and quality web sources. It is designed to find the information scientists need. Quick, easy and comprehensive, Scopus provides superior support of the literature research process. Updated daily, Scopus includes: Over 15,000 peer-reviewed titles from more than 4,000 publishers (500 Open Access journals, 700 conference proceedings, 600 trade publications), 29 million abstracts, 265 million references. The Scopus data base was used to construct charts with organ-country-specific publication benchmarks.

### Web of Science

This is an online academic database provided by the Thomson Institute for Scientific Information (ISI, license with Charité, Humboldt-University Berlin) [37,38]. It provides access to many databases and other resources including: Science Citation Index (SCI), Social Sciences Citation Index (SSCI), Arts & Humanities Citation Index (A&HCI), Index Chemicus, and Current Chemical Reactions, covering about 8,700 leading journals in science, technology, social sciences, and humanities.

#### Search strategies

**Figure 1:**
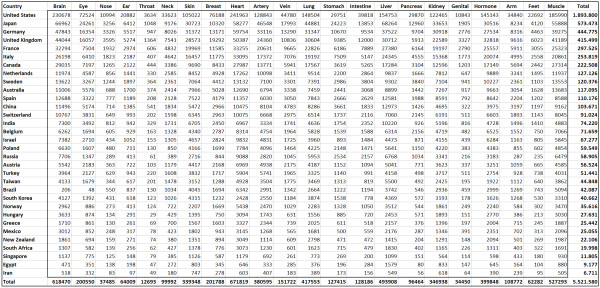
Total number of published items.

**Figure 2:**
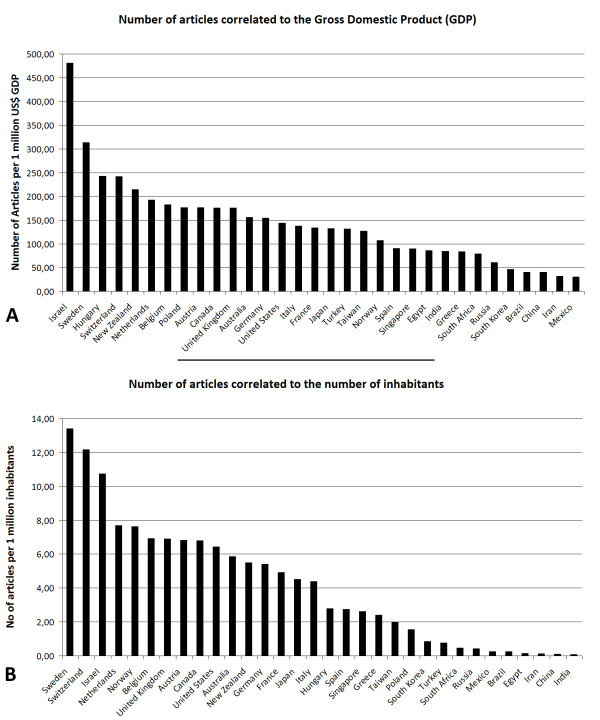
**Total number of published items in relation to the gross domestic product (GDP, A) and to the number of inhabitants (B).** Scopus data base search.

**Figure 3:**
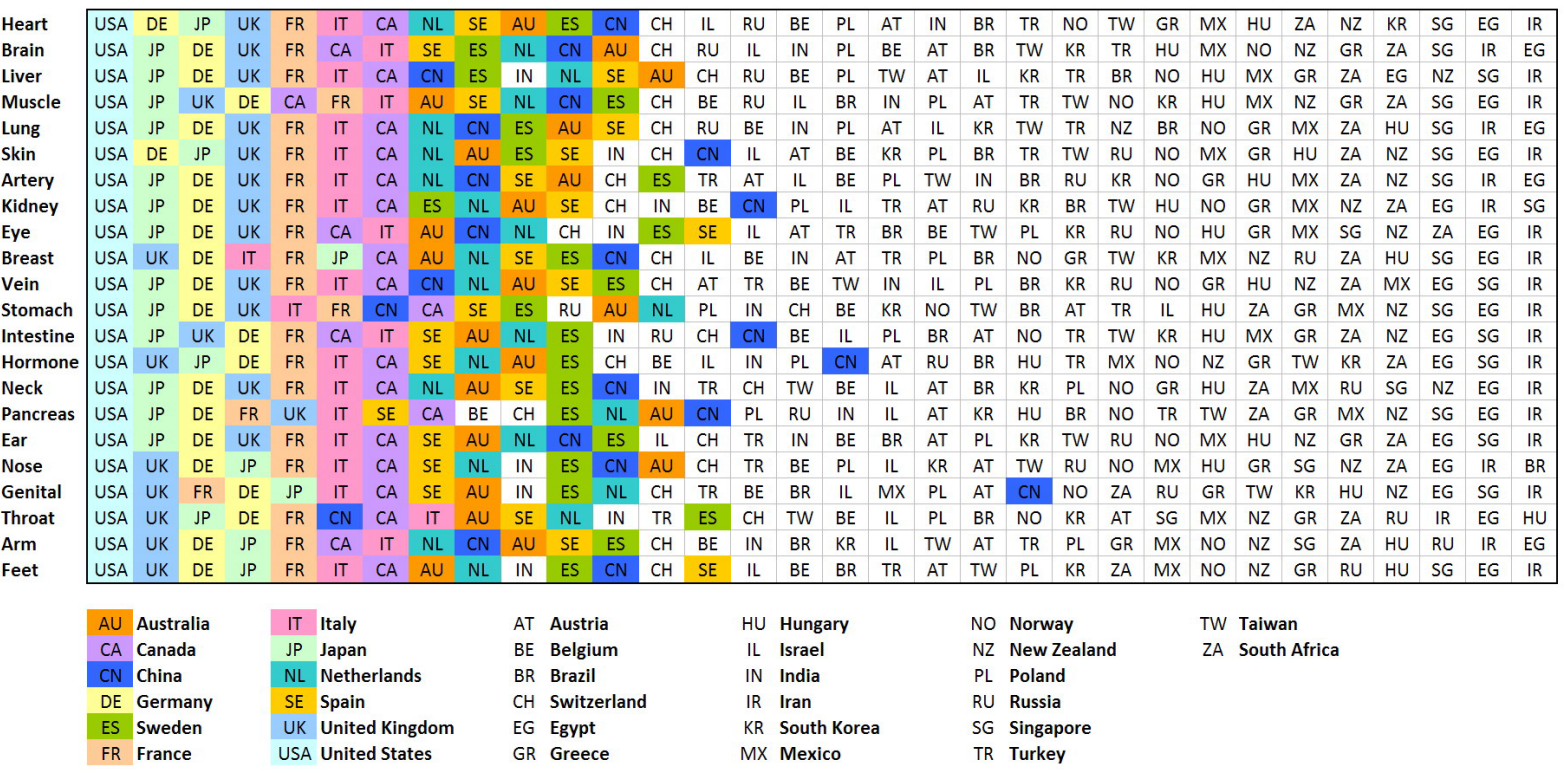
**Organ-specific and country-specific ranking of published items.** Scopus data base search.

For the different searches, the following terms joined together with Boolean operators, i.e. AND were used:

1) organs – the following terms were used to identify single organs: Brain, heart, artery, vein, lung, muscle, eye, nose, ear, throat, neck, skin, breast, stomach, intestine, pancreas, kidney, genital, hormone, arm, feet.

2) countries – while the search in the Web of Science was not restricted in order to calculate global density – equalizing maps, the Scopus searches were restricted to the following list of countries: United States, Germany, Japan, United Kingdom, Iran, Singapore, New Zeeland, Egypt, South Africa, Greece, Mexico, Hungary, Norway, Brazil, Turkey, South Korea, Israel, Austria, Taiwan, Spain, Poland, Belgium, Russia, Switzerland, Sweden, Australia, Netherlands, India, France, Italy, Canada, China.

3) Time span – the analyzed time spans are listed for each data set in the result section: Figure 1, 2, 3, 4: Scopus – all data base files included until the date of the retrieval (2007-09-30). Figures 5, 6, 7, 8, 9: Web of Science – data included between 1966 – 1976 and 1996 – 2006 (2007-11-12). Additional file 1: Scopus – data included between 1961–1970, 1971–1980, 1981–1990, 1991–2000, 2001–2007 (decade 2000–2010 not finalized) (2007-09-30).

**Figure 4:**
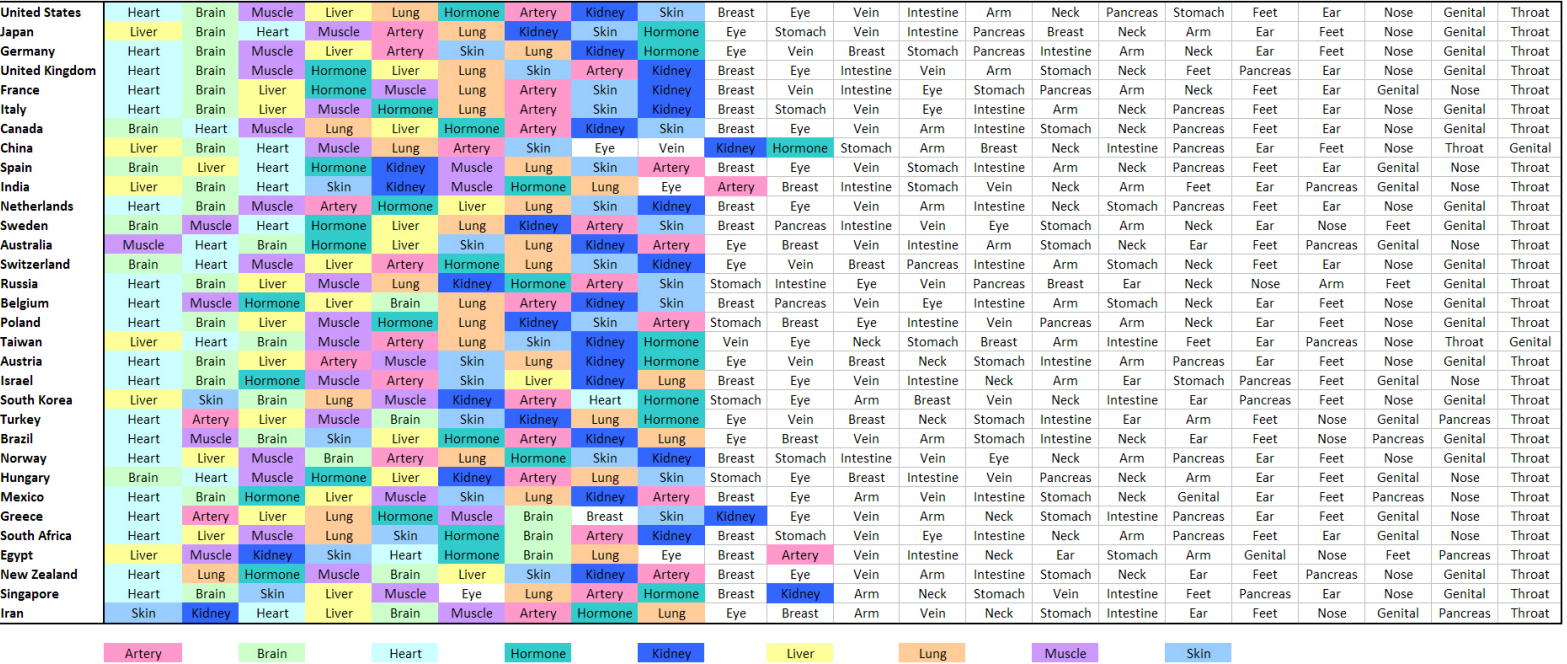
**International differences in focus of research.** Ranking of organs in each country. Scopus data base search.

**Figure 5:**
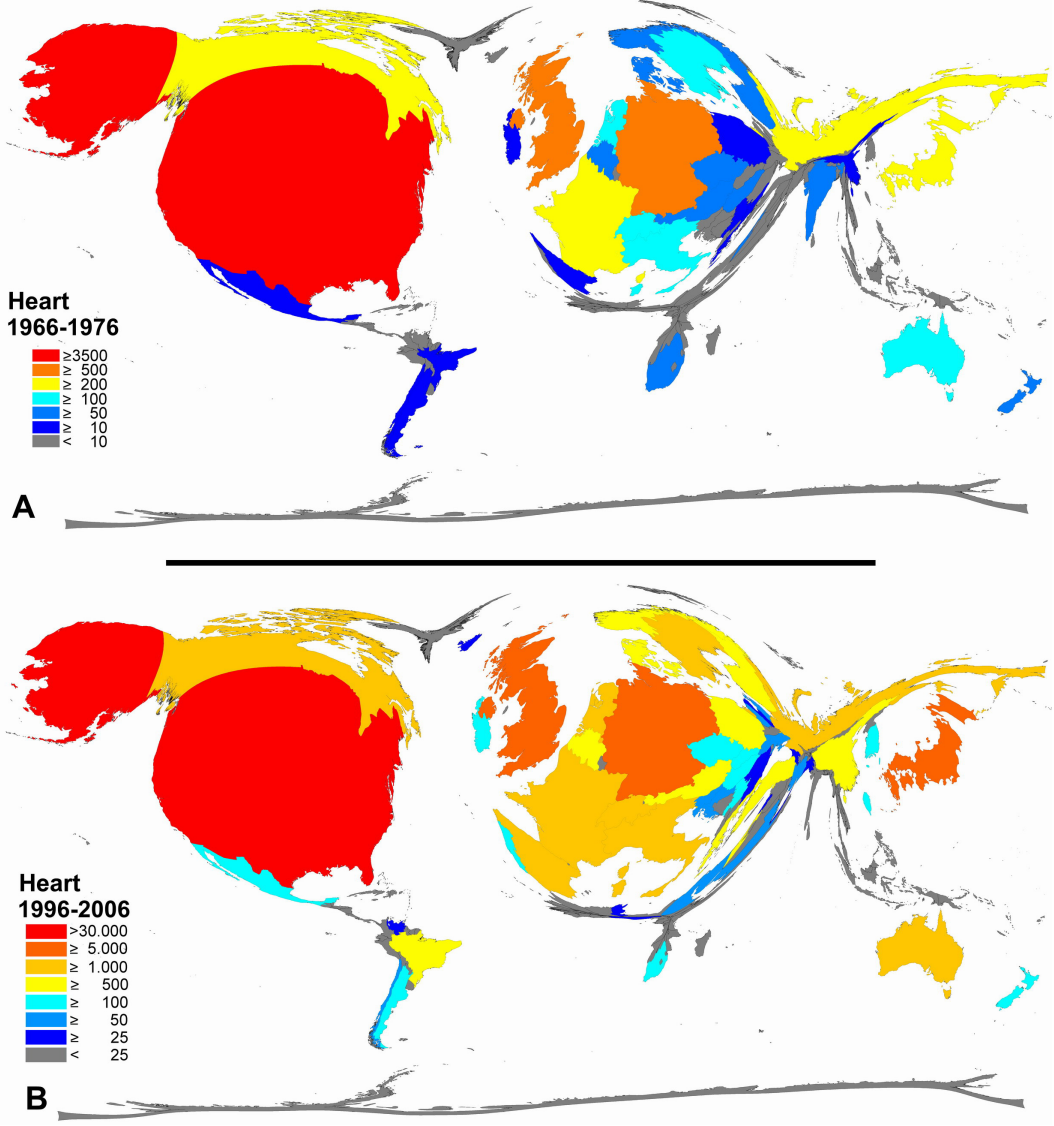
**Color-coded Density-equalizing mapping: Publication quantities for items related to the term "heart" in the two periods 1966–1976 and 1996–2006.** Web of Science data base search.

**Figure 6:**
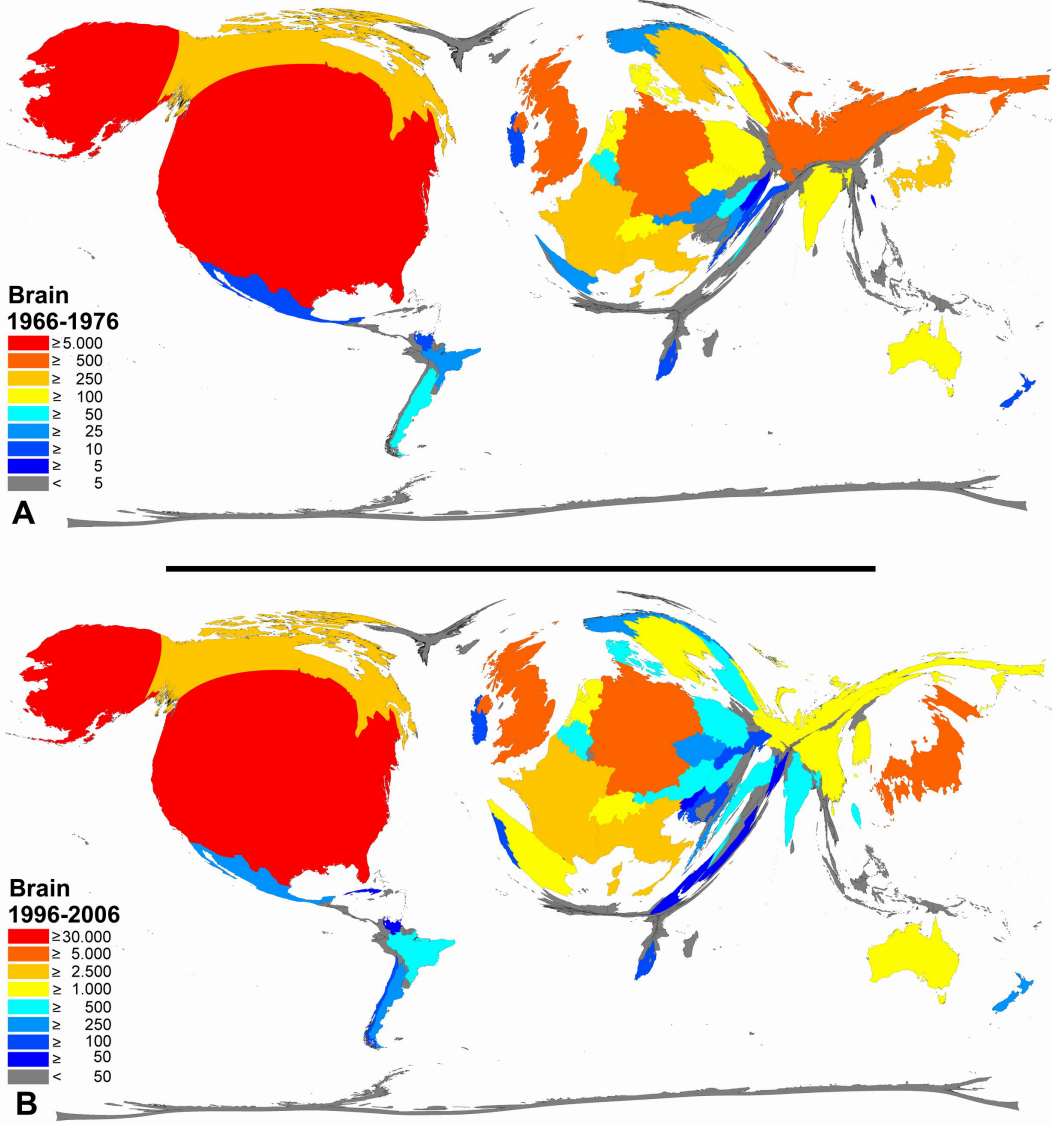
**Color-coded Density-equalizing mapping: Publication quantities for items related to the term "brain" in the two periods 1966–1976 and 1996–2006.** Web of Science data base search.

**Figure 7:**
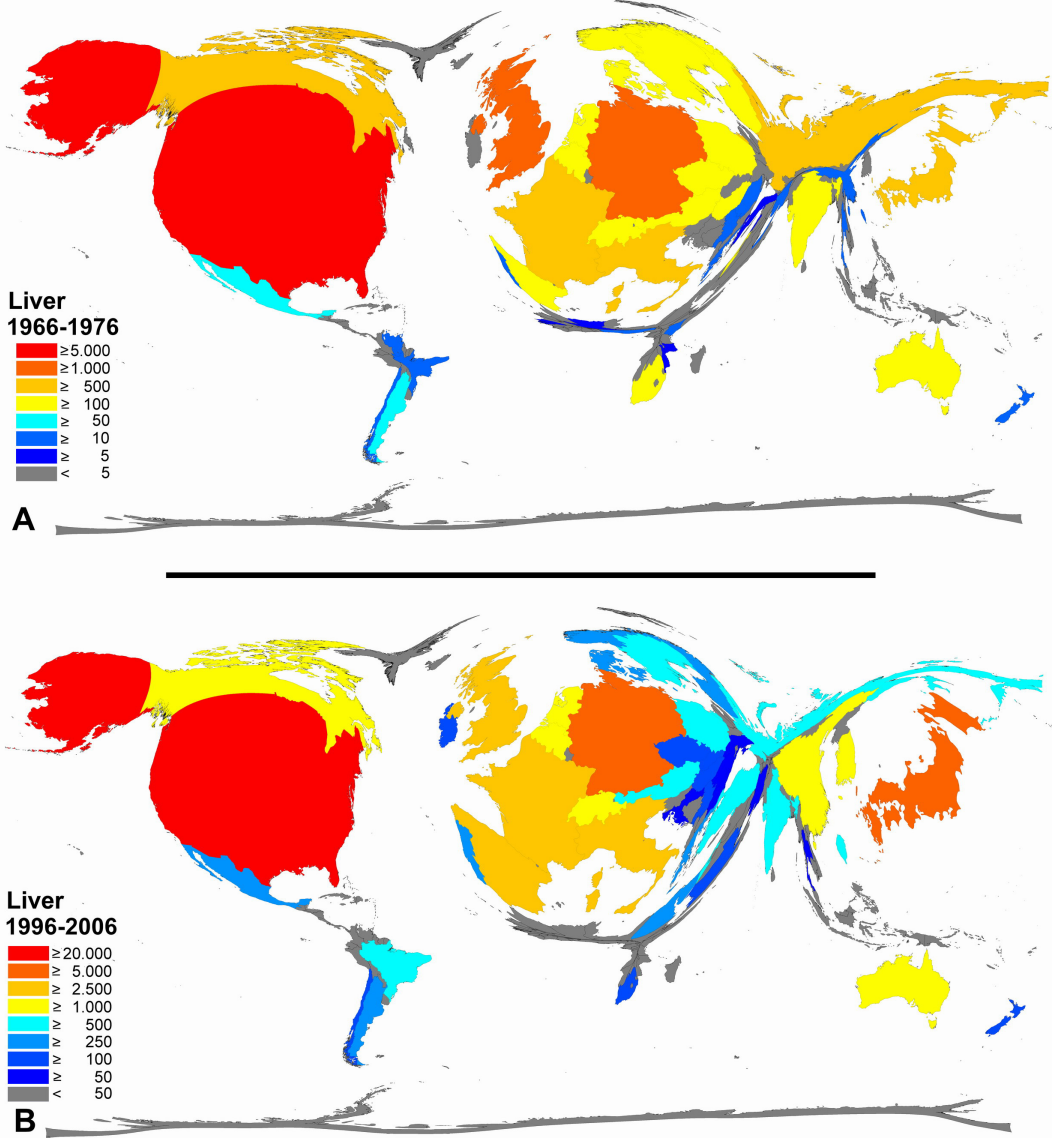
**Color-coded Density-equalizing mapping: Publication quantities for items related to the term "liver" in the two periods 1966–1976 and 1996–2006.** Web of Science data base search.

**Figure 8:**
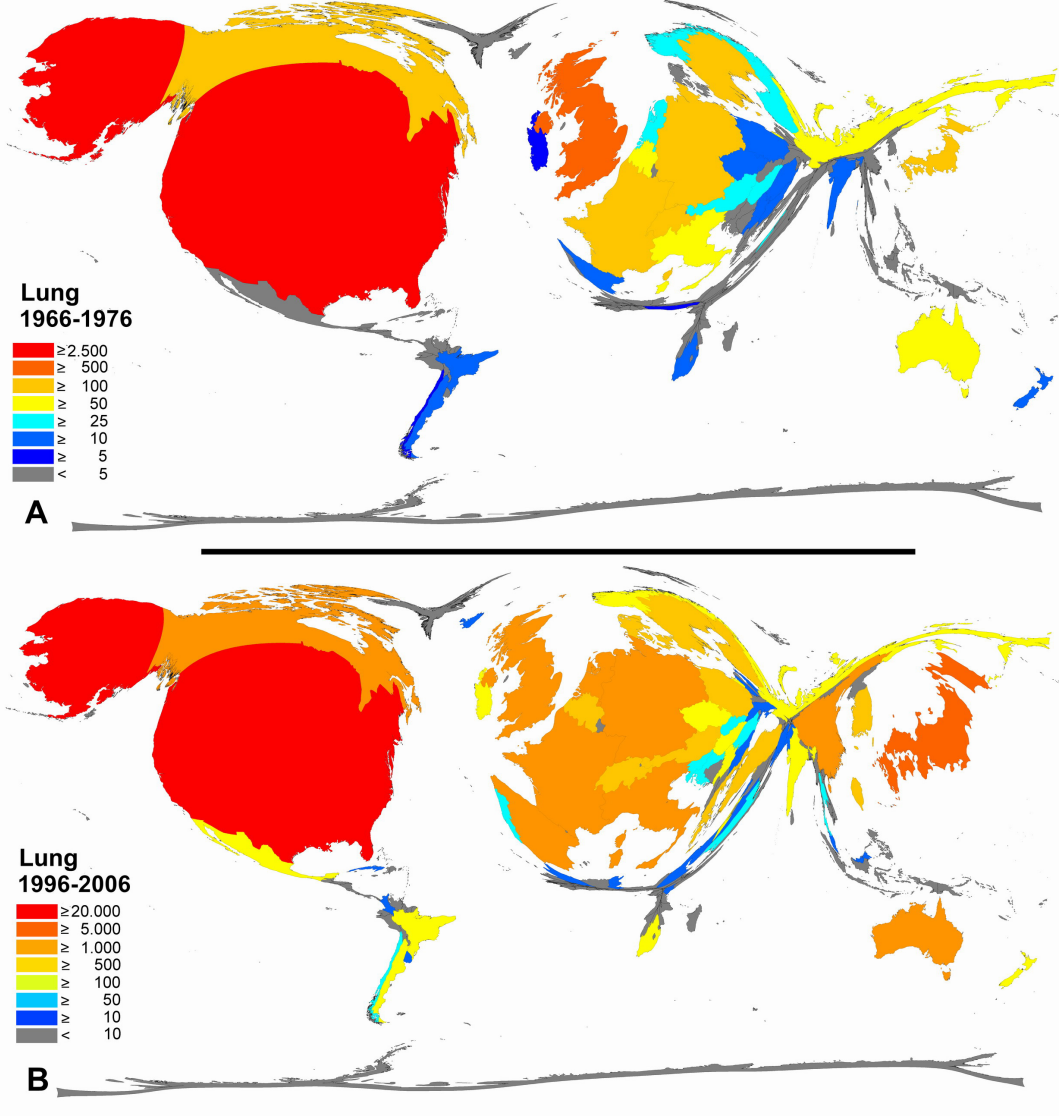
**Color-coded Density-equalizing mapping: Publication quantities for items related to the term "lung" in the two periods 1966–1976 and 1996–2006.** Web of Science data base search.

**Figure 9:**
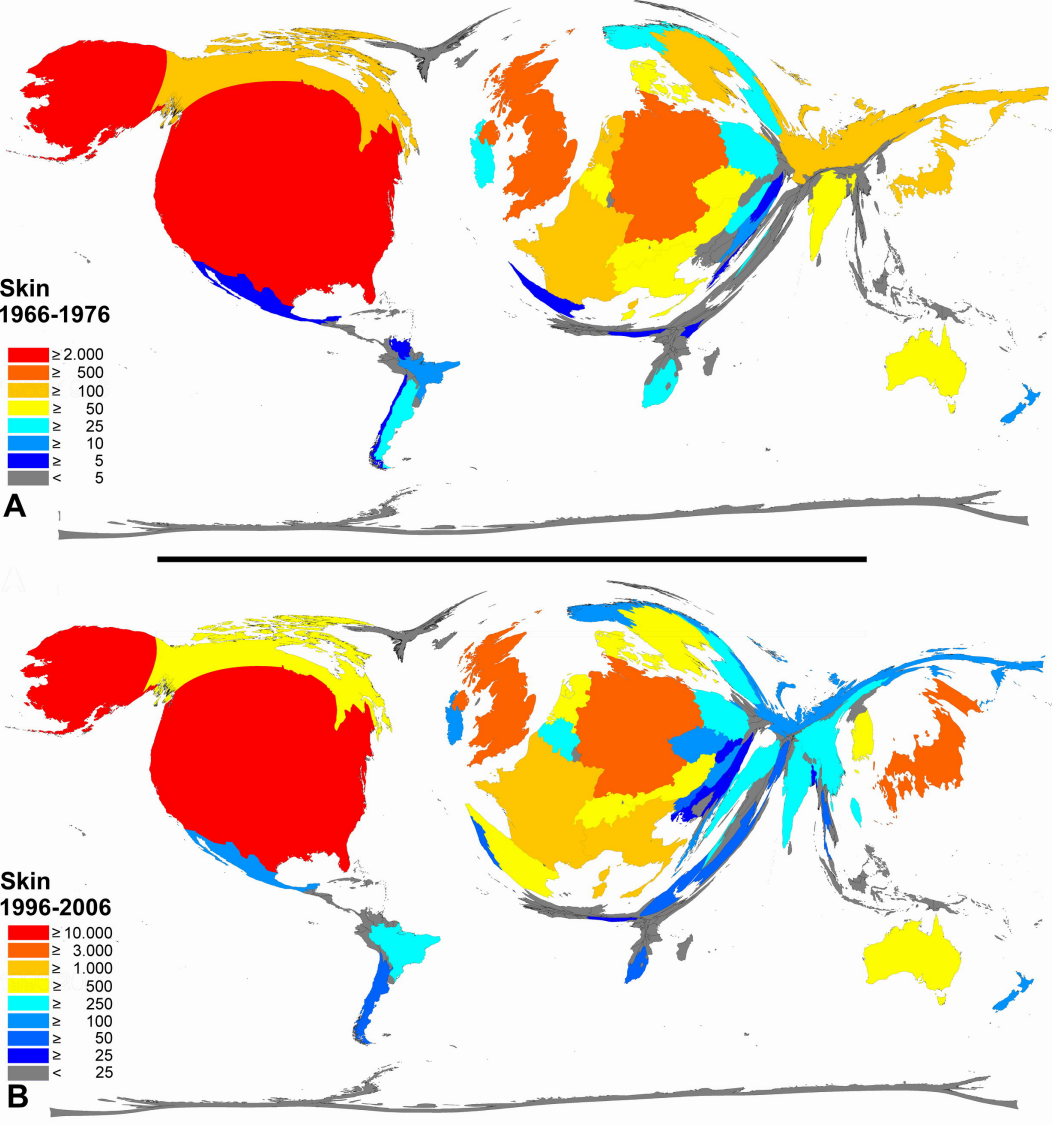
**Color-coded Density-equalizing mapping: Publication quantities for items related to the term "skin" in the two periods 1966–1976 and 1996–2006.** Web of Science data base search.

Total numbers of entries were also related to the parameters gross domestic product (GDP, supplied by the international monetary fund [39], Figure 2) and population numbers of 2007 (supplied by CIA World fact book [40], Figure 2).

#### Density-equalizing mapping

Density-equalizing mapping was used for visualization of data according to a recently published method. In brief, territories were re-sized according to a particular variable, i.e. the number of published items. For the re-sizing procedure the area of each country was scaled in proportion to its total number of published items regarding the organs heart, brain, liver, lung and skin. The specific calculations are based on Gastner and Newman's algorithm [41]. As data set, published items for each organ between 1966 – 1976 and 1996 – 2006 of the ISI Web data base were used.

## Results

### Total number of published items

Using the Scopus database, the number of published items was used as a benchmark of quantity of research output. A total of 5,527,558 published items were analyzed and large differences were found between single organs and single countries (Figure 1): The United States was found to be the most productive country with a total number of 1,893,800 published items. Japan ranked second with 573,473 items followed by Germany (444,775) and the United Kingdom (415,499). When the total number of published items is related to the gross domestic product (GDP) the ranking changes and Israel (1.), Sweden (2.), Hungary (3.), Switzerland (4.) and New Zealand (5.) are listed in the first positions (Figure 1). In this score, the US (14.), Japan (17.), Germany (13.) and UK (11.) are listed in later positions. When the total number of published items per country is related to the number of inhabitants, the first positions include Sweden (1.), Switzerland (2.), Israel (3.), Netherlands (4) and Norway (5.) while the US (10.), Japan (15.), Germany (13.) and UK (7.) are listed in later positions. (Figure 2).

### Country-specific output

When the different organs are related to the most productive countries, an apparent trend is seen for all organs that were analyzed in the present study (Figure 3): The most productive country for every organ is the United States. Then, Japan, UK and Germany follow on the second place. Color-coding demonstrates for the top ten countries that there is a similar country-specific ranking present for many countries. I.e. France is ranked number 5 in 18 out of 22 organ categories and Italy is ranked number 6 in 14 out of 22 organ categories.

Further detailed analysis on the organ-specific research output of different countries using 5 separate time spans from 1961–1970, 1971–1980, 1981–1990, 1991–2000 and 2001–2007 creates a vast amount of information [Additional file 1]. However, it demonstrates the same tendencies for each of these decades with no or only minor exceptions.

### International differences in focus of research

A remarkable difference is present in the individual focus of each country analyzed in the Scopus database. The United States has a clear ranking with the primary focus on studies related to heart (1), brain (2), Muscle (3), liver (4) and lung (5) (Figure 4). A similar focus is shared by countries such as the United Kingdom, Germany or Switzerland with an identical top 3 as indicated by color coding (Figure 4). However, the second most productive country Japan has its primary focus on articles related to the liver. Countries that also primarily focus on the liver are China, Taiwan, South Korea, India and Egypt (Figure 4).

A second step in analyzing international differences is to assess the research output activity over different time spans. The Additional file 2 shows the productivity development (published articles) of each country for every of the 22 organs between 1961–1970, 1971–1980, 1981–1990, 1991–2000 and 2001–2007. Strong increases in quantitative output are present for most countries and most organs [Additional file 2].

### Density-equalizing mapping

In a final step, a Web of Science analysis encompassing all countries and density equalizing algorithms were used to visualize global trends in quantitative research output. The two periods 1966–1976 and 1996–2006 were analyzed for the organs heart, brain, liver, lung and skin and transferred to cartograms. Color-graduation shows that there is a large difference in the overall number of published items related to the heart (Figure 5) with a factor of about 10 between the periods of 1966–1976 and 1996–2006. The proportion of the leading countries is similar in both periods. Only minor proportional changes can be found, i.e. for Brazil (increase) versus Argentina or India (decrease) versus China or Spain (increase) versus France.

When focusing on the published items related the brain similar trends can be seen with a relatively stable situation for the top productive countries US, UK, Japan and Germany and small increases for countries such as China, Spain or South Korea (Figure 6).

The density equalizing cartogram of the liver which is an organ of primary interest of many Asian countries demonstrates a relative increase for China and a decrease for Russia between 1966–1976 and 1996–2006 (Figure 7). Also, a relative increase of the proportion of Spain is present while South Africa's proportion decreased.

For articles related to the lung (Figure 8) and to the skin (Figure 9), a clear proportional increase between the periods of 1966–1976 and 1996–2006 is present for Japan, Spain and China while Russia (lung and skin) and South Africa (skin) slightly decrease in their proportion.

## Discussion

The present study was conducted to provide novel data on global scientific publication activities as a benchmark of research output. In this respect a focus was set primarily on the quantity over the past 50 years – not on the quality which is subject to further studies. Research in this area is of major importance since the policy settings for biomedical and medical research evaluation urgently need review. In the past, numerous scientific reviews and original publications have identified the primacy of quantity over quality as one of the most important threads. Research evaluation and policy projects in countries such as the USA or Australia have described the existence and nature of this problem extensively in their countries but there is still a lack concerning global data. Also, for Europe only little data is available [10,42,43].

The methodological basis of the study consists of database searches using the Scopus and Web of Science databases and the three characteristics 1) organs – to relate the data to clinical and biomedical fields, 2) countries – to provide a global overview of output activity and 3) publication dates – to assess changes over the time.

The range of analyzed scientific publications is unique. However, it should be realized that every database research houses limitations. In the present case, the definition of organ-related terms displays a major limitation since the list of organs (brain, heart, artery, vein, lung, muscle, eye, nose, ear, throat, neck, skin, breast, stomach, intestine, pancreas, kidney, genital, hormone, arm, and feet) can not be representative. Important aspects of diseases, symptoms, syndromes and clinical fields were not included in order to be able to delineate the enormous amount of data files. In this respect, it can be stated that i.e. published items related to blood disorders are not recognized through the search routines.

Also, the issue of linguistic differences and its effects on publication quantity should be addressed. In this respect, the present study included the analysis of publications in all languages included in the data bases. The majority of publications is published in English and it is difficult for non-English journals to get included in the data bases. Therefore, numerous scientific publications in languages other than English are not accessible by the present approach. This is a major bias. Therefore, English speaking countries such as the US, Canada or the UK have an advantage. However, it is generally accepted that scientists from non-English speaking countries in Europe and Asia publish their high quality research in scientific journals that use English as language.

Numerous interesting aspects are found in the present study. In this respect, the first analysis of total numbers of published items reveals a ranking of US, Japan, Germany, UK and France. This ranking parallels the results of many other research benchmarking systems with the US as the top-ranked country [44,45]. However, when the total number of published items is related to the GDP, the 3.3 fold differences between the top-ranked US (1,893,800 published items) and the second ranked Japan (573,473) completely changes and the US and Japan are listed at position 14 and 17, respectively, while countries such as Israel, Sweden and the Eastern European country Hungary lead the field. Similar changes are seen when the data are related to the number of inhabitants with the US and Japan being listed at position 10 and 15, respectively, with Sweden, Switzerland and Israel at the top 3 positions.

The benchmarking process can be subdivided into several fields. A division into indices for different organs makes sense in order to specify the clinical and research focus of single countries. When each organ is assessed for its relevance within each country, a relatively high homogeneity is present: The US ranks first for every organ followed by Japan, UK and Germany. Also for other countries, a high homogeneity is present (i.e. France is ranked number 5 in 18 out of 22 organ categories). Further separation into 5 separate periods from 1961–1970, 1971–1980, 1981–1990, 1991–2000 and 2001–2007 (online supplement file 1) demonstrates similar tendencies.

When individual country-specific organ research focuses are analyzed, continental differences become evident. Western countries such as the US have a clear ranking with the primary focus on articles related to heart (1), brain (2). Muscle (3), liver (4) and lung (5) this focus is more or less similar in the United Kingdom, Germany or Switzerland with an identical top 3. The reason is a similar research and funding policy with a focus on diseases related to the cardiovascular and the nervous systems as indicated in other studies [46]. These diseases also constitute a major burden of disease [47].

In contrast, Asian countries such as Japan, China, South Korea or India have their primary focus on articles related to the liver. Analyzing the burden of disease in these countries, liver related diseases are not at first position. Interestingly, countries such as Australia that are known to have a critical problem with skin cancer due to sun exposure [48] do not have skin-related publications in a top focus.

A further large analysis (online supplement 2) screened differences in the research output with regard to the five periods 1961–1970, 1971–1980, 1981–1990, 1991–2000 and 2001–2007. This data set illustrates a general increase in quantitative output. In this respect, the period of 2001–2007 can not be fully compared to the previous decades due to a lack of 3 years of research.

The final step of this study encompassed a Web of Science analysis using previously published density equalizing algorithms in order to visualize global trends in quantitative research output. Large increases in overall numbers of published items were present. This is in accordance to commonly known trends [49]. The proportions of leading countries is more or less similar in the two time periods (1966–1976 and 1996–2006) and only minor proportional changes can were found. In this respect, a general increase was present for the countries Spain and China in relation to their neighboring countries.

Summarizing these different data analysis approaches, the current study allows to quantify research output globally with regard to a list of 22 relevant organs and selected time periods. The overall result that the US is the predominant country in every quantitative biomedical research outcome parameter is a generally known fact. These finding points to a valid choose of search terms. However, the present approach also provides a broad spectrum of new information. I.e. the high level of homogeneity that is present in the most productive countries concerning their focus of research with similar organs being listed in the top ten. In this respect, a dichotomy is present: Whereas western countries have a clear focus on heart- and brain-related publications, the Asian countries all primarily focused on publications related to the organ liver. This can not be attributed to the burden of disease which is dominated by cardiovascular, neurovascular, respiratory and infectious diseases [47].

Whereas the present study generated a large set of data it needs to be taken into account that this data only describes the quantitative output. Benchmarking systems should also estimate qualitative aspects. The commonly used marker for research quality is the citation index and future studies need to assess this feature. However, it can be estimated from the present data analysis that citation indices will be high for those organs that also have the highest numbers of published items since a citation can only be traced in a published item and a high number of published items also indicate a high number of citations.

In summary, the present study encompasses a novel approach to assess output in research. It was conducted to provide and interpret quantitative benchmarking data on global publication activities. While the US was the leading country in all relevant categories followed by Japan, an interesting dichotomy was present between Western countries such as the US, UK or Germany and Asian countries such as Japan, China or South Korea concerning their primary research interests. Benchmarking systems basing on research output can be used to identify individual and regional differences. However, they need to be backed up by qualitative benchmarks and socioeconomic data to improve international policy settings for research evaluation.

## Competing interests

The authors declare that they have no competing interests.

## Authors' contributions

BGK conceived of the study, participated in the design and co-ordination of the study, performed the analysis, and drafted and prepared the manuscript, CS, CK and SK participated in the analysis, AF and DQ helped to interpret the data. All authors read and approved the final manuscript.

## Supplementary Material

Additional file 1 Number of published items for every organ over 5 time periods. In this file a Scopus – data research was performed for every organ. The research included the periods between 1961–1970, 1971–1980, 1981–1990, 1991–2000, and 2001–2007 (decade 2000–2010 not finalized). Click here for file

Additional file 2 Number of published items for every organ over 5 time periods. In this file a Scopus – data research was performed for every organ. The research included the periods between 1961–1970, 1971–1980, 1981–1990, 1991–2000, and 2001–2007 (decade 2000–2010 not finalized). Click here for file
