# Supplementary material for: Institutional operating figures in basic and applied sciences: Scientometric analysis of quantitative output benchmarking
Source: Health Res Policy Syst. 2008 Jun 13;6:6. doi: 10.1186/1478-4505-6-6 (PMC2459159; doi:10.1186/1478-4505-6-6)
Supplement: Additional file 1 — Number of published items for every organ over 5 time periods. In this file a Scopus – data research was performed for every organ. The research included the periods between 1961–1970, 1971–1980, 1981–1990, 1991–2000, and 2001–2007 (decade 2000–2010 not finalized). [file 1478-4505-6-6-S1.doc]

**Number of published items for the search term:**

| **Brain** |  |  |  |  |  |
| --- | --- | --- | --- | --- | --- |
|  |  |  | **Year** |  |  |
| **Country** | **1961-1970** | **1971-1980** | **1981-1990** | **1991-2000** | **2001-2007** |
| **United States** | 116 | 31121 | 51568 | 83437 | 64436 |
| **Japan** | 2 | 7057 | 13724 | 27497 | 18682 |
| **Germany** | 1 | 6131 | 8089 | 17128 | 16494 |
| **United Kingdom** | 7 | 5799 | 8762 | 15623 | 13853 |
| **France** | 1 | 4499 | 6961 | 11826 | 9007 |
| **Canada** | 4 | 3319 | 6911 | 10083 | 8718 |
| **Italy** | 2 | 2963 | 5115 | 9500 | 8618 |
| **Sweden** | 1 | 1892 | 3145 | 4915 | 3669 |
| **Spain** | 0 | 811 | 1693 | 5165 | 5019 |
| **Netherlands** | 0 | 1105 | 2105 | 4241 | 4523 |
| **China** | 0 | 40 | 478 | 2310 | 8668 |
| **Australia** | 1 | 928 | 1783 | 3919 | 4375 |
| **Switzerland** | 1 | 1316 | 1959 | 3773 | 3718 |
| **Russia** | 0 | 4844 | 2138 | 526 | 198 |
| **Israel** | 2 | 722 | 1419 | 2565 | 2674 |
| **India** | 2 | 1266 | 1397 | 2103 | 2532 |
| **Poland** | 2 | 1887 | 991 | 1580 | 2170 |
| **Belgium** | 0 | 822 | 1101 | 2189 | 2150 |
| **Austria** | 0 | 762 | 938 | 1921 | 1921 |
| **Brazil** | 4 | 360 | 306 | 1171 | 2506 |
| **Taiwan** | 0 | 108 | 306 | 1682 | 2037 |
| **South Korea** | 0 | 47 | 114 | 1273 | 2693 |
| **Turkey** | 0 | 99 | 188 | 1261 | 2416 |
| **Hungary** | 0 | 724 | 684 | 1091 | 1134 |
| **Mexico** | 1 | 352 | 457 | 1002 | 1200 |
| **Norway** | 0 | 456 | 595 | 953 | 958 |
| **New Zealand** | 0 | 141 | 246 | 737 | 737 |
| **Greece** | 0 | 55 | 179 | 543 | 933 |
| **South Africa** | 0 | 223 | 335 | 365 | 384 |
| **Singapore** | 0 | 36 | 80 | 345 | 676 |
| **Iran** | 0 | 35 | 26 | 60 | 397 |
| **Egypt** | 0 | 89 | 65 | 133 | 184 |

**Number of published items for the search term:**

| **Eye** |  |  |  |  |  |
| --- | --- | --- | --- | --- | --- |
|  |  |  | **Year** |  |  |
| **Country** | **1961-1970** | **1971-1980** | **1981-1990** | **1991-2000** | **2001-2007** |
| **United States** | 12 | 9476 | 15781 | 26511 | 20744 |
| **Japan** | 1 | 2884 | 3151 | 10079 | 8146 |
| **Germany** | 1 | 2158 | 2976 | 5922 | 5297 |
| **United Kingdom** | 0 | 1824 | 2797 | 5924 | 5512 |
| **France** | 0 | 1022 | 1351 | 2670 | 2461 |
| **Canada** | 0 | 700 | 1353 | 2552 | 2592 |
| **Italy** | 0 | 712 | 1112 | 2388 | 2198 |
| **Australia** | 0 | 415 | 783 | 2071 | 2307 |
| **China** | 0 | 18 | 142 | 1049 | 3965 |
| **Netherlands** | 0 | 501 | 994 | 1641 | 1451 |
| **Switzerland** | 0 | 523 | 786 | 1317 | 1205 |
| **India** | 0 | 306 | 335 | 1061 | 1790 |
| **Spain** | 0 | 191 | 272 | 1273 | 1586 |
| **Sweden** | 0 | 436 | 763 | 1189 | 879 |
| **Israel** | 0 | 334 | 517 | 918 | 941 |
| **Austria** | 0 | 320 | 422 | 664 | 777 |
| **Turkey** | 0 | 15 | 56 | 665 | 1391 |
| **Brazil** | 0 | 176 | 96 | 478 | 1135 |
| **Belgium** | 0 | 277 | 271 | 586 | 560 |
| **Taiwan** | 0 | 55 | 74 | 581 | 969 |
| **Poland** | 0 | 564 | 146 | 345 | 552 |
| **South Korea** | 0 | 14 | 15 | 427 | 936 |
| **Russia** | 0 | 898 | 323 | 86 | 40 |
| **Norway** | 0 | 127 | 177 | 305 | 277 |
| **Hungary** | 0 | 221 | 121 | 220 | 312 |
| **Greece** | 0 | 59 | 74 | 309 | 419 |
| **Mexico** | 1 | 71 | 78 | 342 | 360 |
| **Singapore** | 0 | 31 | 21 | 259 | 464 |
| **New Zealand** | 0 | 60 | 102 | 217 | 315 |
| **South Africa** | 0 | 83 | 115 | 192 | 192 |
| **Egypt** | 0 | 84 | 40 | 73 | 154 |
| **Iran** | 0 | 9 | 9 | 38 | 276 |

**Number of published items for the search term:**

| **Nose** |  |  |  |  |  |
| --- | --- | --- | --- | --- | --- |
|  |  |  | **Year** |  |  |
| **Country** | **1961-1970** | **1971-1980** | **1981-1990** | **1991-2000** | **2001-2007** |
| **United States** | 3 | 1720 | 2754 | 3787 | 2730 |
| **United Kingdom** | 0 | 450 | 771 | 1374 | 1000 |
| **Germany** | 0 | 646 | 640 | 1099 | 941 |
| **Japan** | 0 | 340 | 806 | 1362 | 748 |
| **France** | 0 | 379 | 425 | 645 | 483 |
| **Italy** | 0 | 208 | 383 | 628 | 604 |
| **Canada** | 0 | 185 | 340 | 454 | 286 |
| **Sweden** | 0 | 144 | 354 | 476 | 270 |
| **Netherlands** | 0 | 89 | 199 | 321 | 247 |
| **India** | 0 | 160 | 108 | 221 | 323 |
| **Spain** | 0 | 148 | 90 | 293 | 246 |
| **China** | 0 | 5 | 42 | 138 | 529 |
| **Australia** | 1 | 79 | 120 | 257 | 231 |
| **Switzerland** | 0 | 100 | 106 | 246 | 197 |
| **Turkey** | 0 | 19 | 17 | 180 | 413 |
| **Belgium** | 0 | 109 | 88 | 225 | 183 |
| **Poland** | 0 | 161 | 66 | 104 | 149 |
| **Israel** | 0 | 47 | 124 | 144 | 119 |
| **South Korea** | 0 | 9 | 10 | 138 | 274 |
| **Austria** | 0 | 76 | 75 | 114 | 98 |
| **Taiwan** | 0 | 9 | 24 | 144 | 167 |
| **Russia** | 0 | 222 | 57 | 10 | 0 |
| **Norway** | 0 | 55 | 56 | 91 | 71 |
| **Mexico** | 0 | 33 | 47 | 98 | 70 |
| **Hungary** | 0 | 49 | 12 | 37 | 36 |
| **Greece** | 0 | 7 | 10 | 53 | 60 |
| **Singapore** | 0 | 18 | 11 | 43 | 53 |
| **New Zealand** | 0 | 17 | 30 | 59 | 53 |
| **South Africa** | 0 | 33 | 38 | 33 | 35 |
| **Egypt** | 0 | 35 | 27 | 36 | 40 |
| **Iran** | 0 | 9 | 5 | 12 | 57 |
| **Brazil** | 0 | 98 | 50 | 166 | 236 |

**Number of published items for the search term:**

| **Ear** |  |  |  |  |  |
| --- | --- | --- | --- | --- | --- |
|  |  |  | **Year** |  |  |
| **Country** | **1961-1970** | **1971-1980** | **1981-1990** | **1991-2000** | **2001-2007** |
| **United States** | 3 | 3276 | 5000 | 7208 | 5395 |
| **Japan** | 0 | 689 | 1481 | 2522 | 1720 |
| **Germany** | 0 | 1022 | 1013 | 1851 | 1631 |
| **United Kingdom** | 1 | 645 | 1154 | 1955 | 1519 |
| **France** | 0 | 521 | 640 | 1017 | 796 |
| **Italy** | 0 | 313 | 498 | 676 | 700 |
| **Canada** | 1 | 321 | 450 | 705 | 645 |
| **Sweden** | 0 | 337 | 602 | 594 | 364 |
| **Australia** | 5 | 273 | 290 | 570 | 562 |
| **Netherlands** | 0 | 135 | 298 | 554 | 454 |
| **China** | 0 | 3 | 69 | 269 | 1044 |
| **Spain** | 0 | 93 | 103 | 579 | 414 |
| **Israel** | 0 | 122 | 250 | 341 | 339 |
| **Switzerland** | 9 | 168 | 158 | 342 | 316 |
| **Turkey** | 0 | 27 | 15 | 233 | 668 |
| **India** | 0 | 156 | 83 | 263 | 440 |
| **Belgium** | 0 | 163 | 153 | 347 | 266 |
| **Brazil** | 0 | 64 | 67 | 295 | 411 |
| **Austria** | 2 | 156 | 139 | 211 | 214 |
| **Poland** | 2 | 224 | 86 | 129 | 270 |
| **South Korea** | 0 | 5 | 12 | 147 | 454 |
| **Taiwan** | 1 | 14 | 32 | 279 | 291 |
| **Russia** | 9 | 318 | 68 | 10 | 8 |
| **Norway** | 1 | 84 | 68 | 150 | 110 |
| **Mexico** | 0 | 36 | 45 | 115 | 121 |
| **Hungary** | 2 | 73 | 37 | 92 | 87 |
| **New Zealand** | 0 | 28 | 50 | 96 | 97 |
| **Greece** | 0 | 18 | 18 | 99 | 126 |
| **South Africa** | 0 | 28 | 50 | 98 | 80 |
| **Egypt** | 1 | 28 | 40 | 57 | 72 |
| **Singapore** | 0 | 8 | 8 | 64 | 68 |
| **Iran** | 0 | 5 | 3 | 22 | 67 |

**Number of published items for the search term:**

| **Throat** |  |  |  |  |  |
| --- | --- | --- | --- | --- | --- |
|  |  |  | **Year** |  |  |
| **Country** | **1961-1970** | **1971-1980** | **1981-1990** | **1991-2000** | **2001-2007** |
| **United States** | 11 | 459 | 772 | 1287 | 1105 |
| **United Kingdom** | 2 | 139 | 243 | 495 | 485 |
| **Japan** | 0 | 84 | 134 | 432 | 398 |
| **Germany** | 5 | 191 | 137 | 298 | 316 |
| **France** | 0 | 90 | 83 | 219 | 214 |
| **China** | 0 | 0 | 24 | 89 | 428 |
| **Canada** | 0 | 36 | 67 | 169 | 172 |
| **Italy** | 0 | 50 | 96 | 122 | 139 |
| **Australia** | 0 | 32 | 35 | 124 | 183 |
| **Sweden** | 0 | 53 | 82 | 120 | 109 |
| **Netherlands** | 3 | 16 | 30 | 109 | 172 |
| **India** | 0 | 63 | 40 | 90 | 136 |
| **Turkey** | 0 | 3 | 6 | 55 | 156 |
| **Spain** | 0 | 7 | 12 | 96 | 93 |
| **Switzerland** | 1 | 21 | 31 | 66 | 83 |
| **Taiwan** | 0 | 8 | 6 | 88 | 99 |
| **Belgium** | 0 | 39 | 12 | 63 | 49 |
| **Israel** | 0 | 13 | 22 | 63 | 55 |
| **Poland** | 0 | 19 | 9 | 27 | 75 |
| **Brazil** | 0 | 9 | 7 | 44 | 70 |
| **Norway** | 0 | 14 | 14 | 53 | 43 |
| **South Korea** | 0 | 0 | 2 | 27 | 94 |
| **Austria** | 0 | 15 | 13 | 39 | 36 |
| **Singapore** | 0 | 8 | 12 | 26 | 33 |
| **Mexico** | 1 | 5 | 9 | 25 | 38 |
| **New Zealand** | 0 | 12 | 14 | 24 | 24 |
| **Greece** | 0 | 4 | 7 | 23 | 35 |
| **South Africa** | 0 | 9 | 13 | 16 | 24 |
| **Russia** | 7 | 38 | 14 | 1 | 1 |
| **Iran** | 0 | 7 | 0 | 9 | 33 |
| **Egypt** | 0 | 10 | 4 | 15 | 18 |
| **Hungary** | 1 | 9 | 1 | 10 | 8 |

**Number of published items for the search term:**

| **Neck** |  |  |  |  |  |
| --- | --- | --- | --- | --- | --- |
|  |  |  | **Year** |  |  |
| **Country** | **1961-1970** | **1971-1980** | **1981-1990** | **1991-2000** | **2001-2007** |
| **United States** | 25 | 3915 | 6962 | 12067 | 10654 |
| **Japan** | 5 | 802 | 1426 | 3600 | 3343 |
| **Germany** | 69 | 1050 | 1317 | 2704 | 2886 |
| **United Kingdom** | 13 | 632 | 1324 | 2783 | 2789 |
| **France** | 3 | 736 | 893 | 1649 | 1551 |
| **Italy** | 6 | 467 | 760 | 1588 | 1821 |
| **Canada** | 2 | 298 | 592 | 1168 | 1326 |
| **Netherlands** | 2 | 103 | 312 | 1018 | 1150 |
| **Australia** | 0 | 140 | 282 | 926 | 1066 |
| **Sweden** | 2 | 193 | 423 | 941 | 802 |
| **Spain** | 1 | 151 | 135 | 848 | 993 |
| **China** | 0 | 1 | 70 | 352 | 1411 |
| **India** | 2 | 178 | 143 | 465 | 943 |
| **Turkey** | 0 | 19 | 21 | 376 | 1192 |
| **Switzerland** | 7 | 206 | 205 | 542 | 638 |
| **Taiwan** | 0 | 16 | 65 | 579 | 818 |
| **Belgium** | 1 | 129 | 193 | 473 | 532 |
| **Israel** | 1 | 107 | 220 | 466 | 511 |
| **Austria** | 8 | 141 | 209 | 410 | 411 |
| **Brazil** | 1 | 68 | 36 | 326 | 603 |
| **South Korea** | 0 | 8 | 37 | 251 | 730 |
| **Poland** | 3 | 181 | 78 | 150 | 438 |
| **Norway** | 1 | 85 | 111 | 285 | 240 |
| **Greece** | 1 | 29 | 38 | 227 | 405 |
| **Hungary** | 8 | 113 | 54 | 109 | 145 |
| **South Africa** | 0 | 58 | 117 | 145 | 107 |
| **Mexico** | 0 | 47 | 47 | 153 | 176 |
| **Russia** | 9 | 251 | 97 | 24 | 8 |
| **Singapore** | 0 | 21 | 17 | 141 | 206 |
| **New Zealand** | 0 | 29 | 53 | 147 | 151 |
| **Egypt** | 0 | 37 | 27 | 95 | 113 |
| **Iran** | 1 | 12 | 3 | 19 | 145 |

**Number of published items for the search term:**

| **Skin** |  |  |  |  |  |
| --- | --- | --- | --- | --- | --- |
|  |  |  | **Year** |  |  |
| **Country** | **1961-1970** | **1971-1980** | **1981-1990** | **1991-2000** | **2001-2007** |
| **United States** | 192 | 16192 | 26558 | 35123 | 26957 |
| **Germany** | 503 | 5181 | 6035 | 10195 | 9458 |
| **Japan** | 19 | 3055 | 6702 | 11590 | 9357 |
| **United Kingdom** | 49 | 4313 | 7018 | 9549 | 7644 |
| **France** | 12 | 2794 | 4092 | 7313 | 5758 |
| **Italy** | 27 | 1803 | 3261 | 5959 | 5407 |
| **Canada** | 10 | 1199 | 2088 | 3348 | 3045 |
| **Netherlands** | 20 | 783 | 1779 | 3101 | 2769 |
| **Australia** | 5 | 850 | 1403 | 2847 | 2861 |
| **Spain** | 2 | 465 | 761 | 3068 | 3226 |
| **Sweden** | 17 | 957 | 1895 | 2454 | 1741 |
| **India** | 12 | 993 | 1196 | 1862 | 2642 |
| **Switzerland** | 28 | 917 | 1198 | 2201 | 2001 |
| **China** | 1 | 25 | 338 | 1080 | 4028 |
| **Israel** | 10 | 526 | 1112 | 1634 | 1375 |
| **Austria** | 42 | 850 | 808 | 1373 | 1344 |
| **Belgium** | 3 | 628 | 857 | 1477 | 1375 |
| **South Korea** | 0 | 167 | 229 | 1197 | 2722 |
| **Poland** | 14 | 1094 | 508 | 804 | 1746 |
| **Brazil** | 4 | 386 | 371 | 1211 | 2073 |
| **Turkey** | 0 | 70 | 96 | 1079 | 2587 |
| **Taiwan** | 1 | 94 | 238 | 1294 | 1525 |
| **Russia** | 52 | 1812 | 609 | 177 | 66 |
| **Norway** | 2 | 345 | 571 | 668 | 621 |
| **Mexico** | 1 | 249 | 294 | 645 | 613 |
| **Greece** | 1 | 81 | 142 | 498 | 845 |
| **Hungary** | 19 | 461 | 212 | 338 | 365 |
| **South Africa** | 4 | 241 | 302 | 452 | 379 |
| **New Zealand** | 0 | 153 | 236 | 475 | 487 |
| **Singapore** | 2 | 52 | 148 | 376 | 548 |
| **Egypt** | 6 | 119 | 106 | 226 | 346 |
| **Iran** | 1 | 63 | 28 | 114 | 541 |

**Number of published items for the search term:**

| **Breast** |  |  |  |  |  |
| --- | --- | --- | --- | --- | --- |
|  |  |  | **Year** |  |  |
| **Country** | **1961-1970** | **1971-1980** | **1981-1990** | **1991-2000** | **2001-2007** |
| **United States** | 9 | 7650 | 12929 | 27448 | 28152 |
| **United Kingdom** | 3 | 1952 | 3738 | 7108 | 6491 |
| **Germany** | 35 | 1691 | 2192 | 4306 | 4947 |
| **Italy** | 1 | 931 | 2475 | 4388 | 3980 |
| **France** | 0 | 1103 | 2190 | 4351 | 3941 |
| **Japan** | 4 | 1012 | 1704 | 3838 | 3762 |
| **Canada** | 2 | 522 | 1230 | 3029 | 3650 |
| **Australia** | 0 | 315 | 752 | 1792 | 2169 |
| **Netherlands** | 1 | 246 | 727 | 1928 | 2026 |
| **Sweden** | 1 | 388 | 828 | 1540 | 1655 |
| **Spain** | 0 | 195 | 387 | 1571 | 2026 |
| **China** | 0 | 4 | 152 | 613 | 2197 |
| **Switzerland** | 2 | 305 | 464 | 1066 | 1126 |
| **Israel** | 0 | 215 | 465 | 1013 | 1131 |
| **Belgium** | 0 | 298 | 447 | 947 | 1095 |
| **India** | 1 | 291 | 354 | 697 | 1107 |
| **Austria** | 2 | 224 | 370 | 695 | 877 |
| **Turkey** | 0 | 18 | 47 | 395 | 1257 |
| **Poland** | 0 | 204 | 136 | 326 | 1033 |
| **Brazil** | 0 | 158 | 156 | 450 | 930 |
| **Norway** | 0 | 88 | 276 | 623 | 682 |
| **Greece** | 0 | 45 | 99 | 513 | 946 |
| **Taiwan** | 0 | 9 | 41 | 411 | 827 |
| **South Korea** | 0 | 9 | 15 | 256 | 952 |
| **Mexico** | 0 | 103 | 99 | 320 | 421 |
| **New Zealand** | 0 | 60 | 124 | 356 | 354 |
| **Russia** | 1 | 444 | 322 | 49 | 28 |
| **South Africa** | 0 | 103 | 219 | 243 | 211 |
| **Hungary** | 0 | 146 | 92 | 214 | 298 |
| **Singapore** | 0 | 14 | 45 | 141 | 387 |
| **Egypt** | 0 | 22 | 45 | 134 | 144 |
| **Iran** | 0 | 7 | 4 | 20 | 247 |

**Number of published items for the search term:**

| **Heart** |  |  |  |  |  |
| --- | --- | --- | --- | --- | --- |
|  |  |  | **Year** |  |  |
| **Country** | **1961-1970** | **1971-1980** | **1981-1990** | **1991-2000** | **2001-2007** |
| **United States** | 260 | 38422 | 62729 | 80898 | 59654 |
| **Germany** | 741 | 11851 | 13300 | 18406 | 15456 |
| **Japan** | 24 | 7230 | 13396 | 23123 | 14504 |
| **United Kingdom** | 25 | 6349 | 11494 | 18050 | 14469 |
| **France** | 19 | 6210 | 8126 | 11073 | 7827 |
| **Italy** | 34 | 4979 | 7302 | 11186 | 9594 |
| **Canada** | 14 | 3140 | 6017 | 10010 | 8806 |
| **Netherlands** | 19 | 1413 | 3544 | 6437 | 5849 |
| **Sweden** | 29 | 2132 | 3251 | 4184 | 3536 |
| **Australia** | 5 | 1358 | 2537 | 4197 | 4593 |
| **Spain** | 8 | 908 | 1532 | 4188 | 4721 |
| **China** | 0 | 64 | 998 | 2846 | 6567 |
| **Switzerland** | 60 | 1572 | 2193 | 3234 | 3016 |
| **Israel** | 8 | 1012 | 2160 | 3524 | 3128 |
| **Russia** | 47 | 5561 | 2656 | 608 | 216 |
| **Belgium** | 15 | 1192 | 1874 | 2844 | 2389 |
| **Poland** | 10 | 1963 | 908 | 1618 | 3285 |
| **Austria** | 37 | 1159 | 1428 | 2223 | 2122 |
| **India** | 25 | 1216 | 1099 | 2162 | 2465 |
| **Brazil** | 4 | 926 | 756 | 1785 | 2871 |
| **Turkey** | 1 | 134 | 173 | 1592 | 4004 |
| **Norway** | 2 | 859 | 1312 | 1727 | 1538 |
| **Taiwan** | 1 | 178 | 416 | 2117 | 2216 |
| **Greece** | 1 | 136 | 237 | 1286 | 1667 |
| **Mexico** | 5 | 526 | 551 | 980 | 1083 |
| **Hungary** | 15 | 882 | 597 | 804 | 796 |
| **South Africa** | 6 | 637 | 1002 | 835 | 593 |
| **New Zealand** | 0 | 355 | 638 | 1026 | 1030 |
| **South Korea** | 0 | 52 | 122 | 743 | 1511 |
| **Singapore** | 0 | 82 | 164 | 381 | 552 |
| **Egypt** | 8 | 150 | 54 | 162 | 272 |
| **Iran** | 0 | 92 | 29 | 63 | 419 |

**Number of published items for the search term:**

| **Artery** |  |  |  |  |  |
| --- | --- | --- | --- | --- | --- |
|  |  |  | **Year** |  |  |
| **Country** | **1961-1970** | **1971-1980** | **1981-1990** | **1991-2000** | **2001-2007** |
| **United States** | 2 | 18298 | 32244 | 47669 | 30630 |
| **Japan** | 22 | 4293 | 8643 | 19987 | 13603 |
| **Germany** | 333 | 5040 | 6308 | 11749 | 9686 |
| **United Kingdom** | 12 | 2296 | 4806 | 10002 | 7244 |
| **France** | 11 | 3606 | 4585 | 7588 | 4841 |
| **Italy** | 23 | 1945 | 3003 | 6439 | 5962 |
| **Canada** | 6 | 1520 | 2953 | 5106 | 4186 |
| **Netherlands** | 5 | 673 | 1744 | 4007 | 3669 |
| **China** | 0 | 21 | 318 | 1776 | 5989 |
| **Sweden** | 19 | 997 | 1700 | 2613 | 1771 |
| **Australia** | 4 | 679 | 1342 | 2417 | 2352 |
| **Switzerland** | 0 | 880 | 1210 | 2536 | 2042 |
| **Spain** | 2 | 414 | 720 | 2426 | 2468 |
| **Turkey** | 0 | 65 | 118 | 1490 | 4068 |
| **Austria** | 31 | 700 | 1032 | 1649 | 1526 |
| **Israel** | 2 | 405 | 752 | 1844 | 1828 |
| **Belgium** | 8 | 610 | 923 | 1782 | 1431 |
| **Poland** | 5 | 789 | 361 | 883 | 2058 |
| **Taiwan** | 0 | 62 | 177 | 1459 | 1806 |
| **India** | 4 | 332 | 305 | 1142 | 1551 |
| **Brazil** | 1 | 343 | 258 | 862 | 1527 |
| **Russia** | 26 | 1806 | 691 | 190 | 107 |
| **South Korea** | 0 | 10 | 55 | 717 | 1768 |
| **Norway** | 0 | 390 | 563 | 856 | 661 |
| **Greece** | 1 | 76 | 132 | 886 | 1249 |
| **Hungary** | 12 | 435 | 326 | 455 | 515 |
| **Mexico** | 0 | 145 | 185 | 464 | 474 |
| **South Africa** | 2 | 242 | 390 | 350 | 246 |
| **New Zealand** | 0 | 122 | 206 | 418 | 368 |
| **Singapore** | 0 | 32 | 52 | 257 | 351 |
| **Iran** | 0 | 33 | 15 | 50 | 309 |
| **Egypt** | 0 | 53 | 19 | 90 | 171 |

**Number of published items for the search term:**

| **Vein** |  |  |  |  |  |
| --- | --- | --- | --- | --- | --- |
|  |  |  | **Year** |  |  |
| **Country** | **1961-1970** | **1971-1980** | **1981-1990** | **1991-2000** | **2001-2007** |
| **United States** | 41 | 6745 | 10906 | 16102 | 10986 |
| **Japan** | 5 | 1457 | 3012 | 7929 | 5590 |
| **Germany** | 196 | 2183 | 2561 | 4499 | 3851 |
| **United Kingdom** | 15 | 1483 | 2139 | 4162 | 3037 |
| **France** | 13 | 1550 | 2150 | 3625 | 2327 |
| **Italy** | 9 | 779 | 1266 | 2674 | 2348 |
| **Canada** | 2 | 623 | 1344 | 2279 | 1693 |
| **China** | 0 | 14 | 207 | 835 | 3727 |
| **Netherlands** | 5 | 264 | 632 | 1431 | 1079 |
| **Australia** | 10 | 285 | 689 | 1261 | 1093 |
| **Sweden** | 9 | 711 | 943 | 1033 | 605 |
| **Spain** | 0 | 197 | 382 | 1252 | 1219 |
| **Switzerland** | 18 | 469 | 595 | 1087 | 806 |
| **Austria** | 12 | 337 | 400 | 772 | 654 |
| **Turkey** | 0 | 25 | 52 | 558 | 1330 |
| **Belgium** | 6 | 310 | 428 | 700 | 520 |
| **Taiwan** | 0 | 21 | 98 | 748 | 908 |
| **India** | 3 | 175 | 210 | 575 | 778 |
| **Israel** | 0 | 211 | 351 | 599 | 564 |
| **Poland** | 4 | 304 | 133 | 364 | 659 |
| **Brazil** | 3 | 151 | 115 | 376 | 697 |
| **South Korea** | 0 | 10 | 26 | 314 | 834 |
| **Russia** | 13 | 694 | 235 | 70 | 33 |
| **Norway** | 3 | 173 | 264 | 344 | 245 |
| **Greece** | 2 | 37 | 53 | 261 | 386 |
| **Hungary** | 6 | 182 | 109 | 176 | 158 |
| **New Zealand** | 1 | 66 | 103 | 202 | 237 |
| **South Africa** | 0 | 104 | 151 | 199 | 147 |
| **Mexico** | 0 | 65 | 82 | 197 | 221 |
| **Egypt** | 3 | 42 | 42 | 79 | 119 |
| **Singapore** | 0 | 14 | 17 | 101 | 129 |
| **Iran** | 1 | 11 | 7 | 24 | 140 |

**Number of published items for the search term:**

| **Lung** |  |  |  |  |  |
| --- | --- | --- | --- | --- | --- |
|  |  |  | **Year** |  |  |
| **Country** | **1961-1970** | **1971-1980** | **1981-1990** | **1991-2000** | **2001-2007** |
| **United States** | 72 | 23819 | 38017 | 49318 | 37278 |
| **Japan** | 5 | 4390 | 8827 | 18446 | 13213 |
| **Germany** | 340 | 5867 | 6975 | 9846 | 8319 |
| **United Kingdom** | 20 | 4453 | 7261 | 10908 | 7962 |
| **France** | 9 | 4235 | 4971 | 7849 | 5762 |
| **Italy** | 10 | 3178 | 4134 | 6242 | 5628 |
| **Canada** | 11 | 2210 | 4335 | 6192 | 4819 |
| **Netherlands** | 14 | 672 | 1780 | 3791 | 3257 |
| **China** | 0 | 30 | 451 | 1834 | 5971 |
| **Spain** | 1 | 611 | 997 | 2961 | 3273 |
| **Australia** | 3 | 766 | 1440 | 2576 | 2674 |
| **Sweden** | 14 | 1118 | 1871 | 2521 | 1867 |
| **Switzerland** | 26 | 1109 | 1150 | 2274 | 1955 |
| **Russia** | 26 | 3923 | 1769 | 186 | 49 |
| **Belgium** | 3 | 889 | 1138 | 1934 | 1864 |
| **India** | 5 | 882 | 818 | 1257 | 1674 |
| **Poland** | 2 | 1206 | 529 | 915 | 1573 |
| **Austria** | 22 | 850 | 918 | 1298 | 1099 |
| **Israel** | 1 | 416 | 820 | 1371 | 1352 |
| **South Korea** | 0 | 49 | 223 | 1367 | 2235 |
| **Taiwan** | 0 | 85 | 230 | 1331 | 1823 |
| **Turkey** | 0 | 80 | 123 | 823 | 2299 |
| **New Zealand** | 0 | 137 | 1780 | 414 | 467 |
| **Brazil** | 2 | 369 | 268 | 694 | 1331 |
| **Norway** | 2 | 377 | 501 | 756 | 647 |
| **Greece** | 0 | 68 | 133 | 661 | 1163 |
| **Mexico** | 2 | 201 | 276 | 622 | 580 |
| **South Africa** | 4 | 288 | 478 | 480 | 373 |
| **Hungary** | 11 | 521 | 268 | 322 | 434 |
| **Singapore** | 0 | 75 | 99 | 241 | 358 |
| **Iran** | 1 | 57 | 18 | 54 | 259 |
| **Egypt** | 4 | 65 | 33 | 122 | 152 |

**Number of published items for the search term:**

| **Stomach** |  |  |  |  |  |
| --- | --- | --- | --- | --- | --- |
|  |  |  | **Year** |  |  |
| **Country** | **1961-1970** | **1971-1980** | **1981-1990** | **1991-2000** | **2001-2007** |
| **United States** | 153 | 6336 | 8450 | 9083 | 5729 |
| **Japan** | 5 | 3657 | 5921 | 9180 | 5460 |
| **Germany** | 108 | 3155 | 2826 | 2780 | 1801 |
| **United Kingdom** | 14 | 2117 | 2871 | 2869 | 1514 |
| **Italy** | 6 | 1524 | 2320 | 2337 | 1322 |
| **France** | 3 | 1686 | 1751 | 1718 | 1028 |
| **China** | 0 | 18 | 333 | 958 | 2352 |
| **Canada** | 5 | 684 | 1025 | 1218 | 687 |
| **Sweden** | 7 | 645 | 866 | 894 | 574 |
| **Spain** | 0 | 438 | 538 | 971 | 719 |
| **Russia** | 24 | 1745 | 636 | 108 | 21 |
| **Australia** | 6 | 379 | 547 | 895 | 614 |
| **Netherlands** | 4 | 239 | 570 | 833 | 554 |
| **Poland** | 4 | 642 | 364 | 513 | 625 |
| **India** | 5 | 362 | 311 | 499 | 577 |
| **Switzerland** | 7 | 453 | 458 | 493 | 326 |
| **Belgium** | 3 | 360 | 358 | 466 | 352 |
| **South Korea** | 0 | 33 | 75 | 527 | 903 |
| **Norway** | 0 | 325 | 350 | 374 | 279 |
| **Taiwan** | 0 | 29 | 116 | 655 | 513 |
| **Brazil** | 2 | 237 | 125 | 315 | 543 |
| **Austria** | 6 | 312 | 285 | 299 | 250 |
| **Turkey** | 0 | 38 | 60 | 406 | 636 |
| **Israel** | 3 | 143 | 263 | 271 | 213 |
| **Hungary** | 2 | 288 | 207 | 238 | 150 |
| **South Africa** | 1 | 196 | 216 | 178 | 124 |
| **Greece** | 0 | 38 | 87 | 221 | 265 |
| **Mexico** | 0 | 51 | 81 | 163 | 205 |
| **New Zealand** | 1 | 83 | 125 | 139 | 123 |
| **Singapore** | 0 | 49 | 41 | 74 | 105 |
| **Egypt** | 4 | 37 | 31 | 59 | 65 |
| **Iran** | 0 | 34 | 5 | 33 | 101 |

**Number of published items for the search term:**

| **Intestine** |  |  |  |  |  |
| --- | --- | --- | --- | --- | --- |
|  |  |  | **Year** |  |  |
| **Country** | **1961-1970** | **1971-1980** | **1981-1990** | **1991-2000** | **2001-2007** |
| **United States** | 111 | 8230 | 10344 | 13109 | 8024 |
| **Japan** | 13 | 2216 | 2666 | 5234 | 3724 |
| **United Kingdom** | 33 | 2628 | 3479 | 3777 | 2083 |
| **Germany** | 207 | 2503 | 2076 | 2719 | 2029 |
| **France** | 3 | 1897 | 1927 | 2478 | 1584 |
| **Canada** | 6 | 840 | 1332 | 1916 | 1171 |
| **Italy** | 20 | 1070 | 1310 | 1546 | 1201 |
| **Sweden** | 10 | 722 | 1123 | 1253 | 696 |
| **Australia** | 9 | 481 | 674 | 1161 | 743 |
| **Netherlands** | 3 | 294 | 651 | 1081 | 835 |
| **Spain** | 2 | 332 | 511 | 958 | 826 |
| **India** | 12 | 562 | 515 | 560 | 703 |
| **Russia** | 31 | 1404 | 562 | 98 | 62 |
| **Switzerland** | 12 | 516 | 430 | 645 | 513 |
| **China** | 0 | 11 | 73 | 323 | 1426 |
| **Belgium** | 6 | 302 | 332 | 526 | 422 |
| **Israel** | 1 | 264 | 309 | 558 | 352 |
| **Poland** | 1 | 567 | 196 | 243 | 464 |
| **Brazil** | 2 | 234 | 90 | 336 | 532 |
| **Austria** | 15 | 276 | 253 | 302 | 248 |
| **Norway** | 0 | 214 | 249 | 303 | 284 |
| **Turkey** | 0 | 34 | 35 | 359 | 563 |
| **Taiwan** | 0 | 24 | 76 | 356 | 363 |
| **South Korea** | 1 | 16 | 25 | 237 | 499 |
| **Hungary** | 10 | 270 | 146 | 159 | 135 |
| **Mexico** | 0 | 90 | 79 | 193 | 197 |
| **Greece** | 2 | 58 | 61 | 139 | 258 |
| **South Africa** | 0 | 113 | 137 | 156 | 73 |
| **New Zealand** | 1 | 73 | 75 | 174 | 149 |
| **Egypt** | 2 | 81 | 22 | 86 | 93 |
| **Singapore** | 1 | 33 | 22 | 54 | 90 |
| **Iran** | 0 | 52 | 8 | 29 | 67 |

**Number of published items for the search term:**

| **Liver** |  |  |  |  |  |
| --- | --- | --- | --- | --- | --- |
|  |  |  | **Year** |  |  |
| **Country** | **1961-1970** | **1971-1980** | **1981-1990** | **1991-2000** | **2001-2007** |
| **United States** | 701 | 30516 | 40851 | 49647 | 33038 |
| **Japan** | 44 | 8129 | 15660 | 27427 | 17004 |
| **Germany** | 836 | 8123 | 7973 | 11524 | 9066 |
| **United Kingdom** | 62 | 6384 | 7956 | 9815 | 6495 |
| **France** | 25 | 4978 | 6438 | 9724 | 6215 |
| **Italy** | 70 | 4047 | 5662 | 8182 | 6384 |
| **Canada** | 57 | 2975 | 4220 | 5930 | 4102 |
| **China** | 0 | 38 | 567 | 2783 | 9585 |
| **Spain** | 2 | 1004 | 2098 | 5028 | 4449 |
| **India** | 31 | 1923 | 1873 | 2975 | 3418 |
| **Netherlands** | 18 | 1128 | 2417 | 3633 | 2641 |
| **Sweden** | 49 | 1800 | 2751 | 2961 | 1741 |
| **Australia** | 27 | 1190 | 2037 | 3260 | 2385 |
| **Switzerland** | 67 | 1168 | 1476 | 2475 | 1874 |
| **Russia** | 65 | 4461 | 1846 | 277 | 119 |
| **Belgium** | 20 | 949 | 1370 | 2283 | 1692 |
| **Poland** | 13 | 1647 | 815 | 1303 | 1863 |
| **Taiwan** | 1 | 127 | 443 | 2319 | 2610 |
| **Austria** | 34 | 658 | 2037 | 1195 | 1117 |
| **Israel** | 11 | 674 | 915 | 1488 | 1385 |
| **South Korea** | 1 | 86 | 190 | 1444 | 2648 |
| **Turkey** | 1 | 117 | 122 | 1243 | 2675 |
| **Brazil** | 10 | 401 | 297 | 1112 | 1922 |
| **Norway** | 6 | 653 | 968 | 1120 | 765 |
| **Hungary** | 19 | 764 | 523 | 611 | 536 |
| **Mexico** | 5 | 316 | 339 | 787 | 729 |
| **Greece** | 5 | 147 | 208 | 688 | 1109 |
| **South Africa** | 2 | 469 | 522 | 496 | 341 |
| **Egypt** | 12 | 287 | 191 | 504 | 585 |
| **New Zealand** | 2 | 228 | 291 | 433 | 461 |
| **Singapore** | 0 | 74 | 122 | 363 | 540 |
| **Iran** | 0 | 64 | 17 | 78 | 390 |

**Number of published items for the search term:**

| **Pancreas** |  |  |  |  |  |
| --- | --- | --- | --- | --- | --- |
|  |  |  | **Year** |  |  |
| **Country** | **1961-1970** | **1971-1980** | **1981-1990** | **1991-2000** | **2001-2007** |
| **United States** | 89 | 5278 | 7926 | 9849 | 6728 |
| **Japan** | 10 | 1710 | 2973 | 4989 | 3278 |
| **Germany** | 104 | 2066 | 2464 | 2943 | 2127 |
| **France** | 15 | 1394 | 1675 | 1949 | 1131 |
| **United Kingdom** | 14 | 1108 | 1731 | 1838 | 1222 |
| **Italy** | 7 | 734 | 1143 | 1517 | 1154 |
| **Sweden** | 25 | 813 | 1061 | 1286 | 655 |
| **Canada** | 5 | 446 | 756 | 1146 | 751 |
| **Belgium** | 7 | 428 | 544 | 761 | 416 |
| **Switzerland** | 17 | 434 | 456 | 756 | 482 |
| **Spain** | 3 | 232 | 349 | 791 | 613 |
| **Netherlands** | 7 | 204 | 437 | 657 | 361 |
| **Australia** | 5 | 170 | 404 | 513 | 350 |
| **China** | 0 | 6 | 74 | 269 | 1077 |
| **Poland** | 2 | 252 | 163 | 305 | 428 |
| **Russia** | 15 | 736 | 237 | 37 | 9 |
| **India** | 2 | 187 | 144 | 216 | 377 |
| **Israel** | 1 | 142 | 167 | 324 | 237 |
| **Austria** | 5 | 167 | 200 | 212 | 187 |
| **South Korea** | 0 | 24 | 27 | 171 | 350 |
| **Hungary** | 3 | 144 | 120 | 162 | 142 |
| **Brazil** | 3 | 92 | 56 | 148 | 247 |
| **Norway** | 0 | 117 | 179 | 136 | 112 |
| **Turkey** | 0 | 25 | 20 | 134 | 319 |
| **Taiwan** | 0 | 12 | 37 | 217 | 226 |
| **South Africa** | 0 | 110 | 120 | 111 | 61 |
| **Greece** | 0 | 20 | 33 | 145 | 178 |
| **Mexico** | 0 | 38 | 46 | 97 | 106 |
| **New Zealand** | 0 | 33 | 51 | 62 | 58 |
| **Singapore** | 0 | 9 | 13 | 46 | 83 |
| **Egypt** | 0 | 23 | 7 | 29 | 21 |
| **Iran** | 0 | 5 | 1 | 6 | 44 |

**Number of published items for the search term:**

| **Kidney** |  |  |  |  |  |
| --- | --- | --- | --- | --- | --- |
|  |  |  | **Year** |  |  |
| **Country** | **1961-1970** | **1971-1980** | **1981-1990** | **1991-2000** | **2001-2007** |
| **United States** | 247 | 23928 | 33231 | 38779 | 26280 |
| **Japan** | 28 | 4374 | 8031 | 13099 | 8121 |
| **Germany** | 662 | 6749 | 7030 | 9517 | 6960 |
| **United Kingdom** | 39 | 4132 | 6150 | 7284 | 4984 |
| **France** | 18 | 3692 | 4787 | 6537 | 4163 |
| **Italy** | 21 | 2762 | 3485 | 4974 | 4126 |
| **Canada** | 15 | 1860 | 3015 | 4263 | 3443 |
| **Spain** | 0 | 768 | 1495 | 3592 | 2736 |
| **Netherlands** | 17 | 730 | 1976 | 2899 | 2190 |
| **Australia** | 8 | 1090 | 1731 | 2368 | 2070 |
| **Sweden** | 32 | 1370 | 2035 | 2268 | 1399 |
| **Switzerland** | 63 | 1191 | 1430 | 1997 | 1510 |
| **India** | 11 | 822 | 839 | 1553 | 1971 |
| **Belgium** | 19 | 866 | 1066 | 1496 | 1272 |
| **China** | 0 | 8 | 228 | 944 | 3515 |
| **Poland** | 8 | 1101 | 549 | 972 | 1590 |
| **Israel** | 2 | 619 | 1023 | 1406 | 1105 |
| **Turkey** | 1 | 84 | 116 | 1194 | 2322 |
| **Austria** | 11 | 729 | 807 | 1088 | 988 |
| **Russia** | 49 | 2393 | 716 | 125 | 58 |
| **South Korea** | 0 | 45 | 877 | 877 | 1394 |
| **Brazil** | 6 | 315 | 289 | 837 | 1489 |
| **Taiwan** | 0 | 88 | 180 | 981 | 1176 |
| **Hungary** | 31 | 728 | 343 | 444 | 347 |
| **Norway** | 2 | 466 | 120 | 700 | 573 |
| **Greece** | 1 | 136 | 173 | 481 | 605 |
| **Mexico** | 1 | 230 | 197 | 419 | 499 |
| **New Zealand** | 0 | 260 | 285 | 416 | 330 |
| **South Africa** | 3 | 287 | 308 | 339 | 228 |
| **Egypt** | 8 | 109 | 72 | 275 | 369 |
| **Iran** | 2 | 34 | 14 | 106 | 462 |
| **Singapore** | 0 | 58 | 64 | 211 | 231 |

**Number of published items for the search term:**

| **Genital** |  |  |  |  |  |
| --- | --- | --- | --- | --- | --- |
|  |  |  | **Year** |  |  |
| **Country** | **1961-1970** | **1971-1980** | **1981-1990** | **1991-2000** | **2001-2007** |
| **United States** | 10 | 1360 | 2585 | 3933 | 2955 |
| **United Kingdom** | 1 | 375 | 757 | 1180 | 1014 |
| **France** | 7 | 464 | 603 | 938 | 778 |
| **Germany** | 41 | 489 | 506 | 954 | 786 |
| **Japan** | 1 | 195 | 410 | 746 | 553 |
| **Italy** | 2 | 187 | 333 | 697 | 554 |
| **Canada** | 1 | 116 | 284 | 417 | 385 |
| **Sweden** | 6 | 111 | 187 | 370 | 267 |
| **Australia** | 1 | 81 | 150 | 384 | 301 |
| **India** | 1 | 127 | 136 | 248 | 304 |
| **Spain** | 0 | 76 | 59 | 311 | 346 |
| **Netherlands** | 2 | 29 | 118 | 261 | 237 |
| **Switzerland** | 7 | 80 | 97 | 170 | 157 |
| **Turkey** | 0 | 8 | 17 | 154 | 332 |
| **Belgium** | 0 | 51 | 95 | 183 | 153 |
| **Brazil** | 2 | 41 | 30 | 136 | 250 |
| **Israel** | 0 | 51 | 93 | 169 | 126 |
| **Mexico** | 0 | 55 | 33 | 145 | 158 |
| **Poland** | 2 | 102 | 44 | 89 | 146 |
| **Austria** | 1 | 70 | 66 | 113 | 87 |
| **China** | 0 | 0 | 10 | 84 | 228 |
| **Norway** | 0 | 32 | 39 | 91 | 87 |
| **South Africa** | 0 | 26 | 54 | 71 | 75 |
| **Russia** | 0 | 136 | 64 | 7 | 9 |
| **Greece** | 0 | 14 | 13 | 63 | 107 |
| **Taiwan** | 0 | 3 | 6 | 87 | 99 |
| **South Korea** | 0 | 3 | 10 | 39 | 126 |
| **Hungary** | 0 | 31 | 30 | 48 | 42 |
| **New Zealand** | 0 | 11 | 30 | 56 | 51 |
| **Egypt** | 0 | 18 | 21 | 45 | 63 |
| **Singapore** | 0 | 4 | 28 | 51 | 31 |
| **Iran** | 0 | 5 | 2 | 6 | 51 |

**Number of published items for the search term:**

| **Hormone** |  |  |  |  |  |
| --- | --- | --- | --- | --- | --- |
|  |  |  | **Year** |  |  |
| **Country** | **1961-1970** | **1971-1980** | **1981-1990** | **1991-2000** | **2001-2007** |
| **United States** | 342 | 23849 | 36089 | 49859 | 35004 |
| **United Kingdom** | 68 | 5642 | 7741 | 11214 | 8153 |
| **Japan** | 25 | 3576 | 6677 | 12011 | 8227 |
| **Germany** | 335 | 4984 | 5724 | 9132 | 7359 |
| **France** | 45 | 4228 | 6186 | 9391 | 5707 |
| **Italy** | 42 | 2425 | 4314 | 7458 | 5835 |
| **Canada** | 30 | 2457 | 3794 | 6057 | 4811 |
| **Sweden** | 34 | 1286 | 2492 | 3769 | 2646 |
| **Netherlands** | 22 | 932 | 2038 | 3859 | 3038 |
| **Australia** | 11 | 1083 | 1948 | 3577 | 3044 |
| **Spain** | 2 | 468 | 1164 | 3698 | 3310 |
| **Switzerland** | 41 | 1074 | 1349 | 2410 | 1729 |
| **Belgium** | 15 | 1028 | 1350 | 2321 | 1811 |
| **Israel** | 7 | 818 | 1387 | 2323 | 1749 |
| **India** | 18 | 781 | 859 | 1460 | 1610 |
| **Poland** | 8 | 912 | 614 | 1089 | 1760 |
| **China** | 0 | 6 | 183 | 896 | 2890 |
| **Austria** | 15 | 447 | 654 | 1108 | 1027 |
| **Russia** | 26 | 1744 | 1054 | 215 | 144 |
| **Brazil** | 2 | 181 | 292 | 926 | 1598 |
| **Hungary** | 9 | 595 | 683 | 826 | 657 |
| **Turkey** | 1 | 28 | 73 | 782 | 1870 |
| **Mexico** | 10 | 346 | 322 | 856 | 817 |
| **Norway** | 5 | 351 | 520 | 669 | 695 |
| **New Zealand** | 2 | 209 | 447 | 793 | 643 |
| **Greece** | 0 | 66 | 180 | 734 | 1024 |
| **Taiwan** | 0 | 42 | 159 | 775 | 946 |
| **South Korea** | 0 | 19 | 39 | 529 | 1039 |
| **South Africa** | 1 | 256 | 332 | 418 | 304 |
| **Egypt** | 6 | 99 | 102 | 174 | 264 |
| **Singapore** | 1 | 42 | 90 | 214 | 251 |
| **Iran** | 0 | 22 | 10 | 36 | 322 |

**Number of published items for the search term:**

| **Arm** |  |  |  |  |  |
| --- | --- | --- | --- | --- | --- |
|  |  |  | **Year** |  |  |
| **Country** | **1961-1970** | **1971-1980** | **1981-1990** | **1991-2000** | **2001-2007** |
| **United States** | 64 | 2310 | 5570 | 14331 | 12565 |
| **United Kingdom** | 34 | 493 | 1228 | 3849 | 4012 |
| **Germany** | 94 | 705 | 989 | 3064 | 3464 |
| **Japan** | 8 | 381 | 780 | 3622 | 3443 |
| **France** | 8 | 368 | 742 | 2391 | 2402 |
| **Canada** | 7 | 317 | 792 | 2170 | 2408 |
| **Italy** | 4 | 256 | 545 | 2014 | 2176 |
| **Netherlands** | 13 | 142 | 360 | 1302 | 1524 |
| **China** | 0 | 3 | 121 | 641 | 2432 |
| **Australia** | 9 | 118 | 372 | 1102 | 1453 |
| **Sweden** | 3 | 197 | 376 | 991 | 794 |
| **Spain** | 0 | 67 | 143 | 865 | 1129 |
| **Switzerland** | 11 | 145 | 232 | 664 | 841 |
| **Belgium** | 2 | 113 | 157 | 586 | 694 |
| **India** | 8 | 91 | 170 | 412 | 815 |
| **Brazil** | 0 | 40 | 55 | 421 | 753 |
| **South Korea** | 0 | 4 | 26 | 384 | 854 |
| **Israel** | 0 | 78 | 142 | 451 | 492 |
| **Taiwan** | 0 | 6 | 43 | 409 | 654 |
| **Austria** | 7 | 99 | 99 | 358 | 496 |
| **Turkey** | 0 | 5 | 10 | 268 | 645 |
| **Poland** | 3 | 77 | 49 | 236 | 490 |
| **Greece** | 0 | 22 | 38 | 251 | 404 |
| **Mexico** | 1 | 34 | 46 | 273 | 348 |
| **Norway** | 0 | 42 | 55 | 238 | 249 |
| **New Zealand** | 0 | 22 | 61 | 170 | 248 |
| **Singapore** | 0 | 8 | 25 | 139 | 261 |
| **South Africa** | 0 | 19 | 66 | 130 | 188 |
| **Hungary** | 2 | 25 | 56 | 118 | 185 |
| **Russia** | 3 | 162 | 69 | 31 | 22 |
| **Iran** | 0 | 6 | 3 | 38 | 192 |
| **Egypt** | 1 | 1 | 15 | 65 | 82 |

**Number of published items for the search term:**

| **Feet** |  |  |  |  |  |
| --- | --- | --- | --- | --- | --- |
|  |  |  | **Year** |  |  |
| **Country** | **1961-1970** | **1971-1980** | **1981-1990** | **1991-2000** | **2001-2007** |
| **United States** | 158 | 2311 | 4361 | 8777 | 7085 |
| **United Kingdom** | 24 | 624 | 991 | 1898 | 2486 |
| **Germany** | 83 | 739 | 708 | 1307 | 1626 |
| **Japan** | 9 | 359 | 655 | 1569 | 1528 |
| **France** | 9 | 457 | 506 | 988 | 1095 |
| **Italy** | 6 | 222 | 423 | 980 | 927 |
| **Canada** | 15 | 215 | 437 | 846 | 929 |
| **Australia** | 6 | 120 | 218 | 498 | 786 |
| **Netherlands** | 7 | 87 | 190 | 515 | 696 |
| **India** | 7 | 196 | 245 | 394 | 568 |
| **Spain** | 0 | 53 | 126 | 497 | 526 |
| **China** | 0 | 4 | 57 | 195 | 941 |
| **Switzerland** | 5 | 202 | 169 | 360 | 407 |
| **Sweden** | 7 | 117 | 252 | 396 | 331 |
| **Israel** | 1 | 81 | 152 | 276 | 295 |
| **Belgium** | 0 | 78 | 84 | 240 | 348 |
| **Brazil** | 6 | 55 | 50 | 262 | 370 |
| **Turkey** | 0 | 19 | 13 | 167 | 539 |
| **Austria** | 6 | 88 | 84 | 207 | 280 |
| **Taiwan** | 0 | 25 | 25 | 266 | 324 |
| **Poland** | 0 | 154 | 63 | 130 | 255 |
| **South Korea** | 0 | 14 | 15 | 124 | 377 |
| **South Africa** | 3 | 43 | 61 | 96 | 119 |
| **Mexico** | 2 | 23 | 41 | 133 | 114 |
| **Norway** | 1 | 38 | 57 | 101 | 105 |
| **New Zealand** | 0 | 12 | 42 | 75 | 140 |
| **Greece** | 0 | 13 | 22 | 65 | 145 |
| **Russia** | 4 | 158 | 37 | 22 | 14 |
| **Hungary** | 1 | 67 | 43 | 53 | 49 |
| **Singapore** | 0 | 15 | 14 | 56 | 95 |
| **Egypt** | 1 | 12 | 7 | 31 | 53 |
| **Iran** | 0 | 12 | 1 | 11 | 71 |

**Number of published items for the search term:**

| **Muscle** |  |  |  |  |  |
| --- | --- | --- | --- | --- | --- |
|  |  |  | **Year** |  |  |
| **Country** | **1961-1970** | **1971-1980** | **1981-1990** | **1991-2000** | **2001-2007** |
| **United States** | 521 | 28342 | 45165 | 67291 | 44671 |
| **Japan** | 60 | 6821 | 11893 | 22254 | 14860 |
| **United Kingdom** | 93 | 6962 | 9299 | 14575 | 10260 |
| **Germany** | 901 | 7473 | 7883 | 12925 | 10093 |
| **Canada** | 37 | 3206 | 6155 | 10347 | 7569 |
| **France** | 32 | 3608 | 5143 | 9321 | 7219 |
| **Italy** | 64 | 2629 | 4018 | 7633 | 6517 |
| **Australia** | 31 | 1362 | 2555 | 4922 | 4813 |
| **Sweden** | 69 | 2168 | 3705 | 4669 | 2942 |
| **Netherlands** | 23 | 1011 | 2402 | 4731 | 3770 |
| **China** | 0 | 25 | 630 | 2212 | 6295 |
| **Spain** | 7 | 508 | 965 | 3628 | 3480 |
| **Switzerland** | 69 | 1056 | 1591 | 2841 | 2488 |
| **Belgium** | 19 | 1023 | 1322 | 2351 | 2351 |
| **Russia** | 85 | 4149 | 1708 | 389 | 148 |
| **Israel** | 15 | 680 | 1236 | 2174 | 1740 |
| **Brazil** | 5 | 478 | 463 | 1558 | 2590 |
| **India** | 35 | 1028 | 815 | 1342 | 1663 |
| **Poland** | 17 | 1312 | 635 | 1119 | 1771 |
| **Austria** | 28 | 551 | 782 | 1729 | 1495 |
| **Turkey** | 1 | 87 | 129 | 1154 | 2660 |
| **Taiwan** | 1 | 104 | 295 | 1722 | 1740 |
| **Norway** | 13 | 547 | 714 | 1152 | 1044 |
| **South Korea** | 0 | 41 | 120 | 984 | 2165 |
| **Hungary** | 19 | 917 | 588 | 823 | 683 |
| **Mexico** | 4 | 229 | 312 | 770 | 781 |
| **New Zealand** | 1 | 231 | 386 | 617 | 752 |
| **Greece** | 2 | 93 | 153 | 589 | 1050 |
| **South Africa** | 2 | 318 | 450 | 552 | 369 |
| **Singapore** | 1 | 61 | 104 | 273 | 491 |
| **Egypt** | 11 | 143 | 84 | 285 | 357 |
| **Iran** | 0 | 54 | 21 | 91 | 339 |
