# Supplementary material for: Institutional operating figures in basic and applied sciences: Scientometric analysis of quantitative output benchmarking
Source: Health Res Policy Syst. 2008 Jun 13;6:6. doi: 10.1186/1478-4505-6-6 (PMC2459159; doi:10.1186/1478-4505-6-6)
Supplement: Supplementary file 2 [file 1478-4505-6-6-S2.doc]

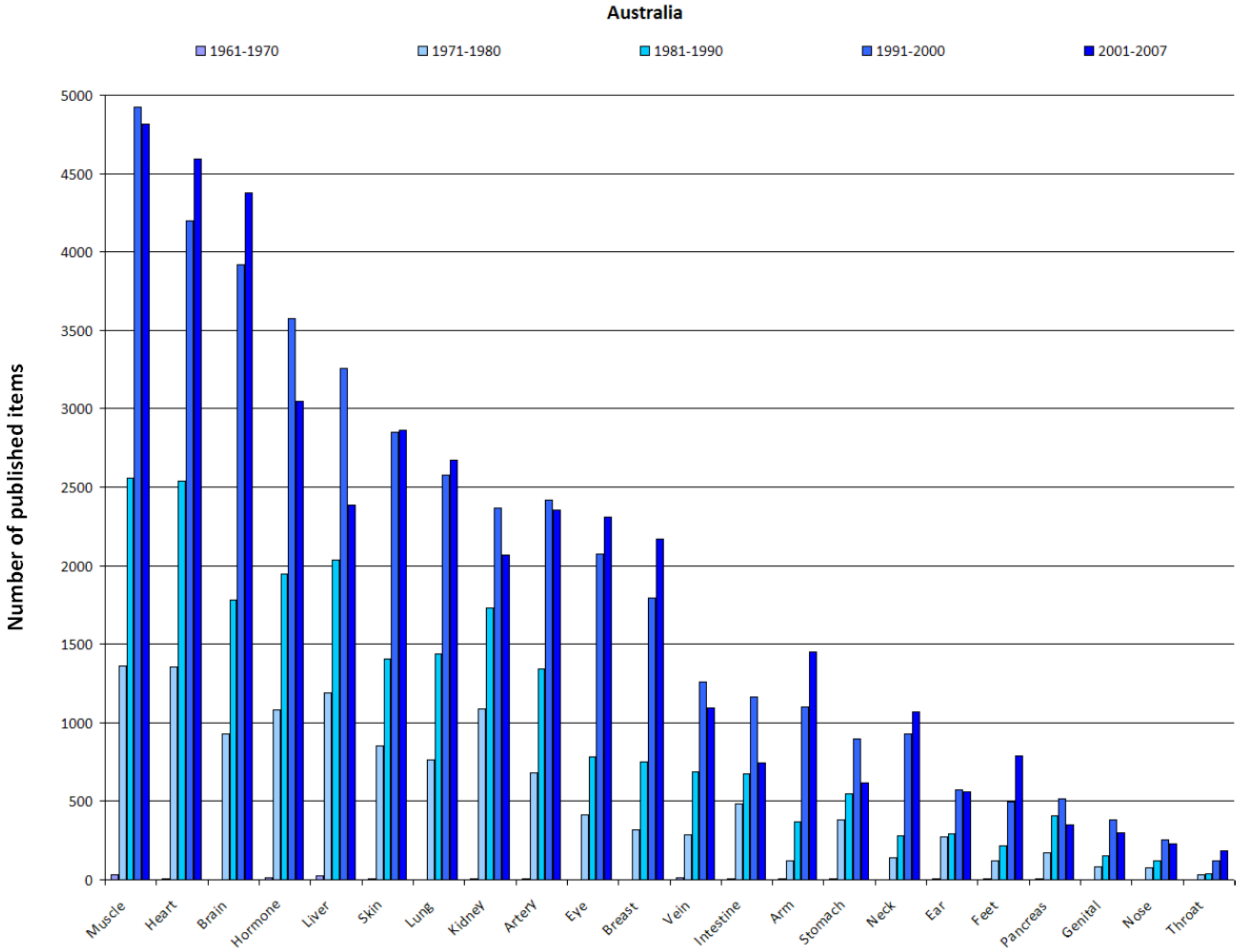

Australia
1961-1970
1971-1980
1981-1990
1991-2000
2001-2007
Number of published items
5000
4500
4000
3500
3000
2500
2000
1500
1000
500
0
Muscle
Heart
Brain
Hormone
Liver
Skin
Lung
Kidney
Artery
Eye
Breast
Vein
Intestine
Arm
Stomach
Neck
Ear
Feet
Pancreas
Genital
Nose
Throat

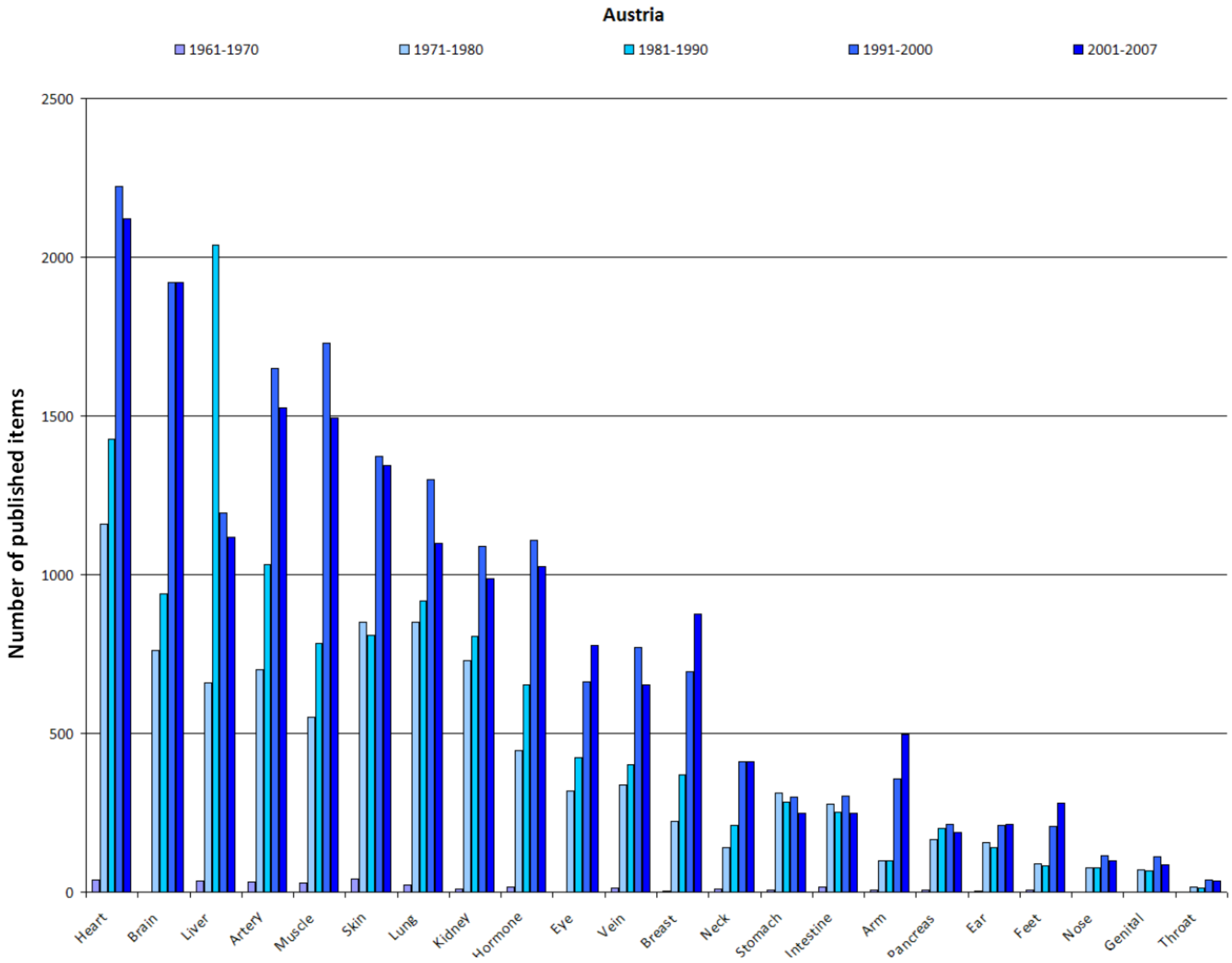

Austria
1961-1970
1971-1980
1981-1990
1991-2000
2001-2007
Number of published items
2500
2000
1500
1000
500
0
Heart
Brain
Liver
Artery
Muscle
Skin
Lung
Kidney
Hormone
Eye
Vein
Breast
Neck
Stomach
Intestine
Arm
Pancreas
Ear
Feet
Nose
Genital
Throat

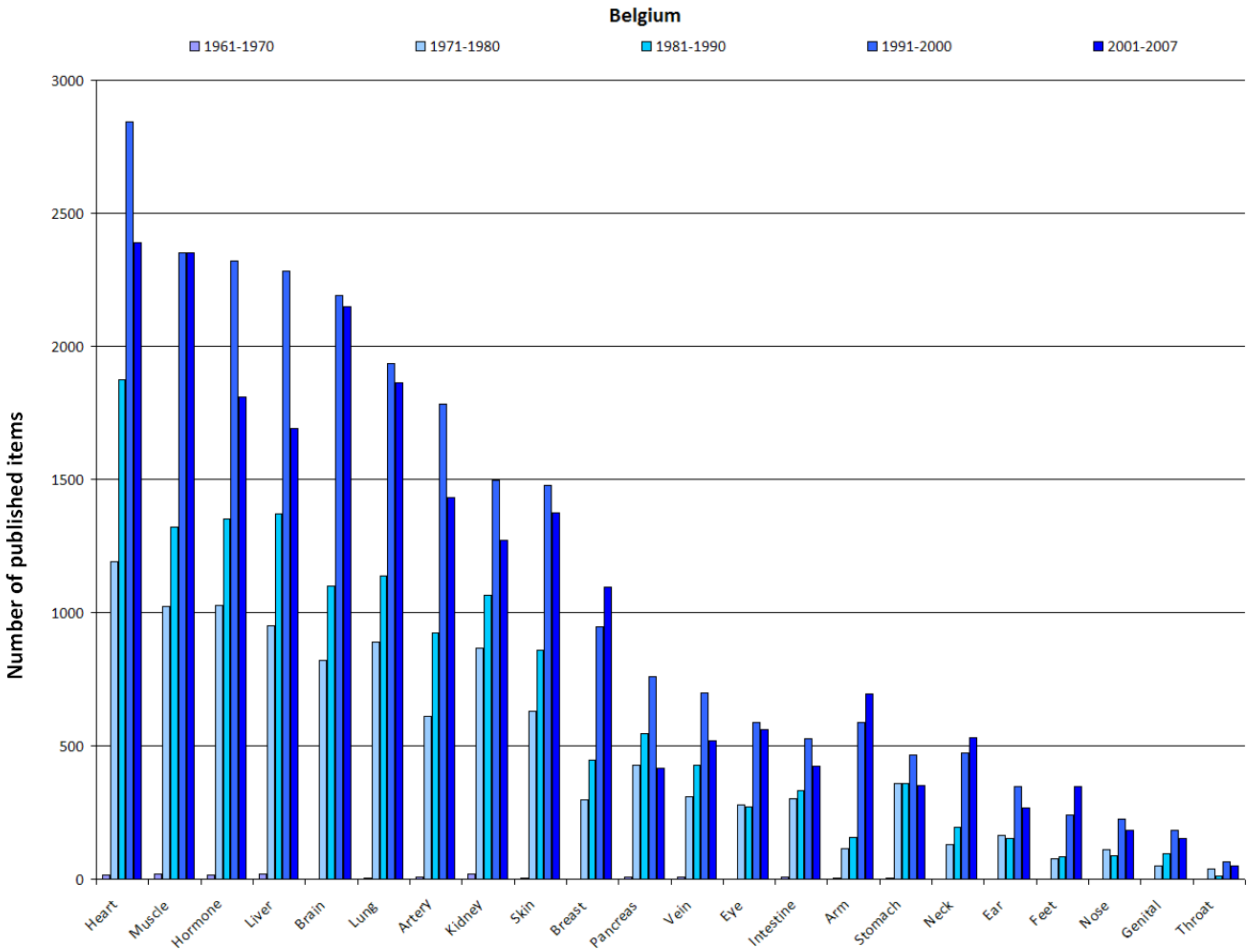
Belgium
1961-1970
1971-1980
1981-1990
1991-2000
2001-2007
Number of published items
3000
2500
2000
1500
1000
500
0
Heart
Muscle
Hormone
Liver
Brain
Lung
Artery
Kidney
Skin
Breast
Pancreas
Vein
Eye
Intestine
Arm
Stomach
Neck
Ear
Feet
Nose
Genital
Throat

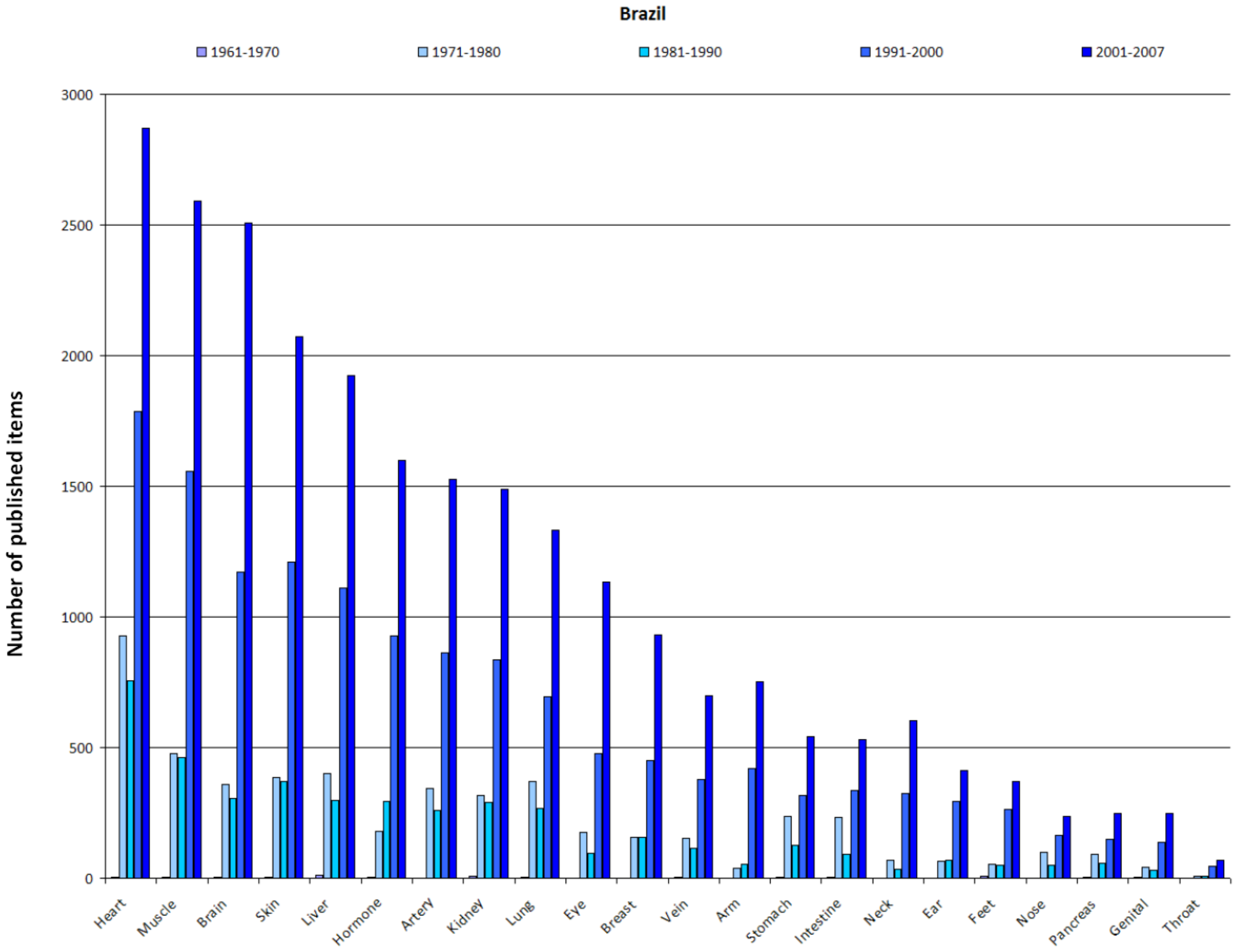
Brazil
1961-1970
1971-1980
1981-1990
1991-2000
2001-2007
Number of published items
0
500
1000
1500
2000
2500
3000
Heart
Muscle
Brain
Skin
Liver
Hormone
Artery
Kidney
Lung
Eye
Breast
Vein
Arm
Stomach
Intestine
Neck
Ear
Feet
Nose
Pancreas
Genital
Throat

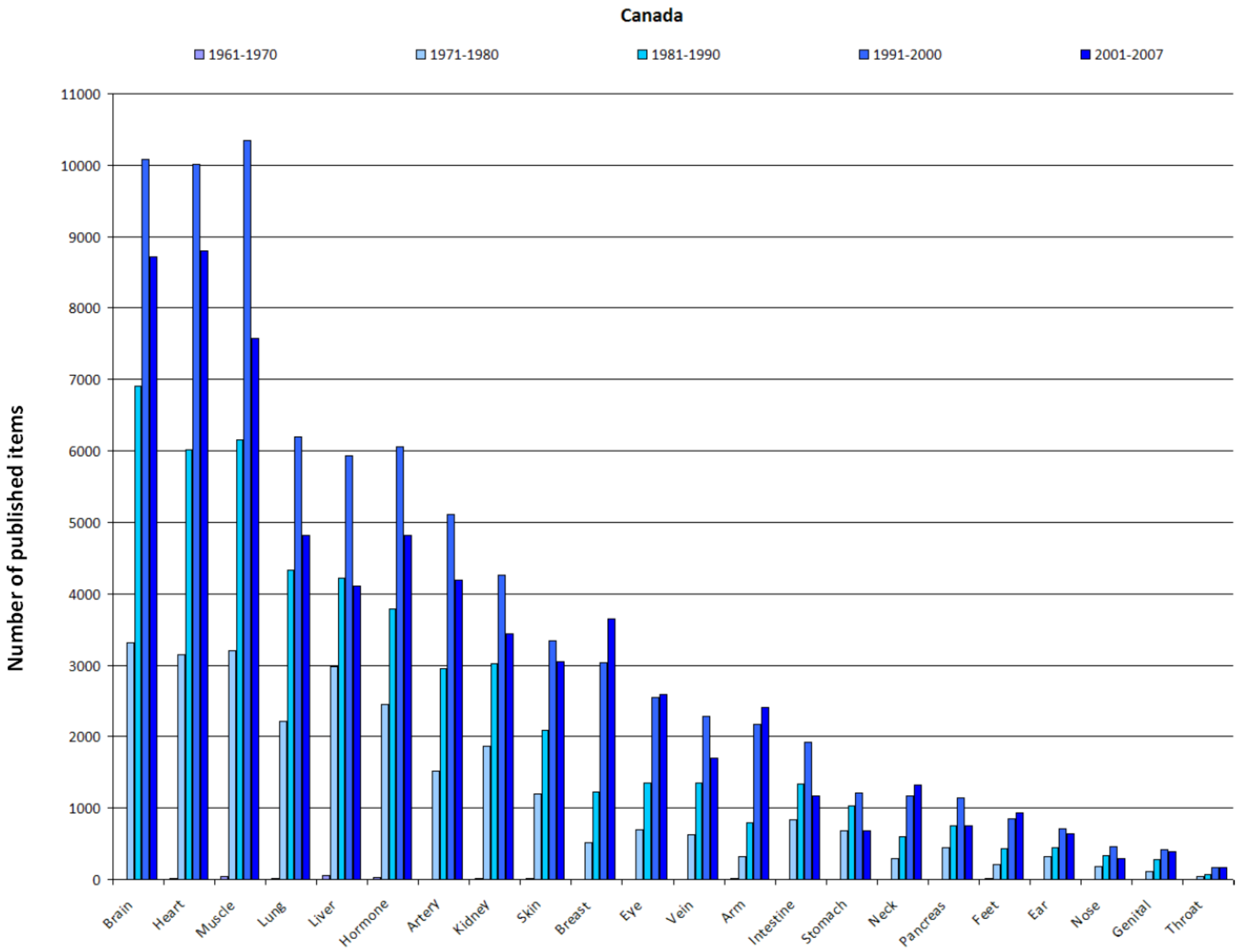
Canada
1961-1970
1971-1980
1981-1990
1991-2000
2001-2007
Number of published items
0
1000
2000
3000
4000
5000
6000
7000
8000
9000
10000
11000
Brain
Heart
Muscle
Lung
Liver
Hormone
Artery
Kidney
Skin
Breast
Eye
Vein
Arm
Intestine
Stomach
Neck
Pancreas
Feet
Ear
Nose
Genital
Throat

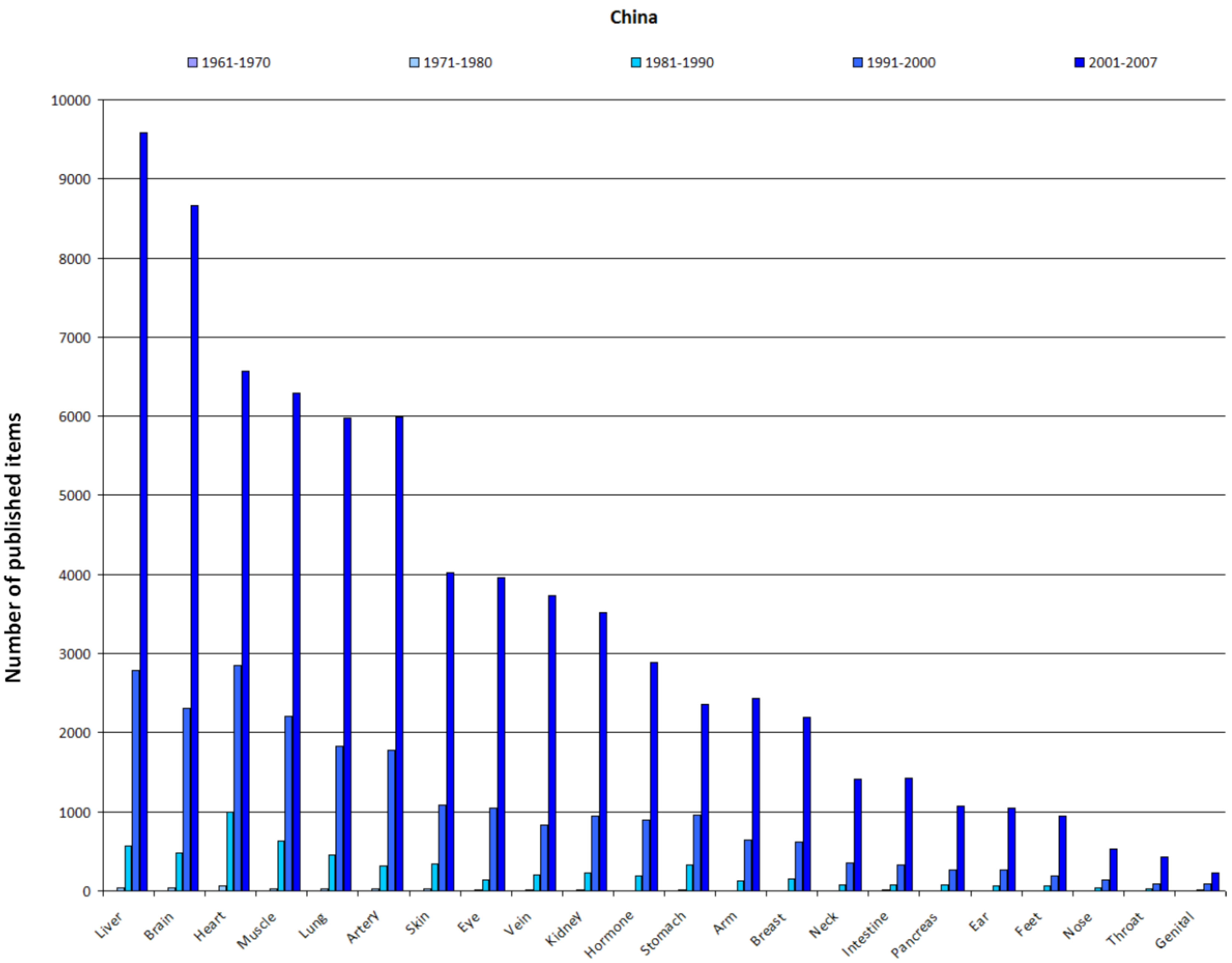
China
1961-1970
1971-1980
1981-1990
1991-2000
2001-2007
Number of published items
0
1000
2000
3000
4000
5000
6000
7000
8000
9000
10000
Liver
Brain
Heart
Muscle
Lung
Artery
Skin
Eye
Vein
Kidney
Hormone
Stomach
Arm
Breast
Neck
Intestine
Pancreas
Ear
Feet
Nose
Throat
Genital

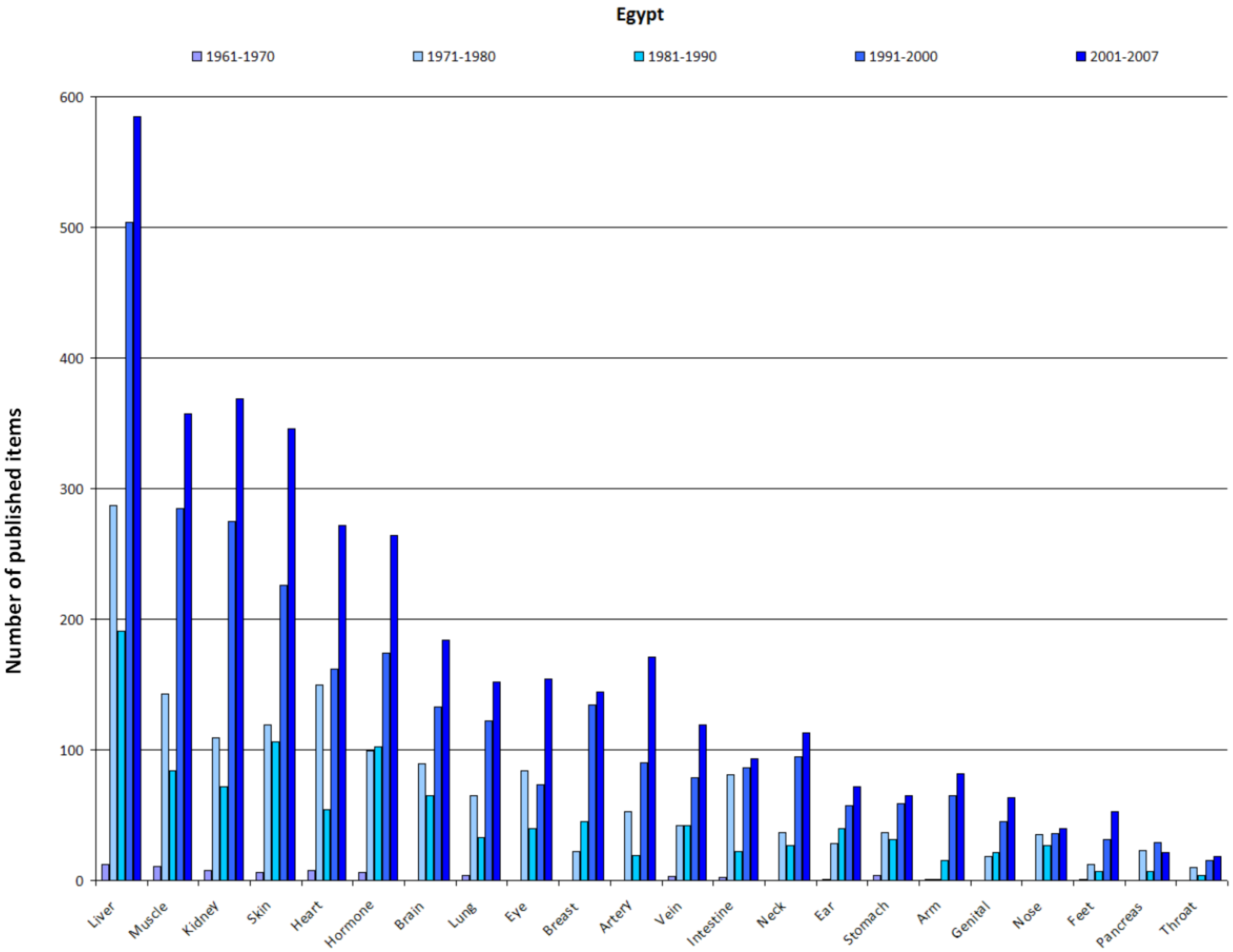

Egypt
1961-1970
1971-1980
1981-1990
1991-2000
2001-2007
Number of published items
600
500
400
300
200
100
0
Liver
Muscle
Kidney
Skin
Heart
Hormone
Brain
Lung
Eye
Breast
Artery
Vein
Intestine
Neck
Ear
Stomach
Arm
Genital
Nose
Feet
Pancreas
Throat

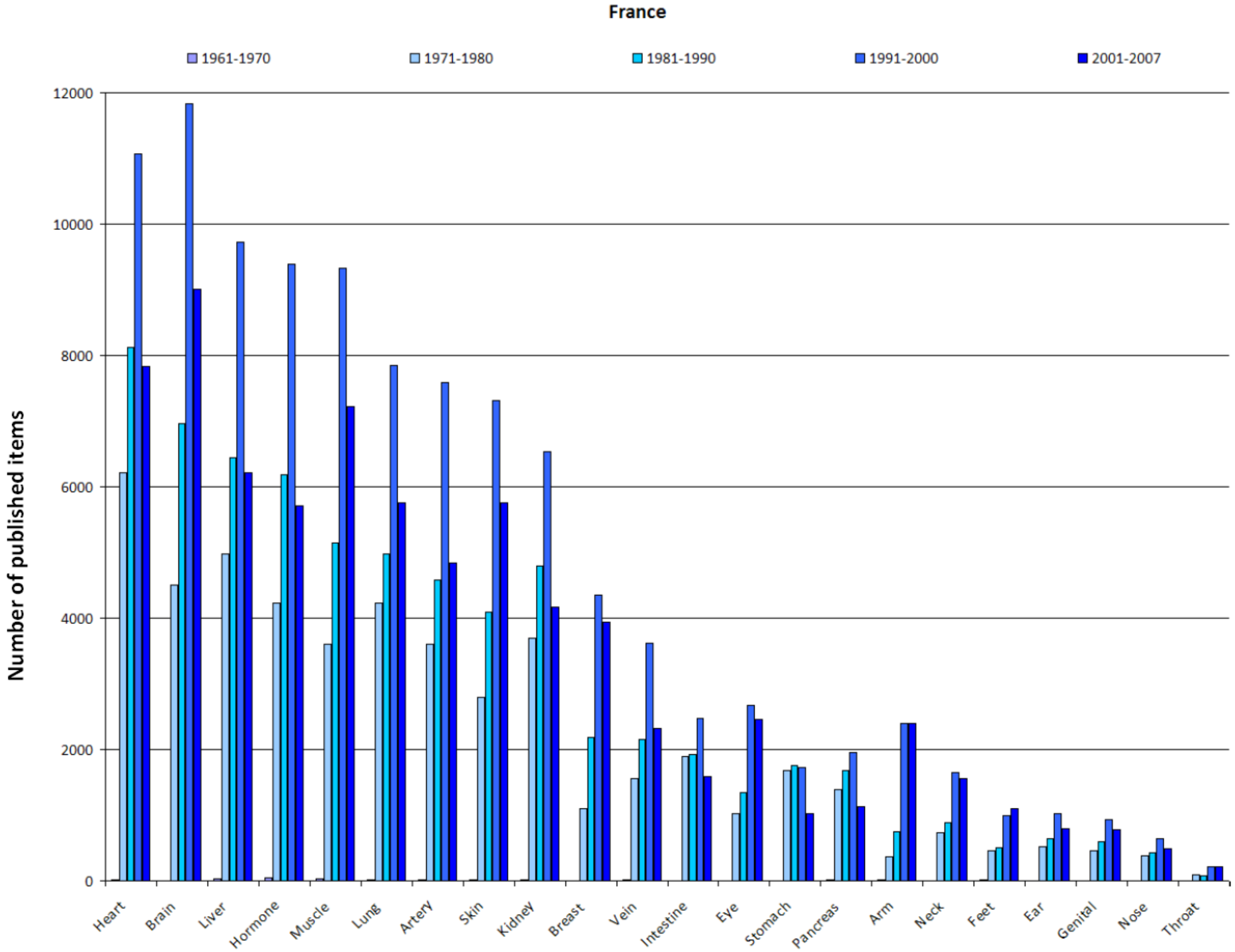
France
1961-1970
1971-1980
1981-1990
1991-2000
2001-2007
Number of published items
12000
10000
8000
6000
4000
2000
0
Heart
Brain
Liver
Hormone
Muscle
Lung
Artery
Skin
Kidney
Breast
Vein
Intestine
Eye
Stomach
Pancreas
Arm
Neck
Feet
Ear
Genital
Nose
Throat

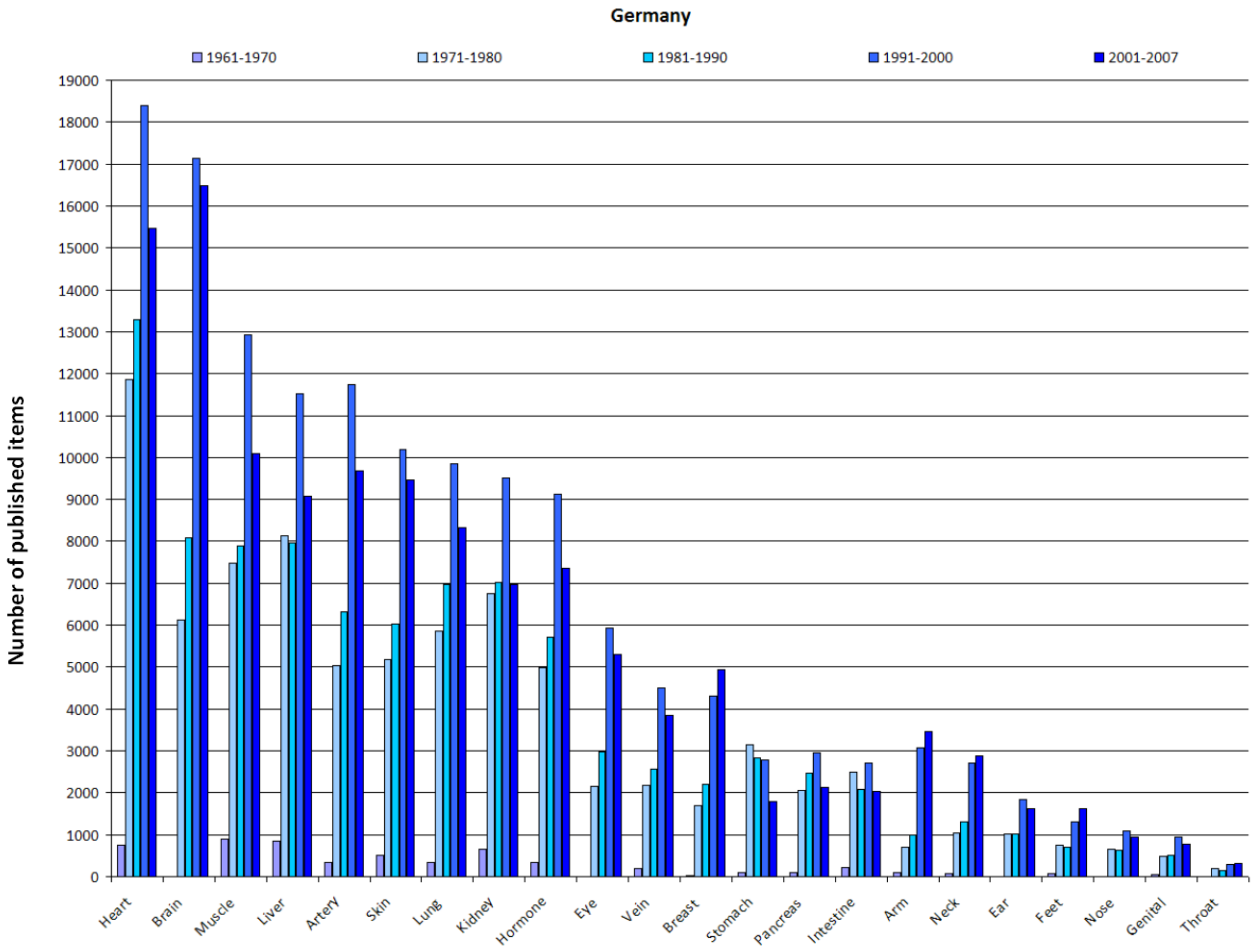

Germany
1961-1970
1971-1980
1981-1990
1991-2000
2001-2007
Number of published items
19000
18000
17000
16000
15000
14000
13000
12000
11000
10000
9000
8000
7000
6000
5000
4000
3000
2000
1000
0
Heart
Brain
Muscle
Liver
Artery
Skin
Lung
Kidney
Hormone
Eye
Vein
Breast
Stomach
Pancreas
Intestine
Arm
Neck
Ear
Feet
Nose
Genital
Throat

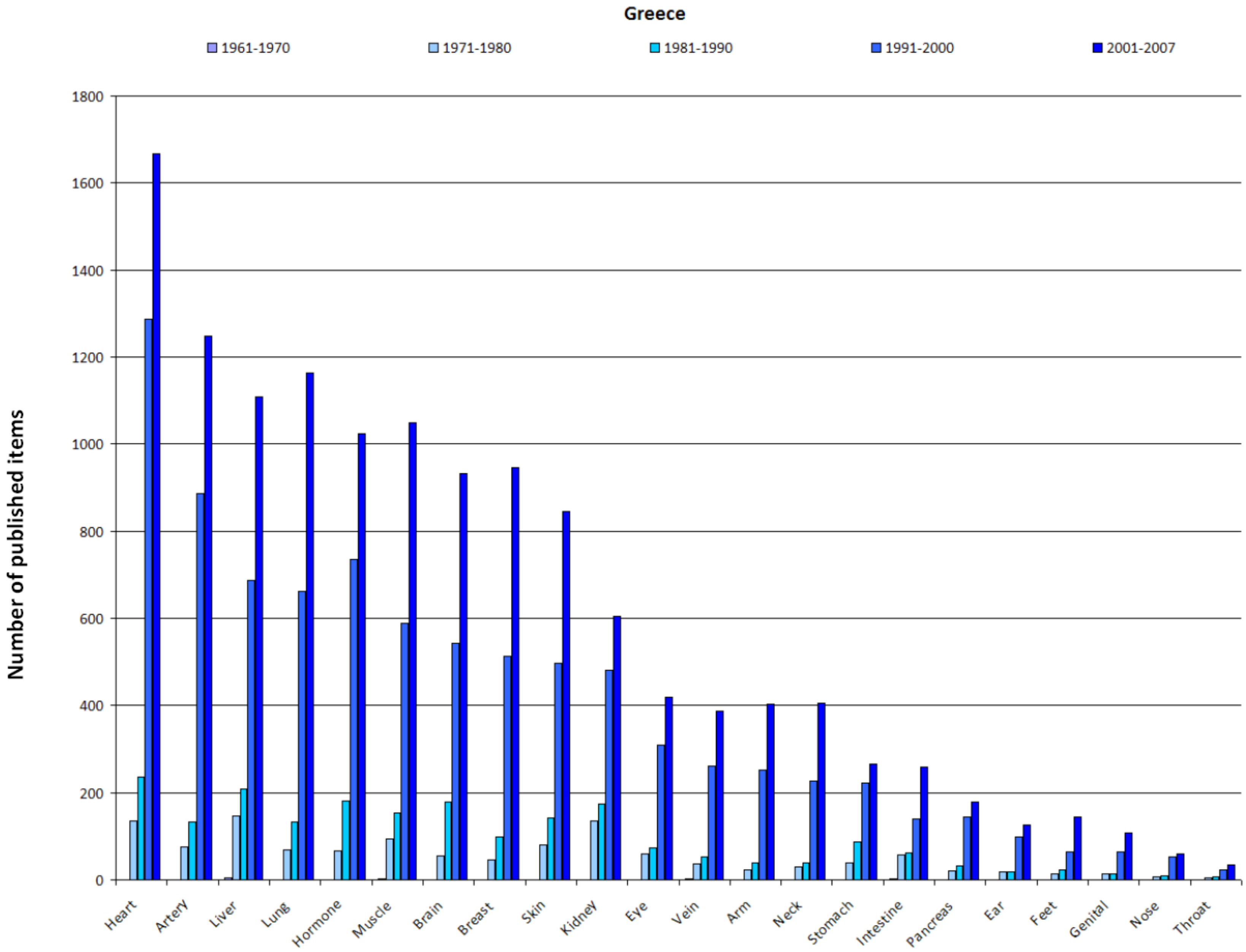
Greece
1961-1970
1971-1980
1981-1990
1991-2000
2001-2007
Number of published items
0
200
400
600
800
1000
1200
1400
1600
1800
Heart
Artery
Liver
Lung
Hormone
Muscle
Brain
Breast
Skin
Kidney
Eye
Vein
Arm
Neck
Stomach
Intestine
Pancreas
Ear
Feet
Genital
Nose
Throat

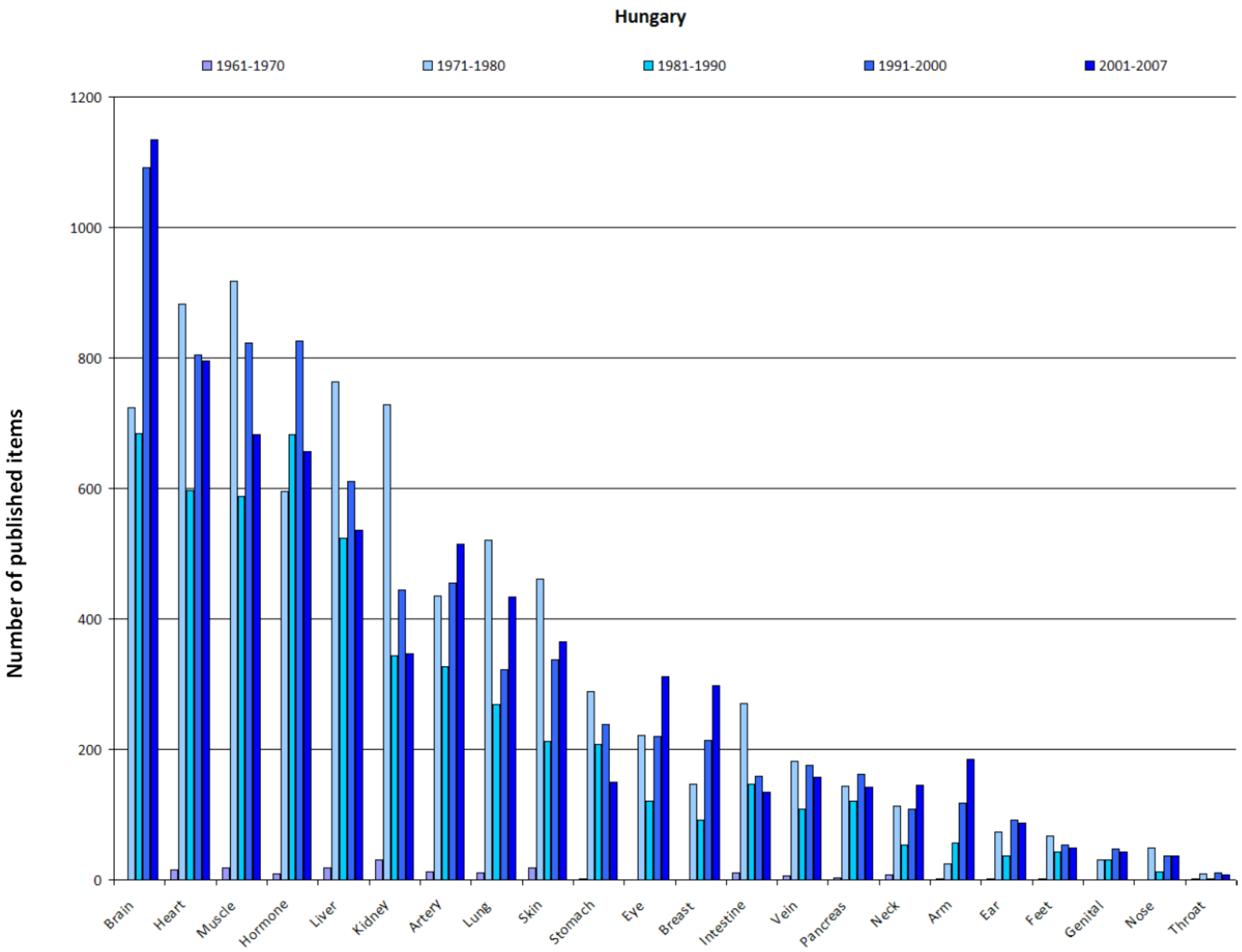
Hungary
1961-1970
1971-1980
1981-1990
1991-2000
2001-2007
Number of published items
1200
1000
800
600
400
200
0
Brain
Heart
Muscle
Hormone
Liver
Kidney
Artery
Lung
Skin
Stomach
Eye
Breast
Intestine
Vein
Pancreas
Neck
Arm
Ear
Feet
Genital
Nose
Throat

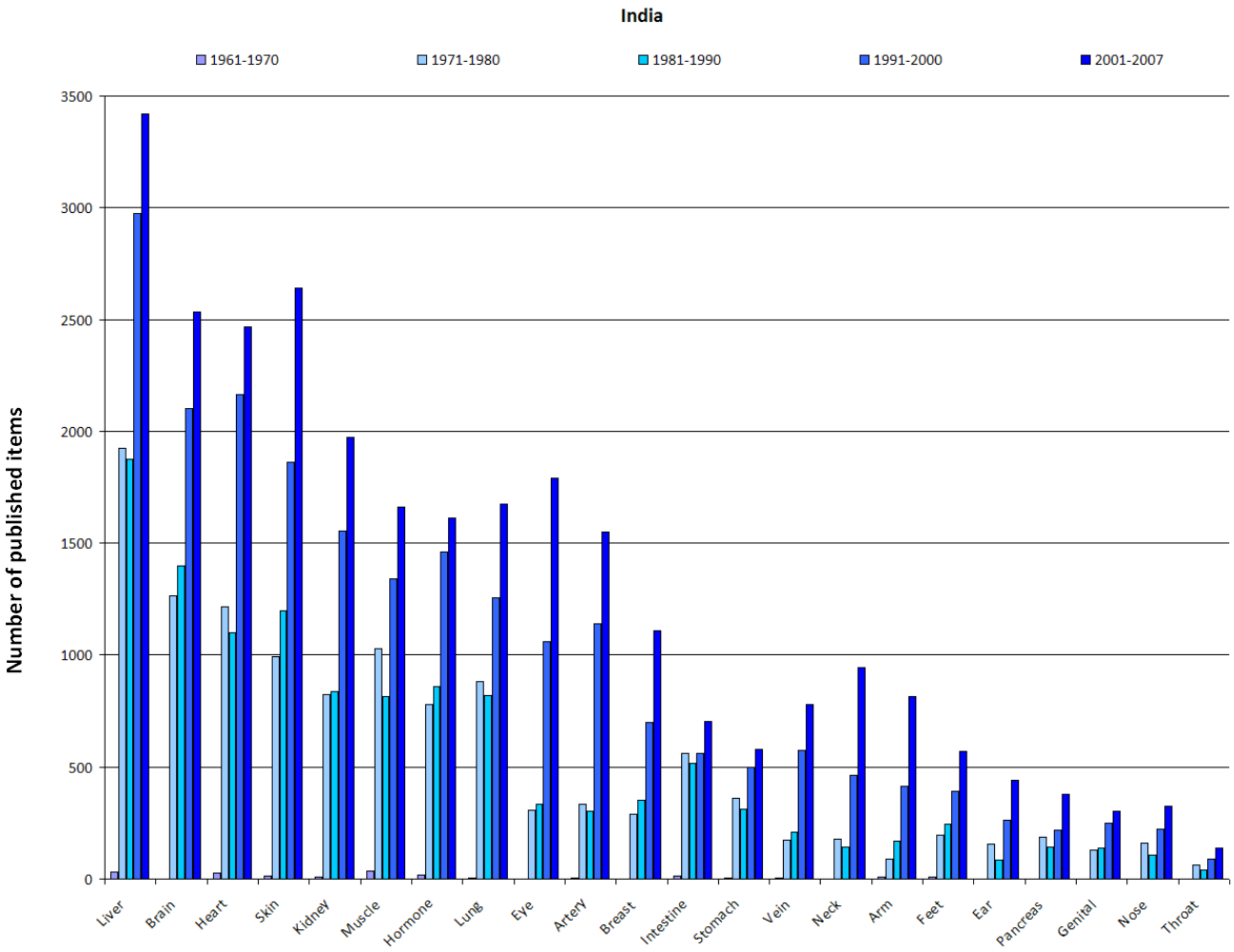
India
1961-1970
1971-1980
1981-1990
1991-2000
2001-2007
Number of published items
3500
3000
2500
2000
1500
1000
500
0
Liver
Brain
Heart
Skin
Kidney
Muscle
Hormone
Lung
Eye
Artery
Breast
Intestine
Stomach
Vein
Neck
Arm
Feet
Ear
Pancreas
Genital
Nose
Throat

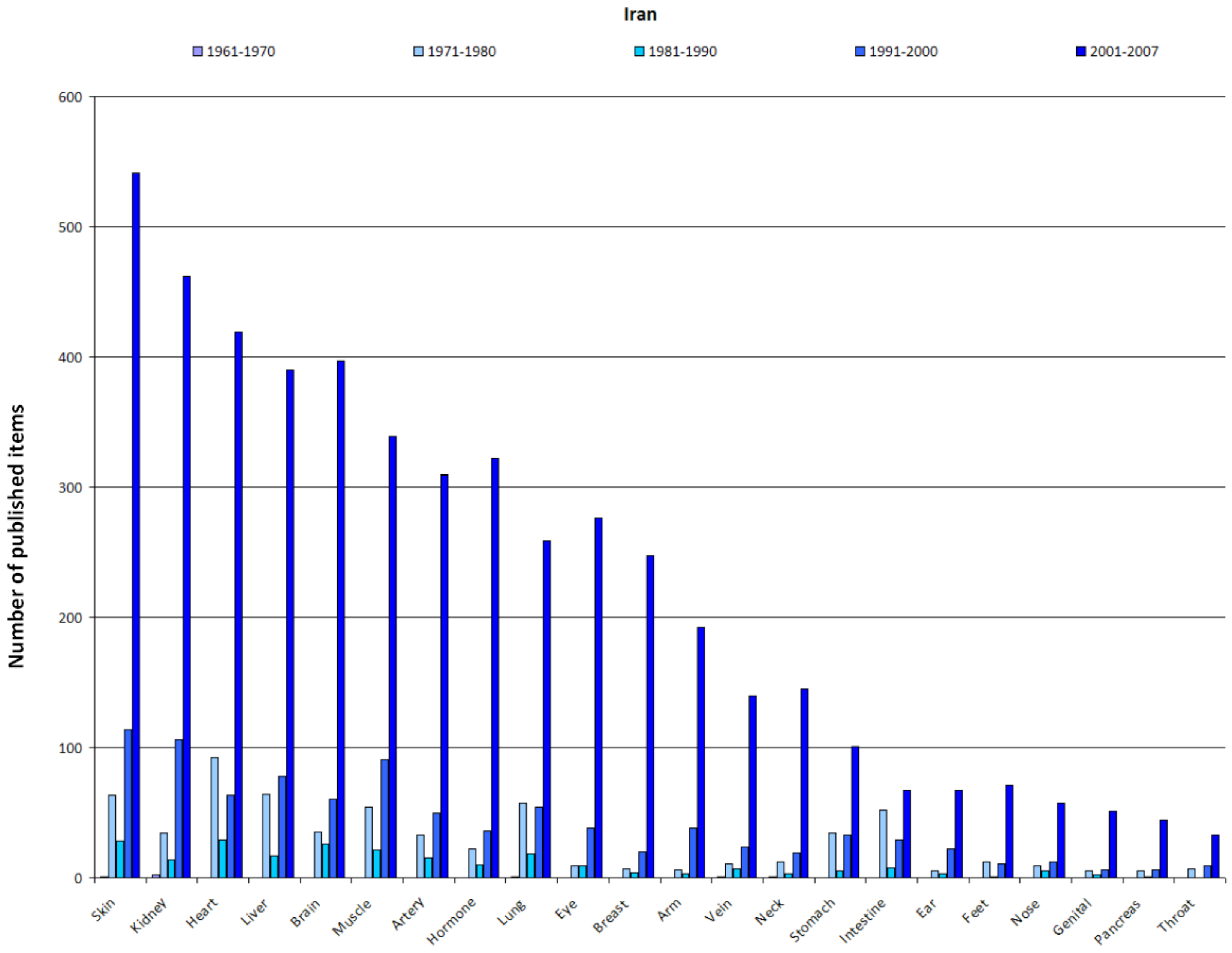
Iran
1961-1970
1971-1980
1981-1990
1991-2000
2001-2007
Number of published items
600
500
400
300
200
100
0
Skin
Kidney
Heart
Liver
Brain
Muscle
Artery
Hormone
Lung
Eye
Breast
Arm
Vein
Neck
Stomach
Intestine
Ear
Feet
Nose
Genital
Pancreas
Throat

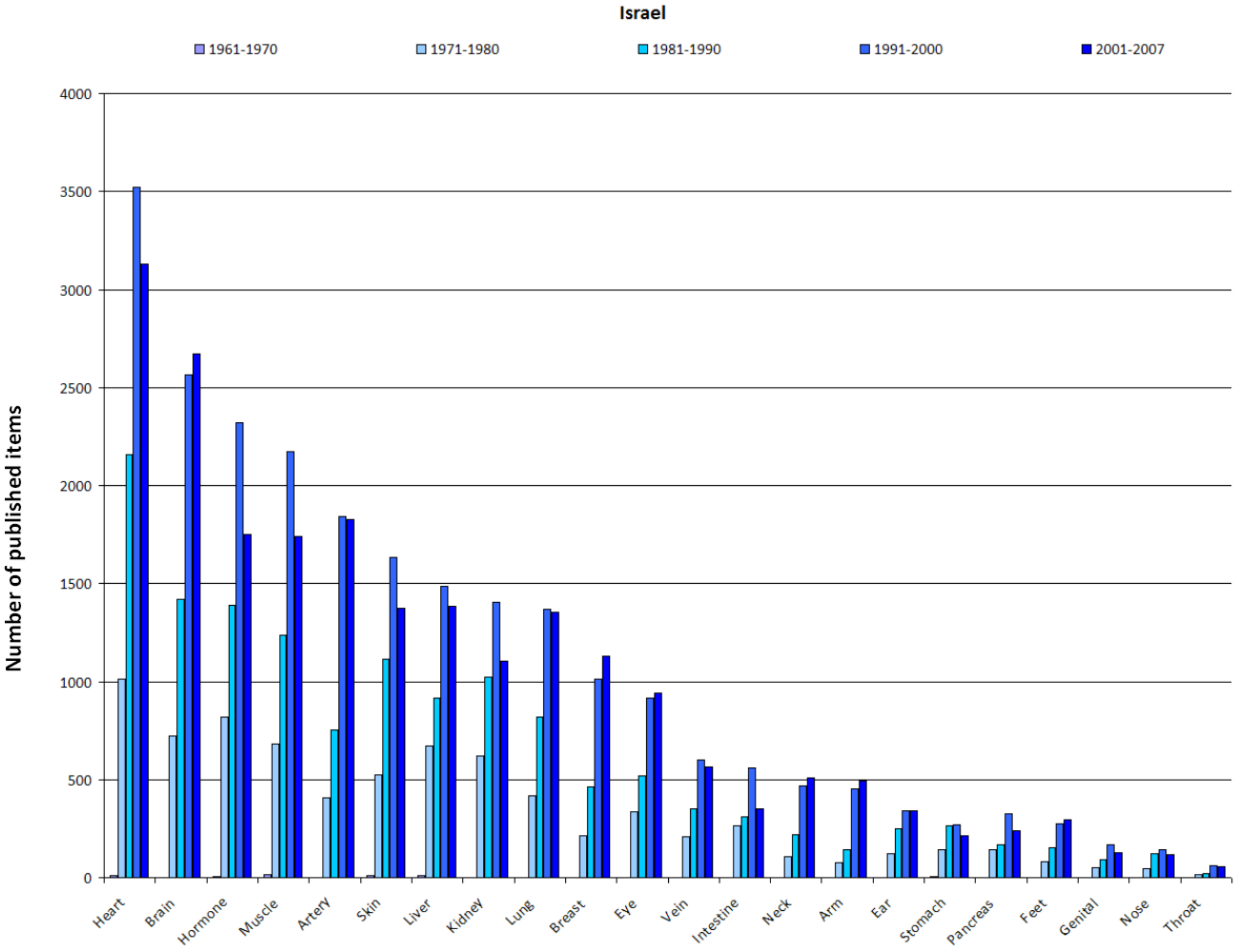

Israel
1961-1970
1971-1980
1981-1990
1991-2000
2001-2007
Number of published items
4000
3500
3000
2500
2000
1500
1000
500
0
Heart
Brain
Hormone
Muscle
Artery
Skin
Liver
Kidney
Lung
Breast
Eye
Vein
Intestine
Neck
Arm
Ear
Stomach
Pancreas
Feet
Genital
Nose
Throat

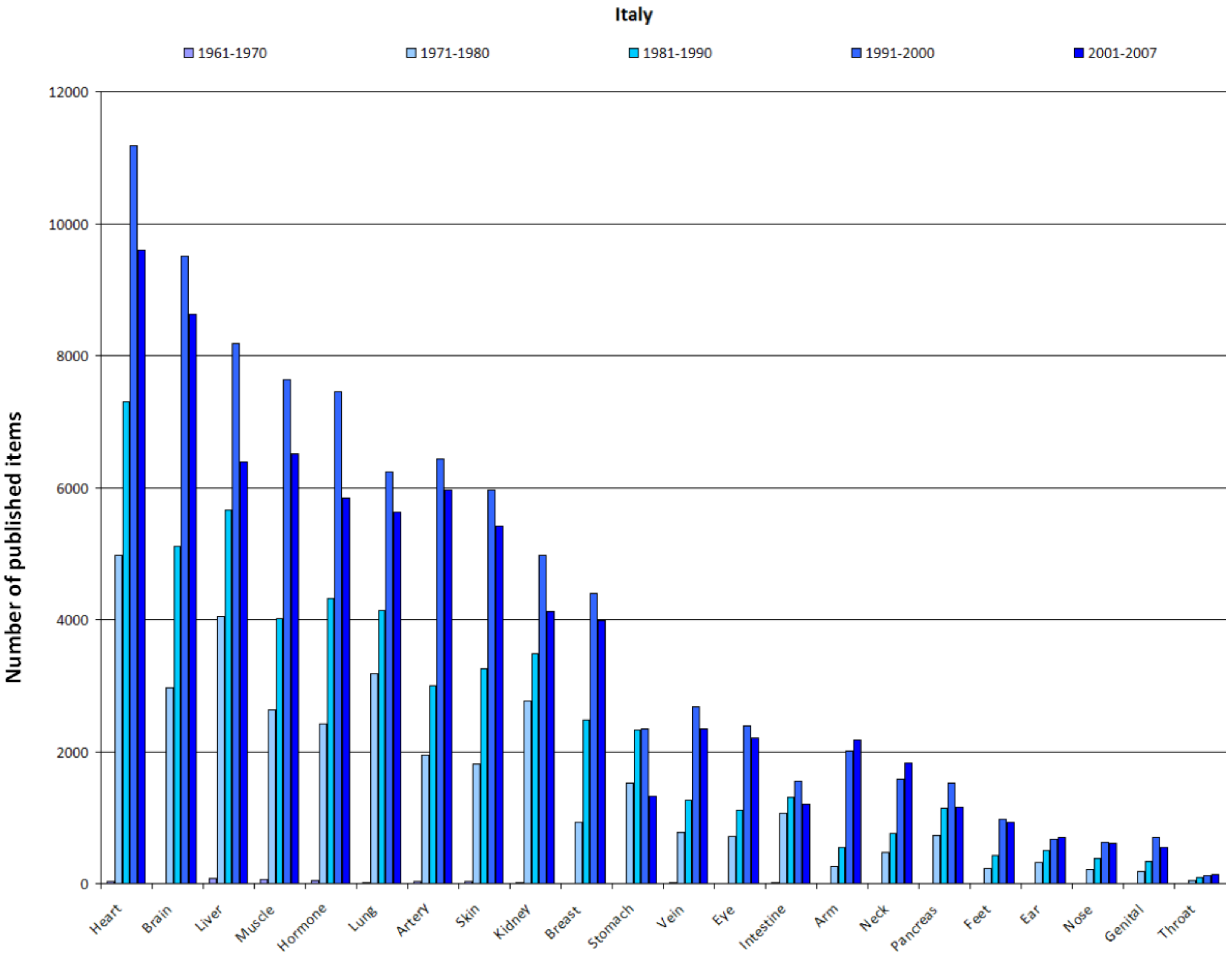
Italy
1961-1970
1971-1980
1981-1990
1991-2000
2001-2007
Number of published items
12000
10000
8000
6000
4000
2000
0
Heart
Brain
Liver
Muscle
Hormone
Lung
Artery
Skin
Kidney
Breast
Stomach
Vein
Eye
Intestine
Arm
Neck
Pancreas
Feet
Ear
Nose
Genital
Throat

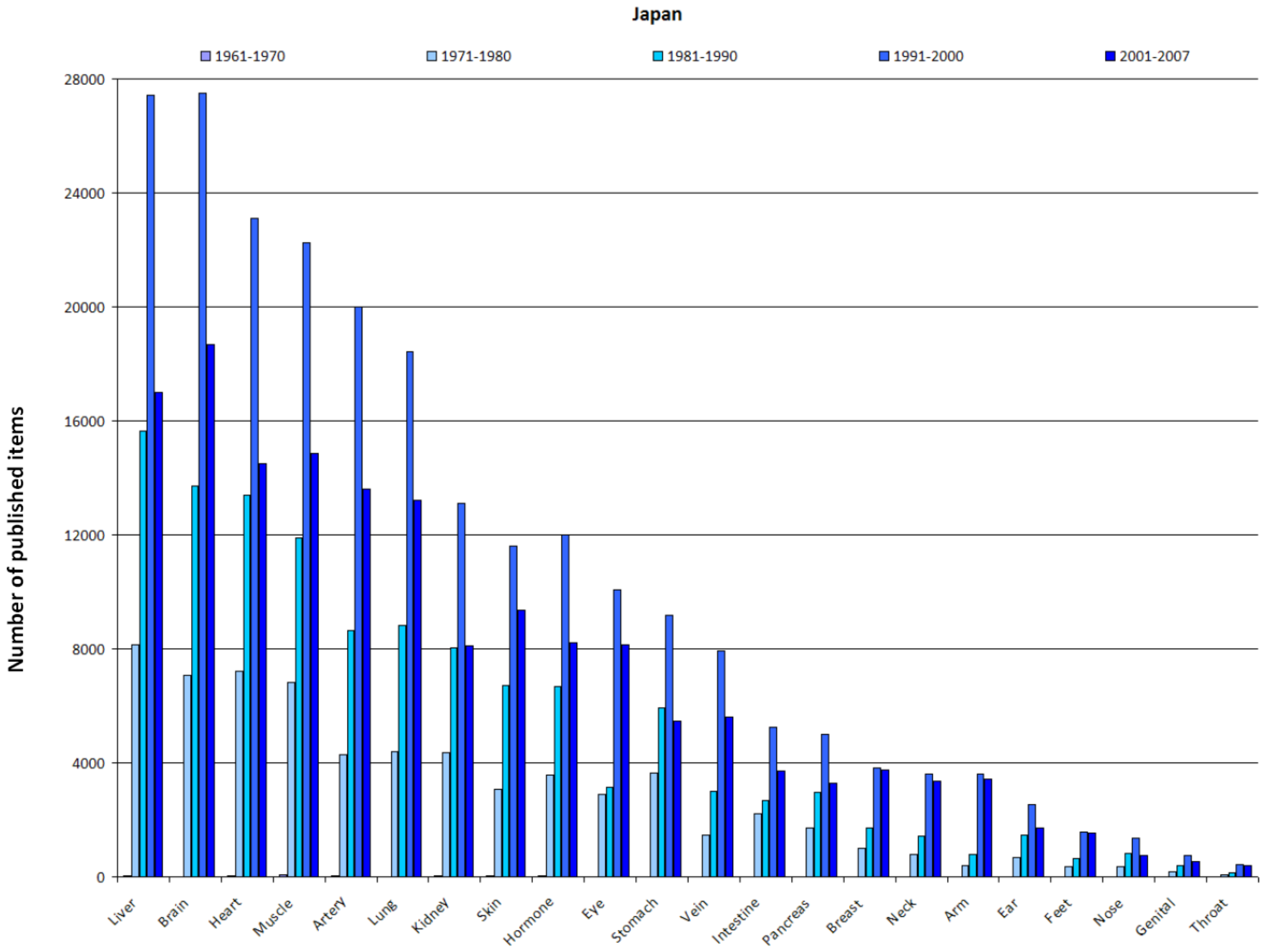
Japan
1961-1970
1971-1980
1981-1990
1991-2000
2001-2007
Number of published items
0
4000
8000
12000
16000
20000
24000
28000
Liver
Brain
Heart
Muscle
Artery
Lung
Kidney
Skin
Hormone
Eye
Stomach
Vein
Intestine
Pancreas
Breast
Neck
Arm
Ear
Feet
Nose
Genital
Throat

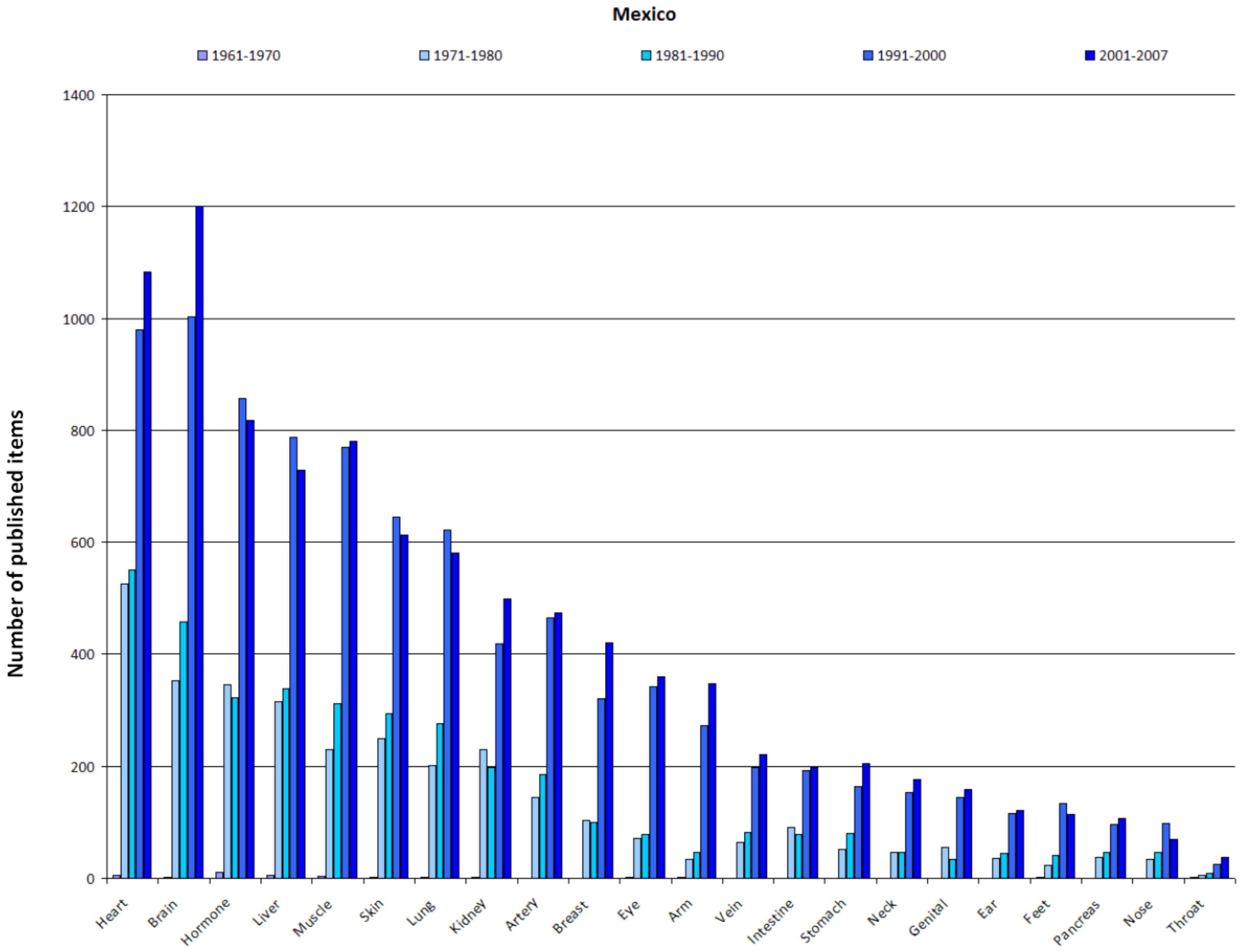

Mexico
1961-1970
1971-1980
1981-1990
1991-2000
2001-2007
Number of published items
1400
1200
1000
800
600
400
200
0
Heart
Brain
Hormone
Liver
Muscle
Skin
Lung
Kidney
Artery
Breast
Eye
Arm
Vein
Intestine
Stomach
Neck
Genital
Ear
Feet
Pancreas
Nose
Throat

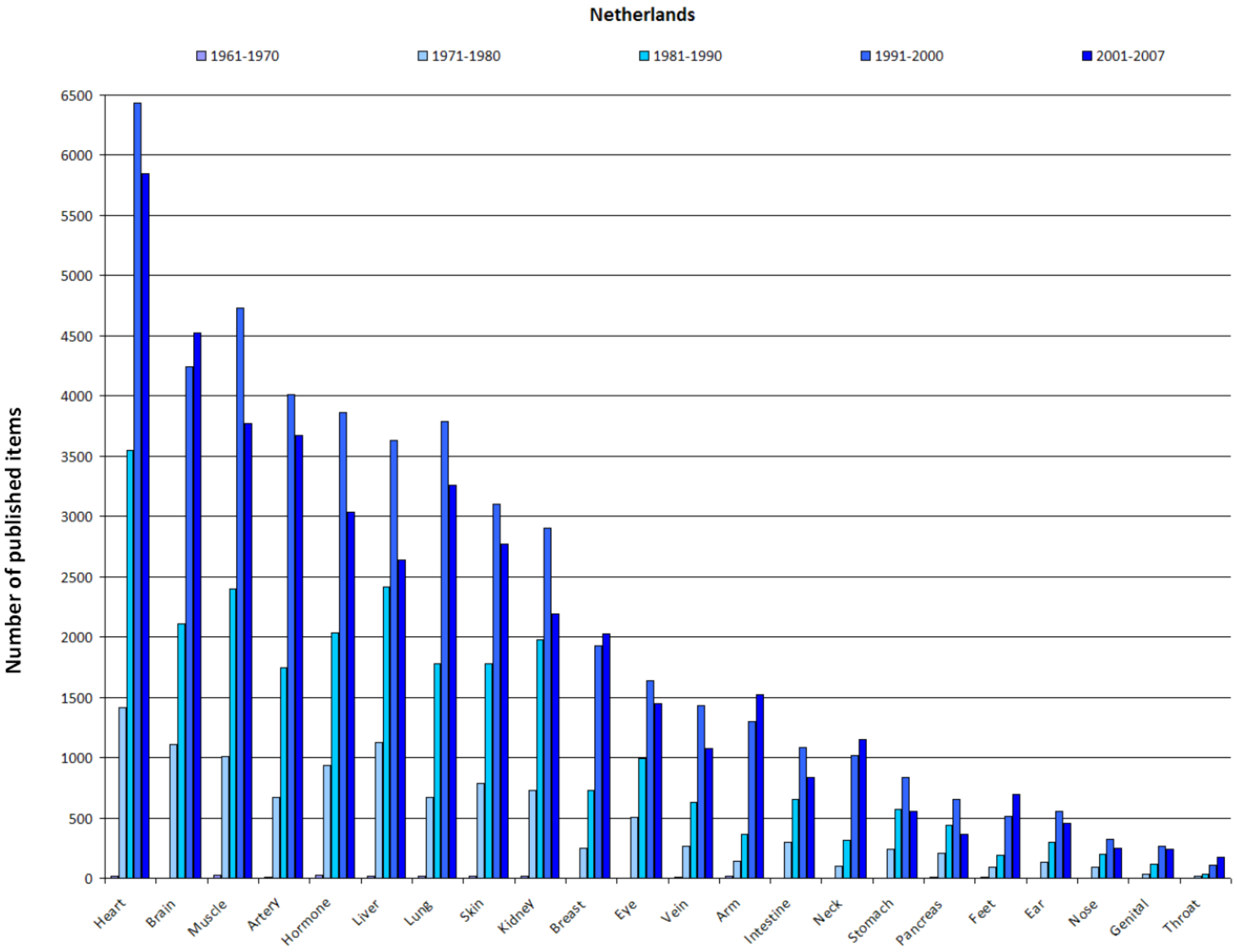

Netherlands
1961-1970
1971-1980
1981-1990
1991-2000
2001-2007
Number of published items
6500
6000
5500
5000
4500
4000
3500
3000
2500
2000
1500
1000
500
0
Heart
Brain
Muscle
Artery
Hormone
Liver
Lung
Skin
Kidney
Breast
Eye
Vein
Arm
Intestine
Neck
Stomach
Pancreas
Feet
Ear
Nose
Genital
Throat

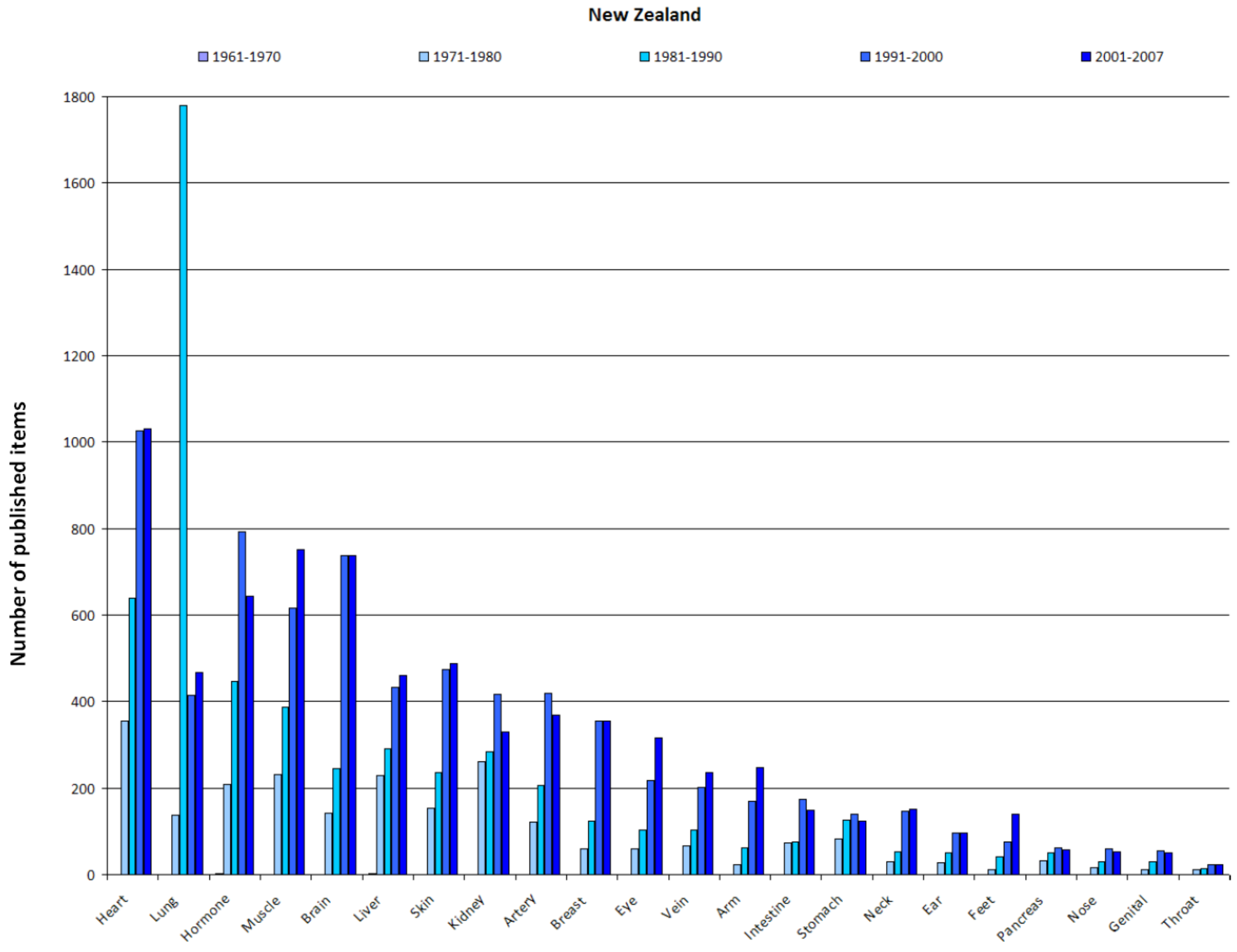
New Zealand
1961-1970
1971-1980
1981-1990
1991-2000
2001-2007
Number of published items
1800
1600
1400
1200
1000
800
600
400
200
0
Heart
Lung
Hormone
Muscle
Brain
Liver
Skin
Kidney
Artery
Breast
Eye
Vein
Arm
Intestine
Stomach
Neck
Ear
Feet
Pancreas
Nose
Genital
Throat

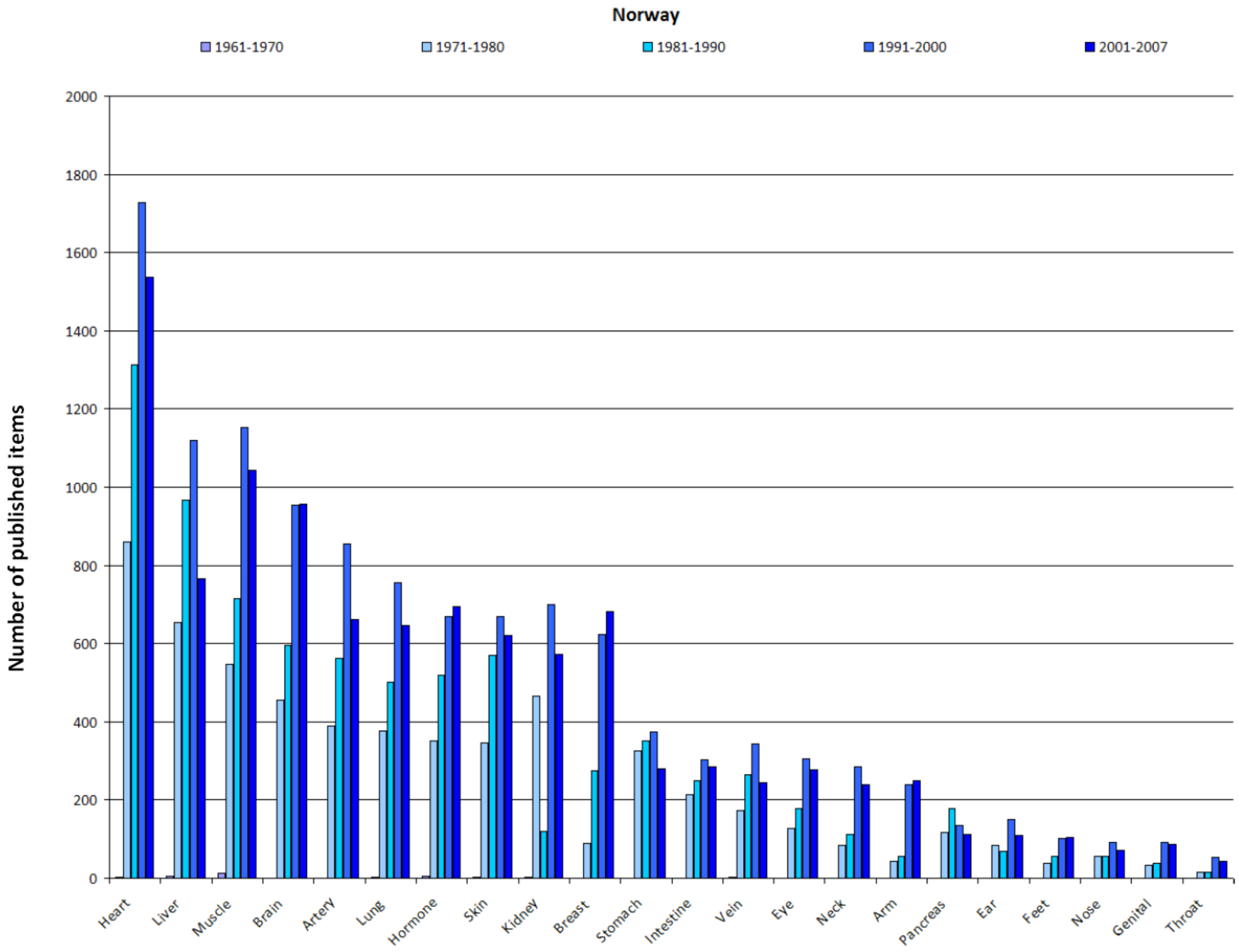
Norway
1961-1970
1971-1980
1981-1990
1991-2000
2001-2007
Number of published items
2000
1800
1600
1400
1200
1000
800
600
400
200
0
Heart
Liver
Muscle
Brain
Artery
Lung
Hormone
Skin
Kidney
Breast
Stomach
Intestine
Vein
Eye
Neck
Arm
Pancreas
Ear
Feet
Nose
Genital
Throat

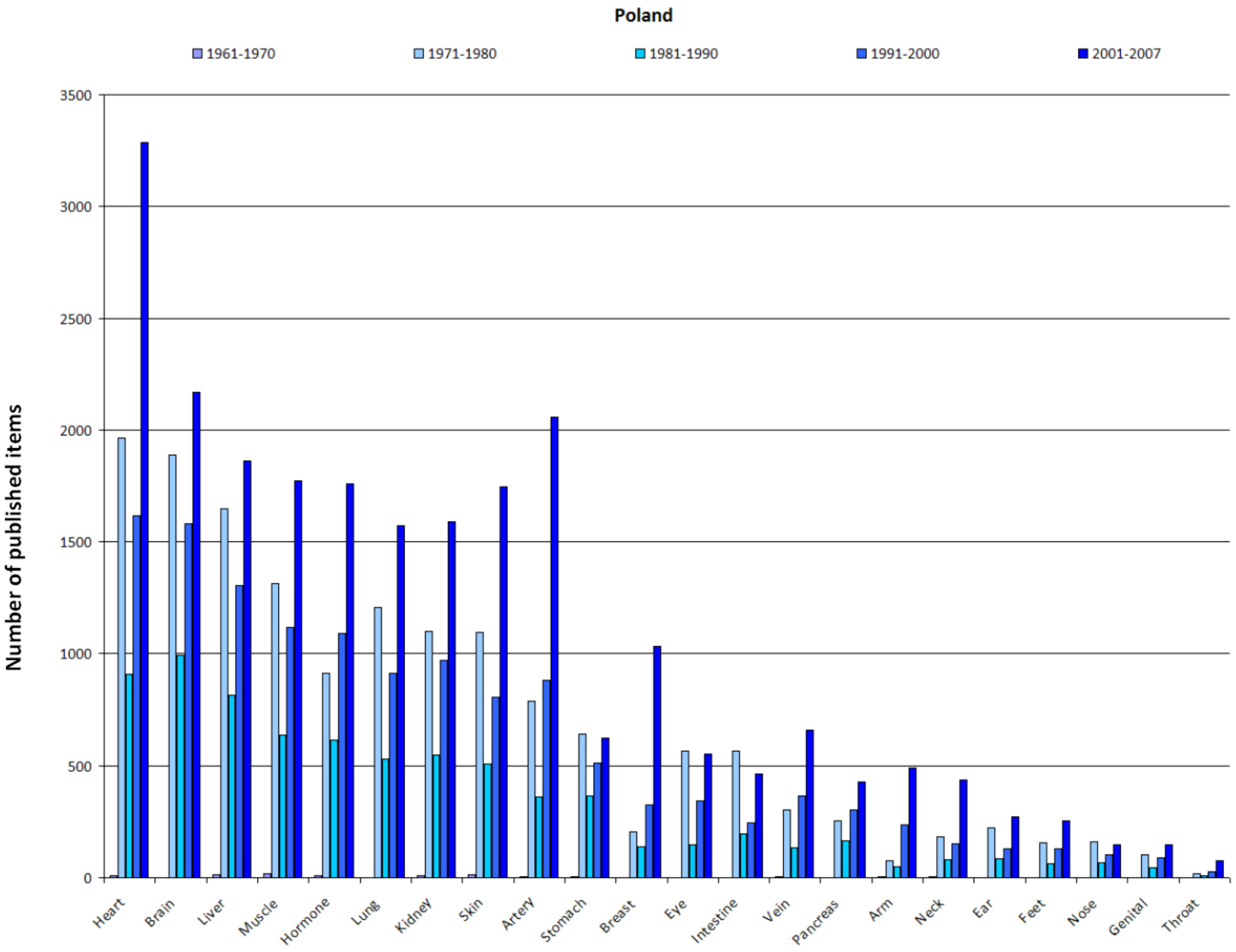

Poland
1961-1970
1971-1980
1981-1990
1991-2000
2001-2007
Number of published items
3500
3000
2500
2000
1500
1000
500
0
Heart
Brain
Liver
Muscle
Hormone
Lung
Kidney
Skin
Artery
Stomach
Breast
Eye
Intestine
Vein
Pancreas
Arm
Neck
Ear
Feet
Nose
Genital
Throat

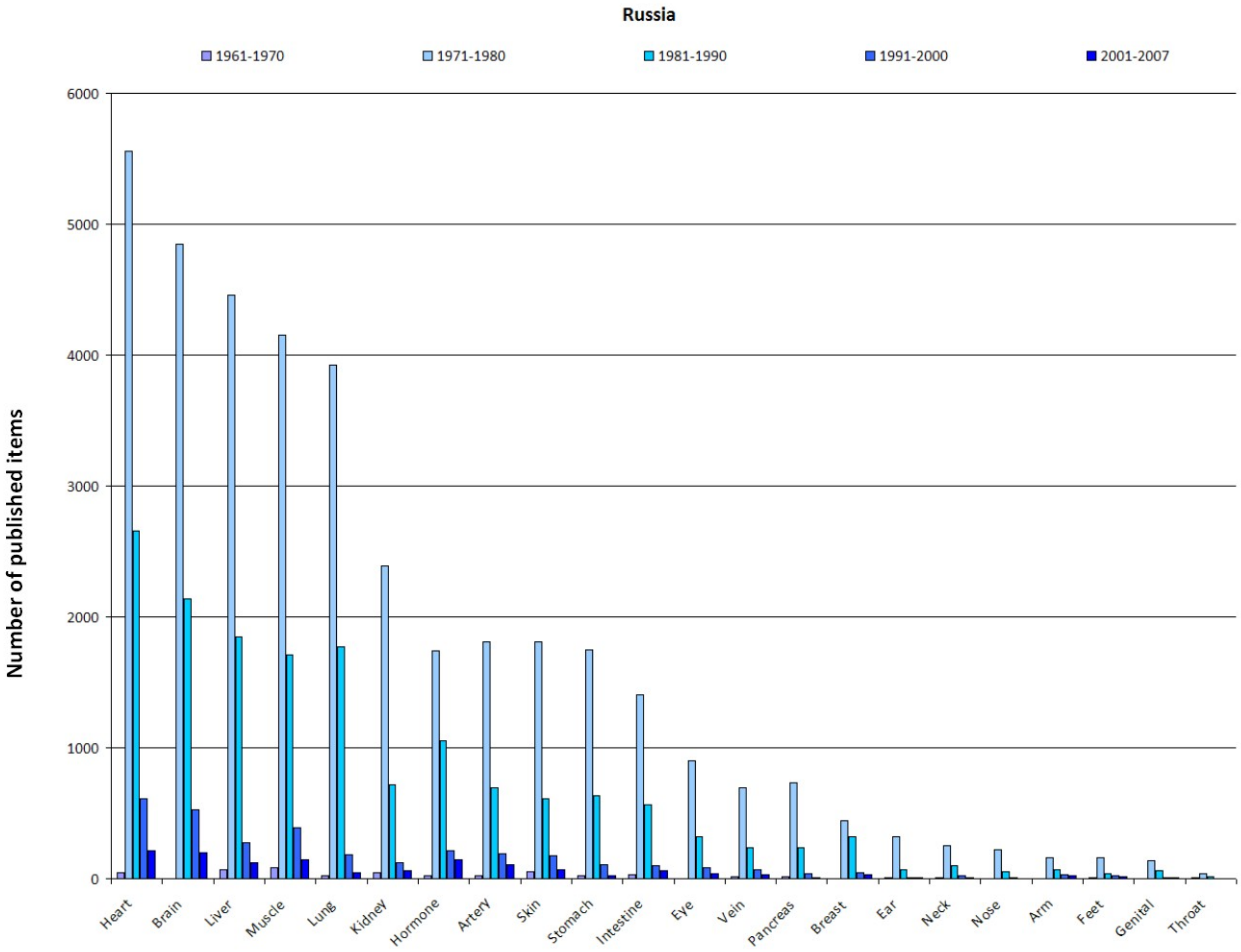
Russia
1961-1970
1971-1980
1981-1990
1991-2000
2001-2007
Number of published items
6000
5000
4000
3000
2000
1000
0
Heart
Brain
Liver
Muscle
Lung
Kidney
Hormone
Artery
Skin
Stomach
Intestine
Eye
Vein
Pancreas
Breast
Ear
Neck
Nose
Arm
Feet
Genital
Throat

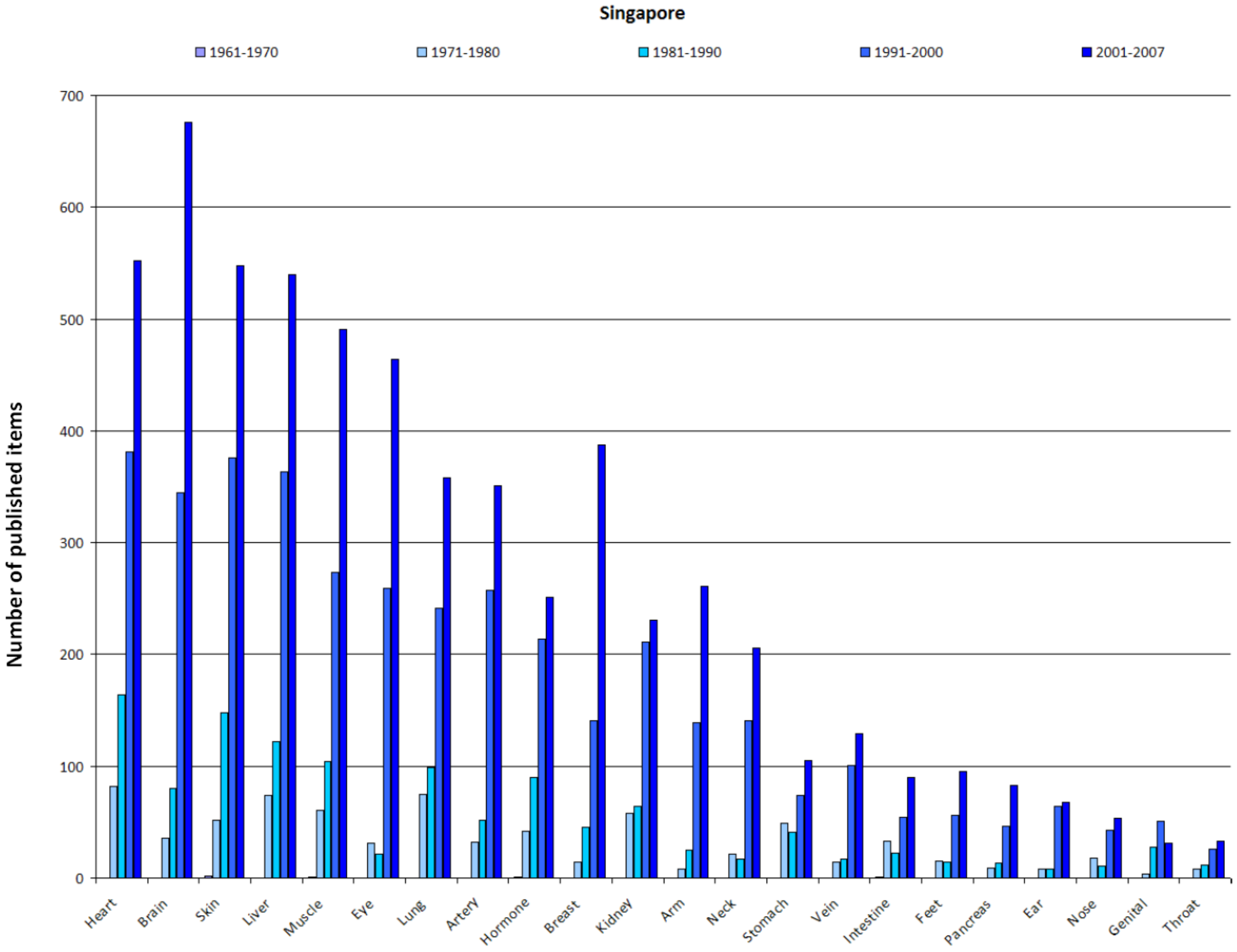
Singapore
1961-1970
1971-1980
1981-1990
1991-2000
2001-2007
Number of published items
700
600
500
400
300
200
100
0
Heart
Brain
Skin
Liver
Muscle
Eye
Lung
Artery
Hormone
Breast
Kidney
Arm
Neck
Stomach
Vein
Intestine
Feet
Pancreas
Ear
Nose
Genital
Throat

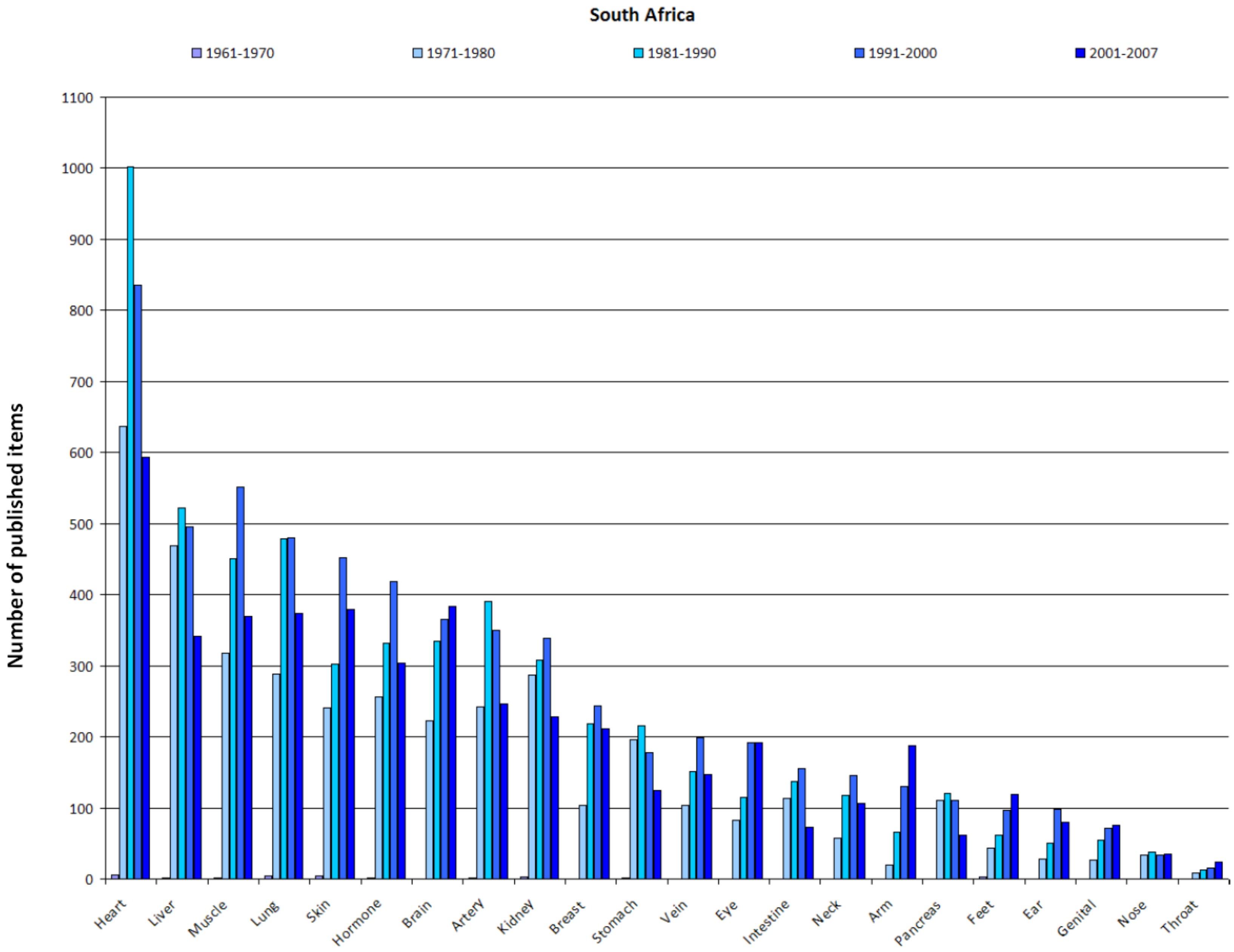
South Africa
1961-1970
1971-1980
1981-1990
1991-2000
2001-2007
Number of published items
1100
1000
900
800
700
600
500
400
300
200
100
0
Heart
Liver
Muscle
Lung
Skin
Hormone
Brain
Artery
Kidney
Breast
Stomach
Vein
Eye
Intestine
Neck
Arm
Pancreas
Feet
Ear
Genital
Nose
Throat

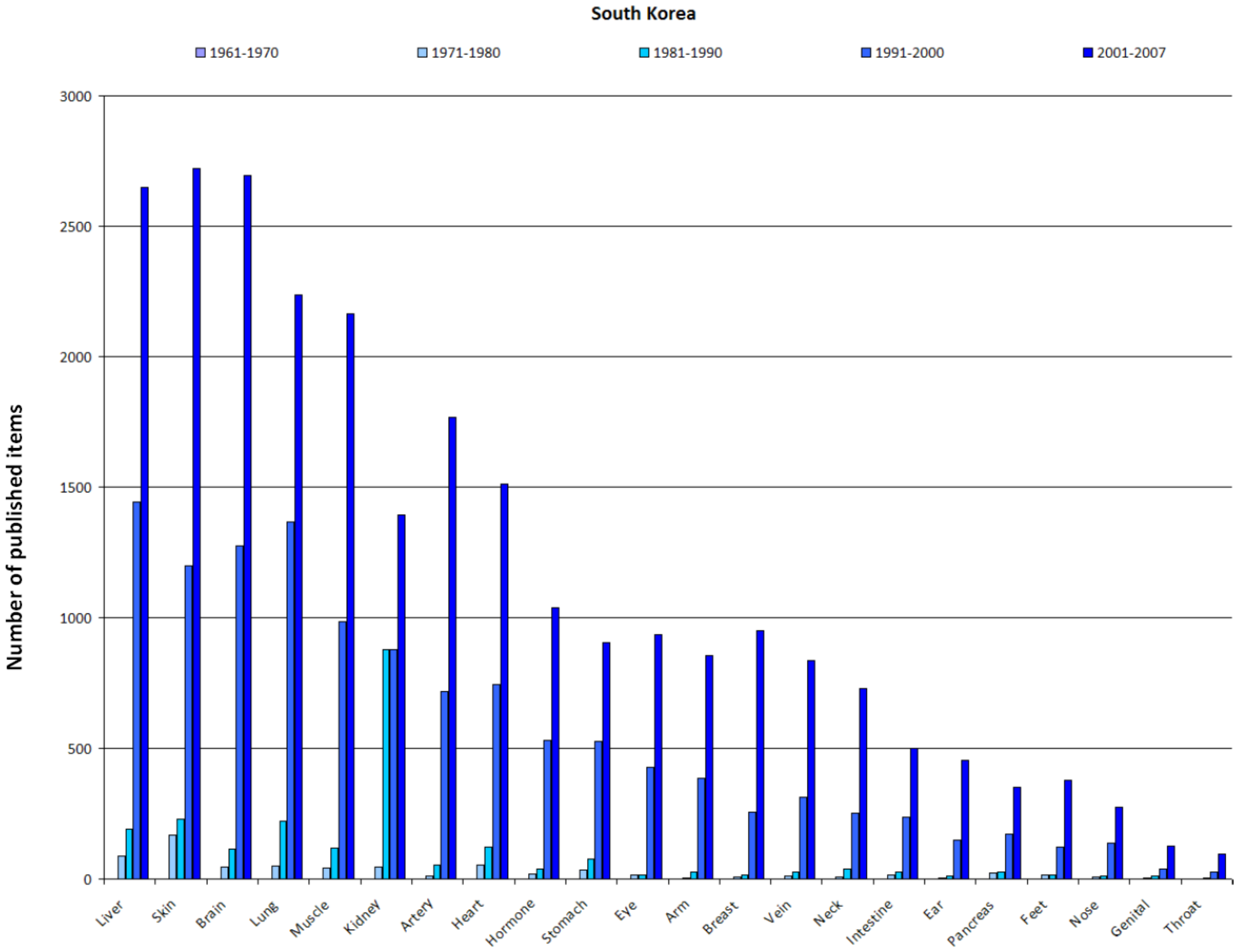
South Korea
1961-1970
1971-1980
1981-1990
1991-2000
2001-2007
Number of published items
3000
2500
2000
1500
1000
500
0
Liver
Skin
Brain
Lung
Muscle
Kidney
Artery
Heart
Hormone
Stomach
Eye
Arm
Breast
Vein
Neck
Intestine
Ear
Pancreas
Feet
Nose
Genital
Throat

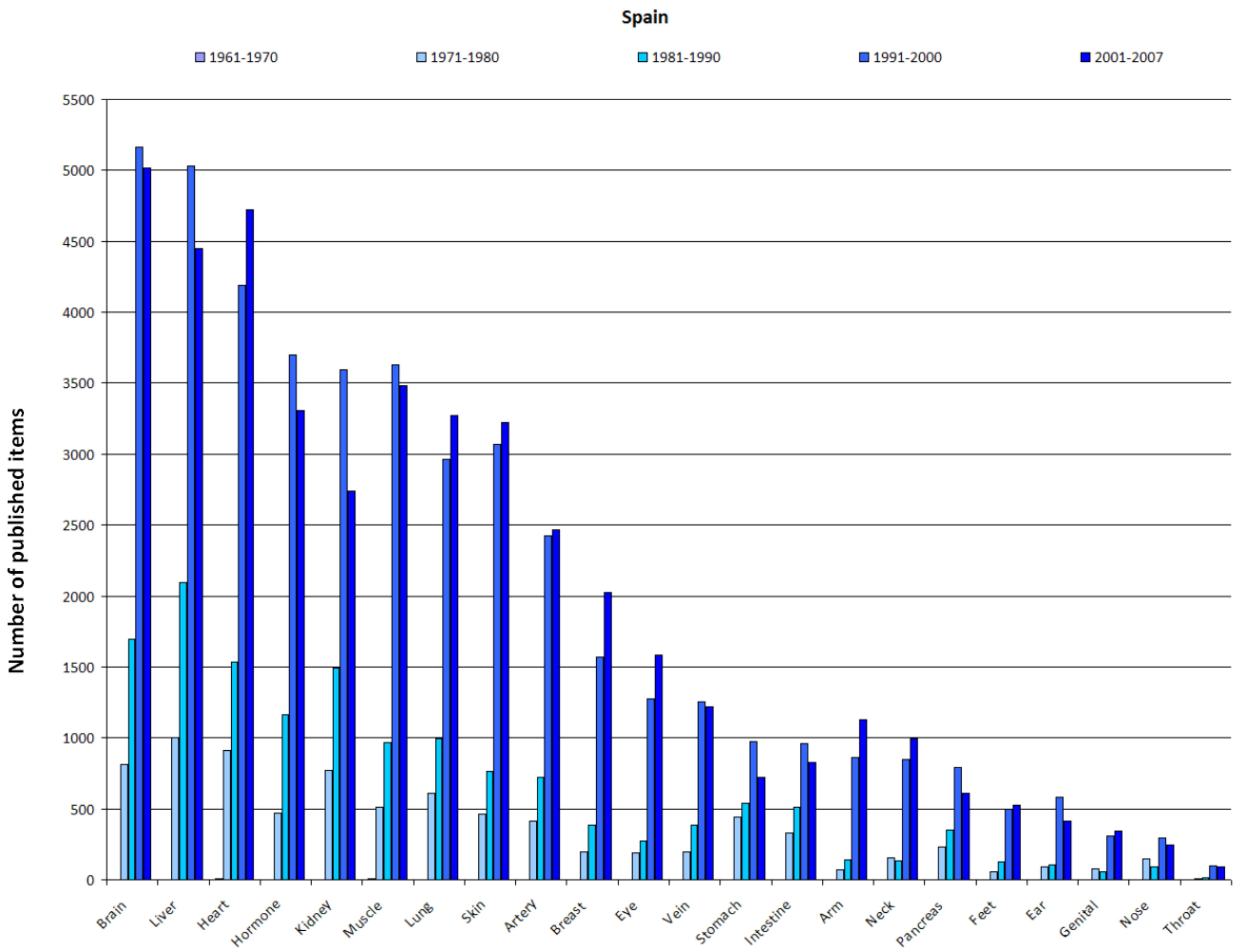

Spain
1961-1970
1971-1980
1981-1990
1991-2000
2001-2007
Number of published items
5500
5000
4500
4000
3500
3000
2500
2000
1500
1000
500
0
Brain
Liver
Heart
Hormone
Kidney
Muscle
Lung
Skin
Artery
Breast
Eye
Vein
Stomach
Intestine
Arm
Neck
Pancreas
Feet
Ear
Genital
Nose
Throat

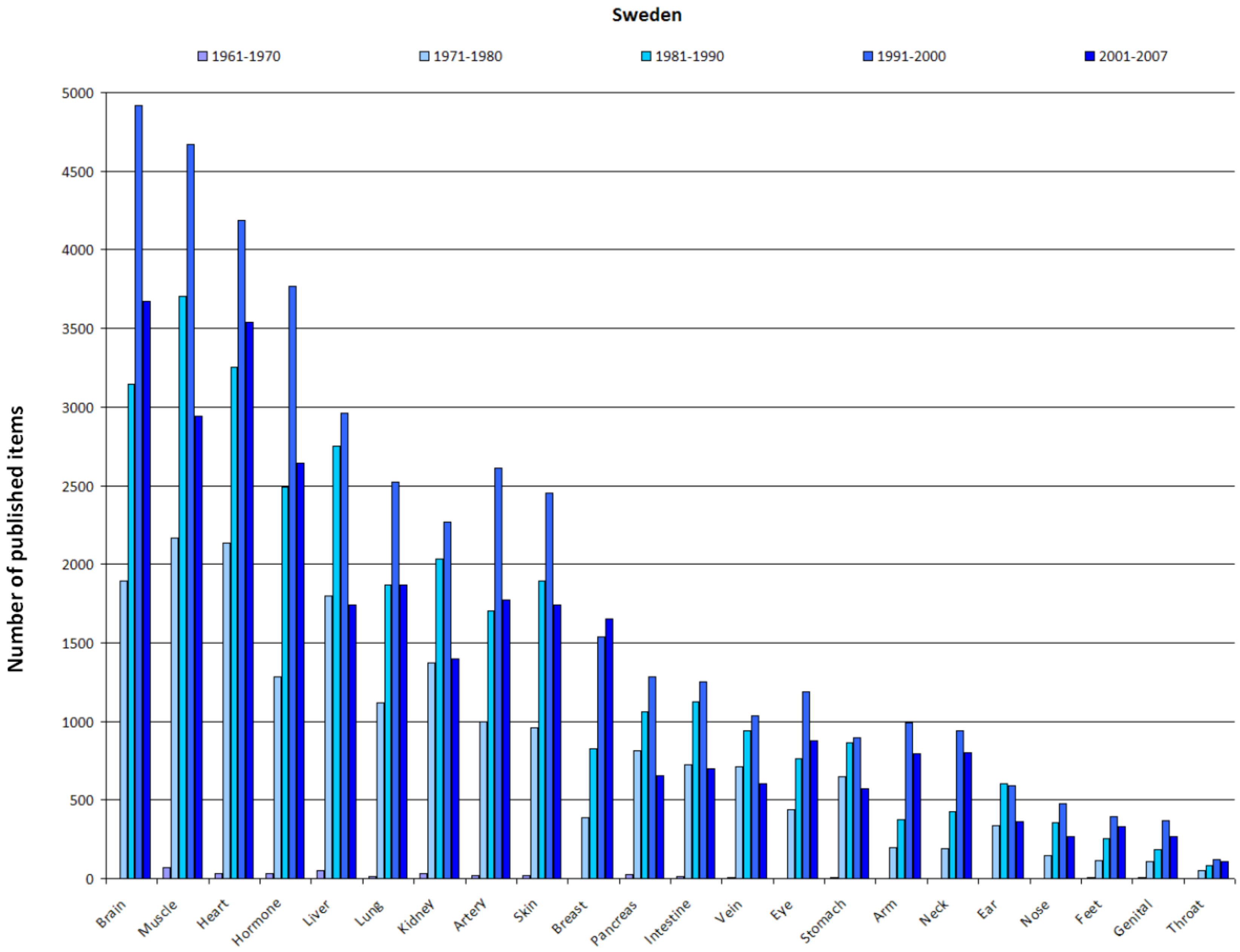

Sweden
1961-1970
1971-1980
1981-1990
1991-2000
2001-2007
Number of published items
5000
4500
4000
3500
3000
2500
2000
1500
1000
500
0
Brain
Muscle
Heart
Hormone
Liver
Lung
Kidney
Artery
Skin
Breast
Pancreas
Intestine
Vein
Eye
Stomach
Arm
Neck
Ear
Nose
Feet
Genital
Throat

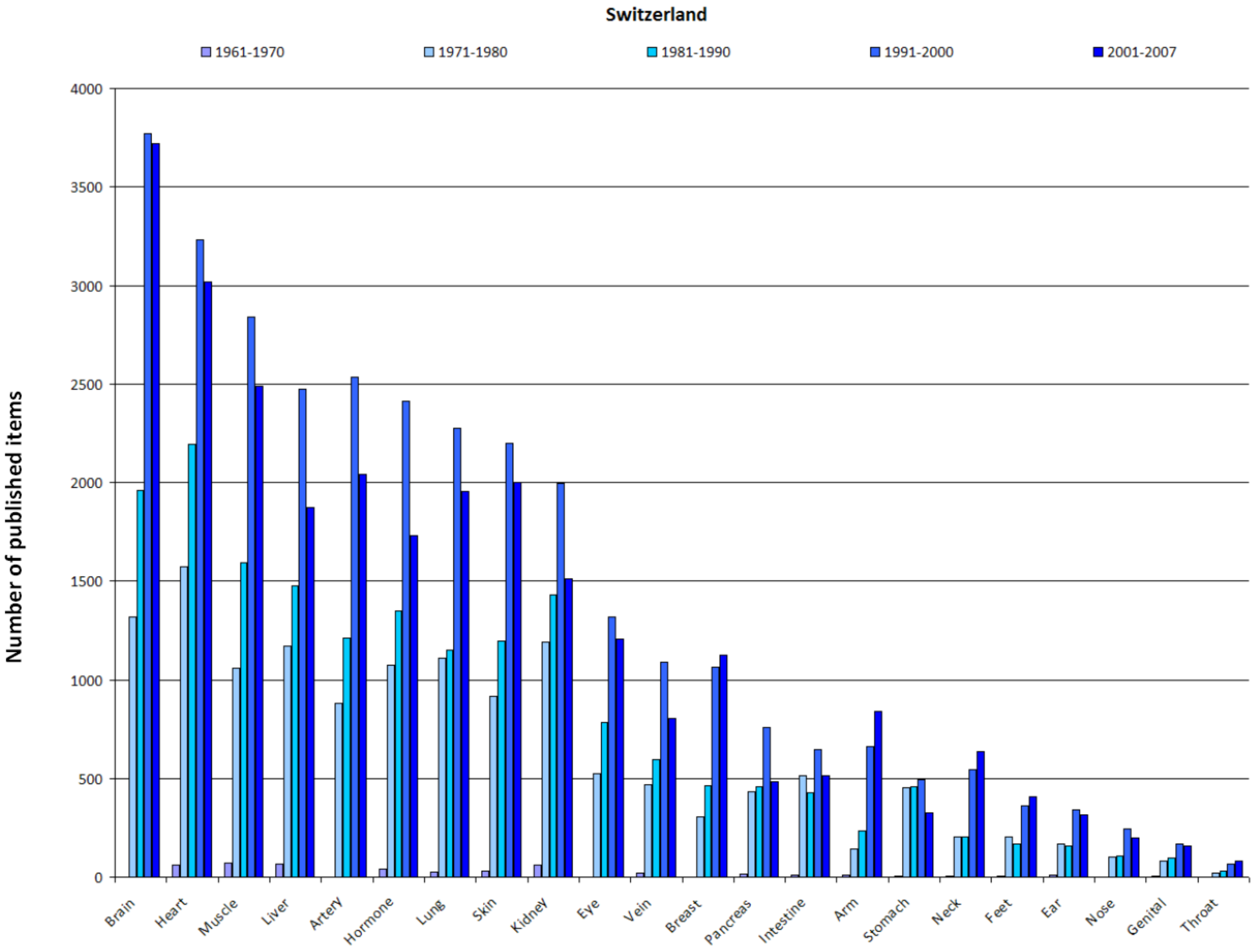
Switzerland
1961-1970
1971-1980
1981-1990
1991-2000
2001-2007
Number of published items
4000
3500
3000
2500
2000
1500
1000
500
0
Brain
Heart
Muscle
Liver
Artery
Hormone
Lung
Skin
Kidney
Eye
Vein
Breast
Pancreas
Intestine
Arm
Stomach
Neck
Feet
Ear
Nose
Genital
Throat

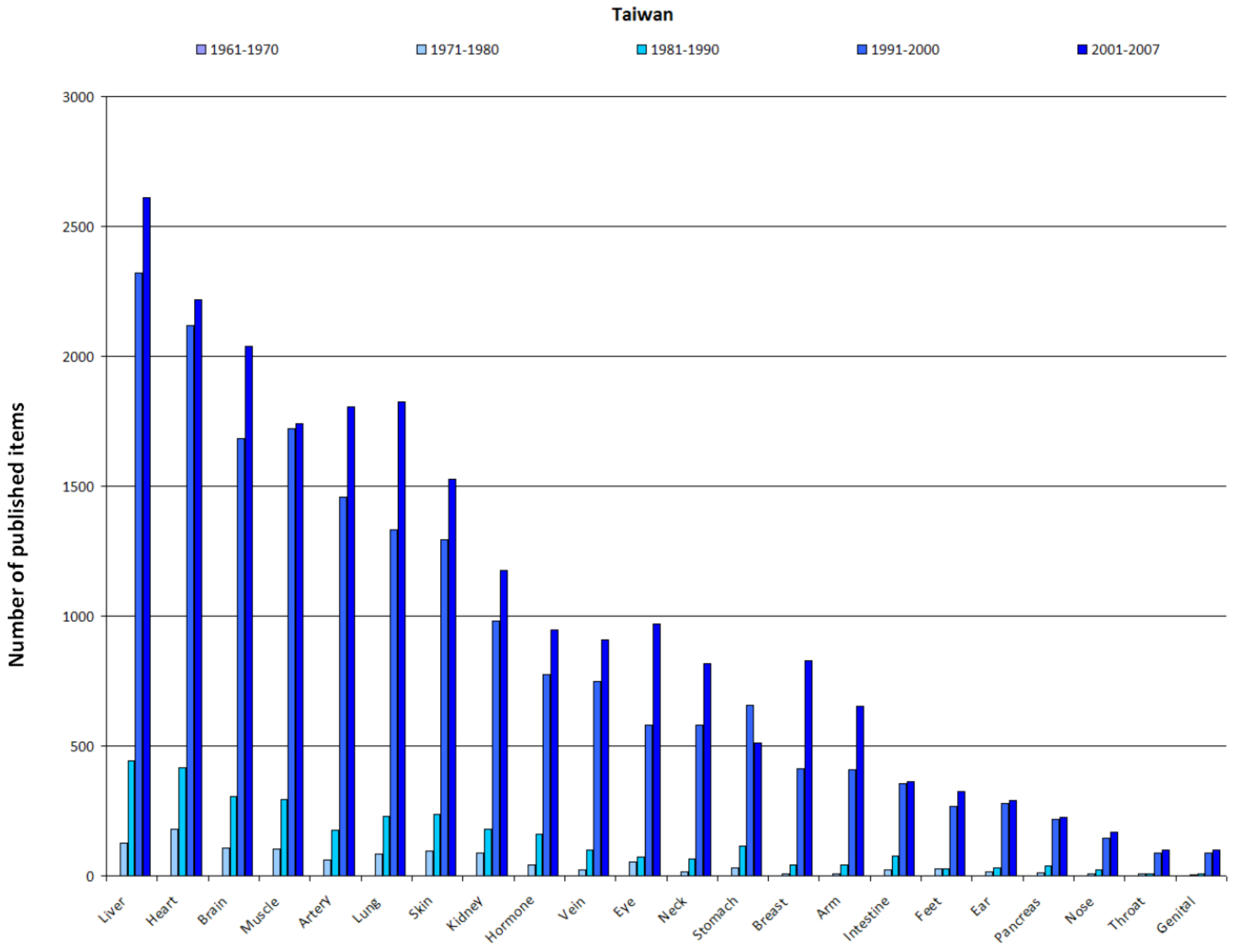
Taiwan
1961-1970
1971-1980
1981-1990
1991-2000
2001-2007
Number of published items
3000
2500
2000
1500
1000
500
0
Liver
Heart
Brain
Muscle
Artery
Lung
Skin
Kidney
Hormone
Vein
Eye
Neck
Stomach
Breast
Arm
Intestine
Feet
Ear
Pancreas
Nose
Throat
Genital

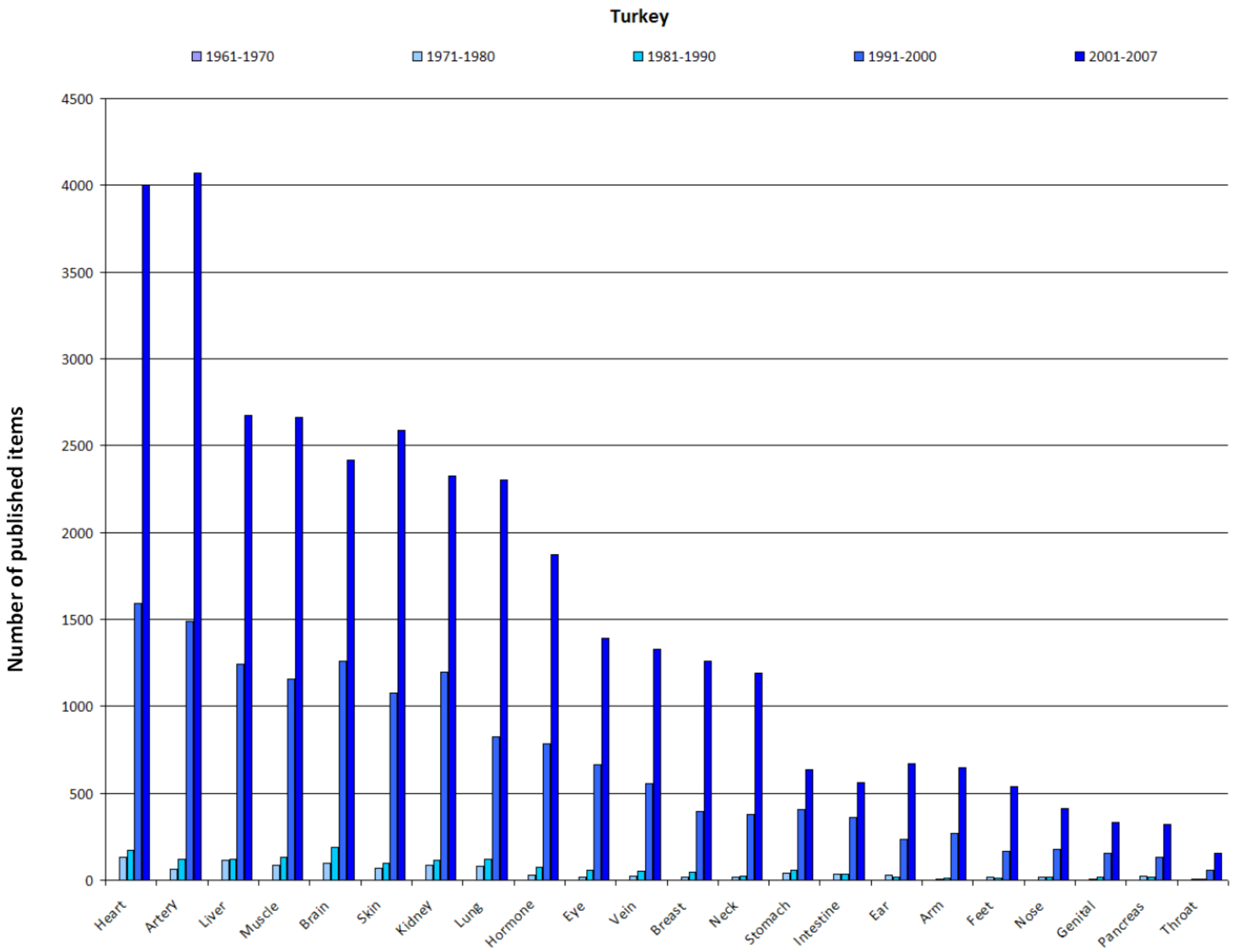

Turkey
1961-1970
1971-1980
1981-1990
1991-2000
2001-2007
Number of published items
4500
4000
3500
3000
2500
2000
1500
1000
500
0
Heart
Artery
Liver
Muscle
Brain
Skin
Kidney
Lung
Hormone
Eye
Vein
Breast
Neck
Stomach
Intestine
Ear
Arm
Feet
Nose
Genital
Pancreas
Throat

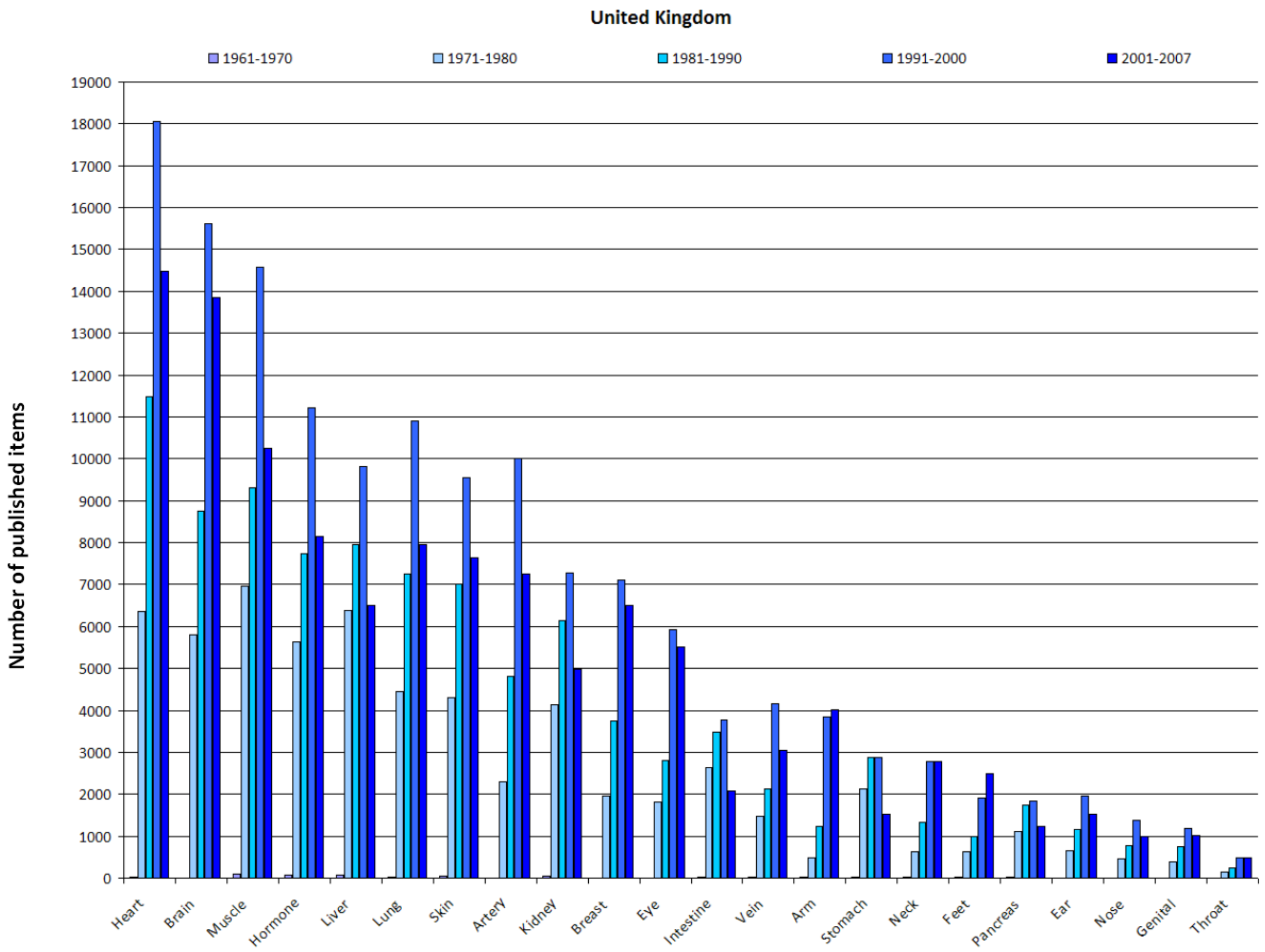
United Kingdom
1961-1970
1971-1980
1981-1990
1991-2000
2001-2007
Number of published items
19000
18000
17000
16000
15000
14000
13000
12000
11000
10000
9000
8000
7000
6000
5000
4000
3000
2000
1000
0
Heart
Brain
Muscle
Hormone
Liver
Lung
Skin
Artery
Kidney
Breast
Eye
Intestine
Vein
Arm
Stomach
Neck
Feet
Pancreas
Ear
Nose
Genital
Throat

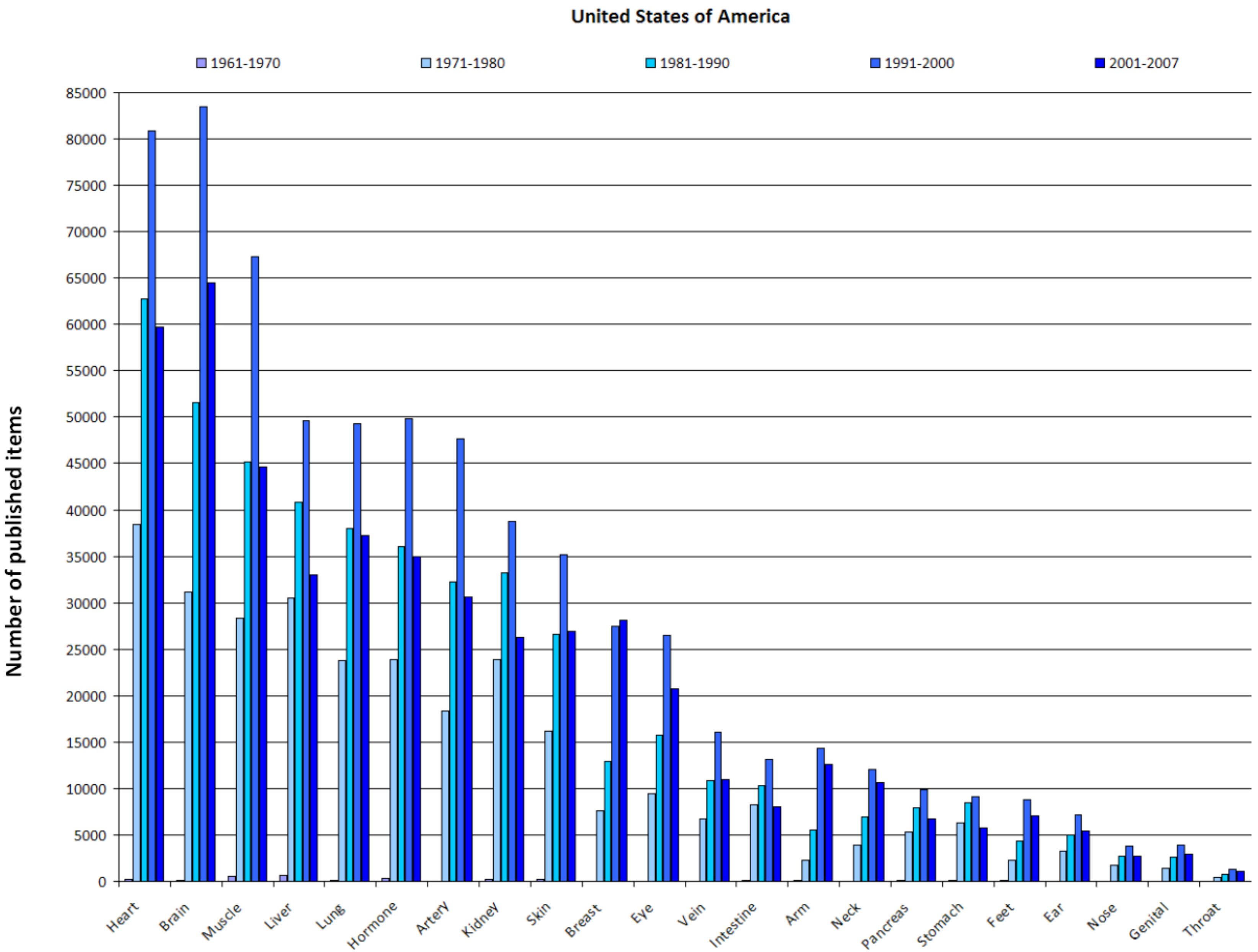
United States of America
1961-1970
1971-1980
1981-1990
1991-2000
2001-2007
Number of published items
85000
80000
75000
70000
65000
60000
55000
50000
45000
40000
35000
30000
25000
20000
15000
10000
5000
0
Heart
Brain
Muscle
Liver
Lung
Hormone
Artery
Kidney
Skin
Breast
Eye
Vein
Intestine
Arm
Neck
Pancreas
Stomach
Feet
Ear
Nose
Genital
Throat
